# Temporal relationship between vasopressor and sedative administration and cerebrovascular response in traumatic brain injury: a time-series analysis

**DOI:** 10.1186/s40635-023-00515-5

**Published:** 2023-05-29

**Authors:** Logan Froese, Alwyn Gomez, Amanjyot Singh Sainbhi, Nuray Vakitbilir, Izabella Marquez, Fiorella Amenta, Kevin Y. Stein, Frederick A. Zeiler

**Affiliations:** 1 Biomedical Engineering, Price Faculty of Engineering, University of Manitoba, Winnipeg, MB Canada; 2 Section of Neurosurgery, Department of Surgery, Rady Faculty of Health Sciences, University of Manitoba, Winnipeg, MB Canada; 3 Department of Human Anatomy and Cell Science, Rady Faculty of Health Sciences, University of Manitoba, Winnipeg, Canada; 4 Undergraduate Engineering, Price Faculty of Engineering, University of Manitoba, Winnipeg, Canada; 5 Undergraduate Medical Education, Rady Faculty of Health Sciences, University of Manitoba, Winnipeg, Canada; 6 Division of Clinical Neuroscience, Karolinska Institutet, Stockholm, Sweden; 7 Division of Anaesthesia, Department of Medicine, Addenbrooke’s Hospital, University of Cambridge, Cambridge, UK; 8 Centre on Aging, University of Manitoba, Winnipeg, Canada

**Keywords:** Autoregulation, Cerebrovascular reactivity, Sedative drugs, Vasopressors

## Abstract

**Background:**

Although vasopressor and sedative agents are commonly used within the intensive care unit to mediate systemic and cerebral physiology, the full impact such agents have on cerebrovascular reactivity remains unclear. Using a prospectively maintained database of high-resolution critical care and physiology, the time-series relationship between vasopressor/sedative administration, and cerebrovascular reactivity was interrogated. Cerebrovascular reactivity was assessed through intracranial pressure and near infrared spectroscopy measures. Using these derived measures, the relationship between hourly dose of medication and hourly index values could be evaluated. The individual medication dose change and their corresponding physiological response was compared. Given the high number of doses of propofol and norepinephrine, a latent profile analysis was used to identify any underlying demographic or variable relationships. Finally, using time-series methodologies of Granger causality and vector impulse response functions, the relationships between the cerebrovascular reactivity derived variables were compared.

**Results:**

From this retrospective observational study of 103 TBI patients, the evaluation between the changes in vasopressor or sedative agent dosing and the previously described cerebral physiologies was completed. The assessment of the physiology pre/post infusion agent change resulted in similar overall values (Wilcoxon signed-ranked *p* value > 0.05). Time series methodologies demonstrated that the basic physiological relationships were identical before and after an infusion agent was changed (Granger causality demonstrated the same directional impact in over 95% of the moments, with response function being graphically identical).

**Conclusions:**

This study suggests that overall, there was a limited association between the changes in vasopressor or sedative agent dosing and the previously described cerebral physiologies including that of cerebrovascular reactivity. Thus, current regimens of administered sedative and vasopressor agents appear to have little to no impact on cerebrovascular reactivity in TBI.

**Supplementary Information:**

The online version contains supplementary material available at 10.1186/s40635-023-00515-5.

## Background

As the foundation of current intensive care unit (ICU) guidelines, vasopressor and sedative agents are commonly administered in the acute phase after a moderate or severe traumatic brain injury (TBI) given their ability to regulate and mediate extreme levels of patient physiology. It is common for vasopressor agents to be used to maintain cerebral perfusion pressure (CPP) guideline-based targets of 60 to 70 mmHg [1], with sedative agents used to mediate intracranial pressure (ICP) and suppress cerebral metabolic demand [1–3]. However, despite their common use within the clinical setting of an ICU, the full physiologic impact of such vasopressors and sedative agents on the multi-modal cerebral physiologic response is quite limited. Furthermore, the role that such agents have on cerebrovascular reactivity, particularly after TBI is important.

In recent literature, cerebrovascular reactivity has gained extensive interest given that it has been shown to be a new independent factor associated with poor patient outcome after TBI [4–9]. Currently, cerebrovascular reactivity is measured continuously using the pressure reactivity index (PRx; a correlation between ICP and systemic blood pressure (ABP)) [10–12], though other methods of determining cerebrovascular reactivity have been proposed [13–18]. Using such methods, cerebrovascular reactivity has emerged as a unique monitored metric that is associated with poor patient outcome and mortality [7–9, 12]. In parallel, there has been limited overall improvement in TBI outcomes in moderate and severe cohorts related to guideline-based interventions over the past 25 years [7]. This is associated with unchanged insult burden related to impaired cerebrovascular reactivity despite changes in guideline-based interventions over this period. This has led to interest in utilizing cerebrovascular reactivity monitoring to generate individualized targets for critical care [7, 19–21]. These include the optimal cerebral perfusion pressure (CPPopt) [19, 20, 22, 23], the optimal bispectral index (BISopt) [21, 24], and individual ICP [25–28] as personal identifiers of overall patient state.

In order for these above-mentioned methods to be effectively utilized, there needs to be a comprehensive understanding of the impact of current critical care therapeutic interventions on cerebrovascular reactivity. Our current understanding of guideline-based therapeutics, including that of sedative and vasopressor agents, is limited mostly to large time aggregated data [7, 29–31]. It should be noted that some sedative agents have dual actions as both analgesics and anesthetics. Though in most of the literature, these drugs were administered for their anesthetic roles in induced deep states of sedation in combination with other purely anesthetic agents for the goal of improved ICP/CPP control. These studies utilize broad averages of individualized physiologic and treatment data which results in a lack of temporal resolution that is dependent on daily treatment measures. Thus, there is a limited moment-by-moment physiological understanding of the role that such agents have on systemic and cerebral physiology.

Such analyses require the use of time-linked pharmacologic data with high-frequency cerebral physiology and the leveraging of time-series methodologies to properly assess the impact of dose changes in vasopressor and sedative agents on continuously derived metrics of cerebrovascular reactivity. These datasets are often rare and difficult to come by. As such, this study aimed to assess the influence of commonly administered sedative agents; including midazolam, propofol, fentanyl, and ketamine; as well as vasopressor agents of norepinephrine, phenylephrine, and vasopressin on cerebrovascular reactivity, utilizing the prospectively maintained Winnipeg Acute TBI Database.

## Materials and methods

### Study design

We retrospectively reviewed our prospectively maintained TBI database from the Winnipeg Acute TBI Laboratories, at the University of Manitoba. From this, patients with archived high-frequency digital physiology (ICP and ABP) and treatment data pertaining to vasopressor (norepinephrine, vasopressin, and phenylephrine) or sedative (ketamine, propofol, fentanyl, and midazolam) agent administration were included. Regarding ketamine and fentanyl, though these are analgesic medications with sedative properties, for this cohort they were administered for the purpose of induction of deep sedation for the goal of ICP/CPP control. All patients included in this database were aged 17 or older and suffered a moderate-to-severe TBI, requiring admission to the surgical intensive care unit (SICU) for invasive ICP monitoring. Patients received treatment according to the Brain Trauma Foundation (BTF) guidelines (i.e., CPPopt was not targeted in this cohort) [1]. A total of 103 patients were identified, having pharmacologic agent information paired with cerebral physiologic data. Work here is similar to that done previously by our group [30, 32–34].

### Ethics

Data were collected following full approval by the University of Manitoba Health Research Ethics Board (H2017:181, H2017:188, B2018:103, H2020:118) and the Health Sciences Centre Research Impact Committee. These are renewed on an annual basis, reconfirmed in 2022. Procedures were followed in accordance with the ethical standards of the responsible committee for human experimentation and with the Helsinki Declaration of 1975.

### Patient data collection

High-frequency ABP, ICP, and regional brain tissue oxygen saturation (rSO_2_) data were collected (though it should be noted that not all patients had rSO_2_ measured [*n* = 87]). ABP was obtained through arterial lines connected to pressure transducers zeroed at the level of the tragus (Baxter Healthcare Corp. CardioVascular Group, Irvine, CA) [35]. ICP was acquired via an intra-parenchymal strain gauge probe (Codman ICP MicroSensor; Codman & Shurtlef Inc., Raynham, MA), placed in the frontal lobe, or via an extraventricular drain (EVD). rSO_2_ was measured using NIRS regional oximetry of the left and right frontal lobes (Covidien INVOS 5100C).

All signals were recorded using digital data transfer or digitized via an A/D converter (DT9803/DT9804/DT9826; Data Translation, Marlboro, MA) and, where appropriate, sampled at a frequency of 100 Hz using ICM + software (Cambridge Enterprise Ltd., Cambridge, UK, http://icmplus.neurosurg.cam.ac.uk). Signal artifacts were removed using both manual and automated methods prior to further processing or analysis, identical to past work by our lab [36–39]. The EVD ICP (*n* = 4) signals had their opening artifacts and other erroneous data cleaned through manual inspection by a trained clinician.

### Signal processing

Signal processing work was done with ICM + or R statistical software (R Core Team (2019). R: A language and environment for statistical computing. R Foundation for Statistical Computing, Vienna, Austria. URL https://www.R-project.org/). ABP and ICP were decimated over a 10-s, non-overlapping moving average filter to get MAP and ICP. CPP = MAP – ICP. PRx was derived as a Pearson correlation between 30 consecutive 10-s windows of ICP and MAP, updated every minute [13–15]. The pulse amplitude of ICP (AMP) was derived using Fourier analysis of the ICP pulse waveform [16, 40]. Pulse amplitude index (PAx) was derived as the correlation between slow waves of AMP and MAP [16, 40], and RAC was derived as the correlation between slow-waves of AMP and CPP [17]. COx_a is a minimally invasive measure derived using the standard Pearson correlation between 30 consecutive 10-s windows of MAP and rSO_2_, updated every minute to give COx_R_a and COx_L_a for the right and left side, respectively[18]. PRx, PAx, RAC, and COx_a are all surrogate measures of cerebrovascular reactivity that range from -1 to 1[13–18]. Higher values indicate more impaired cerebrovascular reactivity, while values below about 0 indicate intact cerebrovascular reactivity[5, 8, 18, 41].

Using the date and time stamp for each minute-by-minute data point, daily summaries were derived for all days after injury for each patient, producing:

1. mean ICP, % time with ICP above 20 and 22 mmHg—extracted from the BTF guidelines [3].
2. mean CPP, % time with CPP below 60 mmHg and above 70 mmHg—extracted from the BTF guidelines [1, 5].
3. mean PRx, % time with PRx above 0, + 0.25, and + 0.35 literature-defined thresholds [5, 8].
4. mean PAx, % time with PAx above 0, and + 0.25 literature-defined thresholds [8].
5. mean RAC, % time with RAC above − 0.10, and − 0.05 literature-defined thresholds [8].
6. mean COx_a, % time with COx_a above 0, and + 0.30 (both right and left side) current literature-highlighted thresholds [18, 41, 42].

### Statistical analysis

Descriptive summary statistics for the patient population are provided in Table 1. Alpha for statistical significance was set at 0.05, with Bonferroni correction applied for multiple comparisons. Boxplots, error-bar plots, and a locally estimated scatterplot smoothing (LOESS) plot were used to aid in the description of the data. The statistical analysis was split into five phases:

A. Evaluation of continuously infused drugs visually through boxplots and locally estimated scatterplot smoothing (LOESS).
B. Evaluation of physiology pre/post each dose change.
C. Dichotomize the data based on age (< 60 years and > 60 years) and sex (male and female).
D. Using latent profile analysis (LPA) to distinguish any underlying groups.
E. Vector space modeling of variables using Granger causality and impulse response function testing.

#### Boxplots of continuous infusion changes

Using locally estimated scatterplot smoothing (LOESS) and boxplots, the impact of different dosages of the infusion agents on the given physiological measures could be compared. These include MAP, ICP, CPP, PRx, PAx, RAC, and COx_a. For the derivation of the LOESS plots, the continuous agents’ infusion rates were paired with their respective minute-by-minute time-stamped physiology, thus for every minute there was physiology and indicated infusion rate. This data was then grouped for all desired continuous infusion agents over the entire dataset to give one LOESS plot per agent per physiology.

#### Evaluation of all infusions

For each infused agent, we identified all times where its infusion rate was changed or a bolus dose was given, and indexed the date, time, infusion rate, and all physiological variables data. Then, the physiology both pre-/post infusion rate change was assessed, with a 30-min window pre/post dose and a 30-min delay. Thus, each change used 90 min of data (this allowed all agents to reach full onset response, and was taken from our previous work) [32]. Any time window that had less than 50% of the data was discarded from the study. For each window, the grand mean and the percent time over the given thresholds was found for each infusion rate change. Note, the bolus and continuous infusions were separated for all analyses. Finally, a Wilcoxon signed-ranked test was performed between the two-window datasets with Bonferroni used to adjust for multiple comparisons.

The analyzed physiological variable thresholds were taken from previously indicated guideline or literature-based thresholds and were previously listed, as referenced above.

#### Dichotomization analysis

The data were then further categorized into subgroups based on demographic profiles. Evaluation of the subgroups included only age < 60 vs. age ≥ 60 and sex, based on past work [33, 43]. Like the full group analysis, for each comparison, a Wilcoxon signed-ranked test was performed between the pre- and post-windows with Bonferroni correction to adjust for multiple comparisons.

#### Latent profile analysis

Given the relatively large number of infusions for norepinephrine and propofol, we performed LPA for these agents to determine if any underlying unique physiological relationships between individual responses exist. For each dose change, the pre/post window’s physiological data were subtracted to get the delta change of the variable overtime. Thus, for MAP, ICP, CPP, AMP, rSO_2_, PRx, PAx, RAC, and COx_a, there was a single delta change value per change in dose of agent. With this linked data, a generic LPA was performed as outlined by tidyLPA [44]. As summary, LPA uses a specified number of clusters to categorize each data point into the optimal cluster. These optimal clusters are selected by minimizing the overall variance within each cluster and over the whole data. To select the optimal number of clusters, we leveraged Bayesian information criteria (BIC) which balances model fit and overfitting by selecting the lowest BIC model. With the optimal number of clusters selected an LPA was optimized and shown.

LPA was performed between increase/decrease in norepinephrine/propofol of:

1. Physiological variables—MAP/ICP/CPP/AMP/rSO_2_.
2. Cerebrovascular reactivity measures—PRx/PAx/RAC/COx_R_a/COx_L_a.

#### Vector space time-series assessment

Vector space modeling was completed for the continuous infusion of norepinephrine and propofol, as well as bolus doses of fentanyl (given that these had the most amount of data), utilizing 2 methods: Granger causality testing and impulse response testing. Initially, the 30-min windows pre/post drug infusion change/bolus dose for all variables was tested for stationarity using the Augmented Dicky Fuller (ADF) and Kwiatkowski–Phillips–Schmidt–Shin (KPSS). Since stationarity was not confirmed for all models, we performed a first-order difference on the initial signal. After stationarity was confirmed, basic Granger testing was performed on the bidirectional relationships between ABP and ICP/rSO_2_/AMP, and CPP and AMP for both the pre/post windows of time using previously outlined methods (these relationships correspond to PRx, COx_a, PAx, and RAC measures) [45, 46]. An autoregressive order of 4 for the vector models was chosen based on previous cerebral physiologic literature [45, 46]. The final p-values were compared and the model whose values were significant (*p* < 0.05) were denoted. For those models whose p-values were bidirectionally significant, the higher f statistic was used to denote the optimal directional relationship. From these any that had a denoted directional impact difference from the pre to post window was tallied to give a total percent from which the drug impacted the overall Granger causality relationship (i.e., did the drug impact directionality).

To perform an impulse response function (IRF) analysis, the data variables of ABP/ICP/rSO_2_/AMP and CPP/AMP were initially modeled for each 30-min window using a vector autoregressive moving average (VARMA). However, since many models did not converge, the moving average component was dropped and a vector autoregressive (VAR) structure using 4 lag components was found (as documented by previous literature) [45, 46]. These VAR models were then assessed directionally using IRF to evaluate the impact of ABP on ICP/rSO_2_/AMP and CPP on AMP, and vice versa. These impulse responses were saved and then, for all data, one final overall IRF was found from the median and interquartile range of the collected responses, resulting in one plot for each agent change/window/variable pulse.

## Results

The median age was 42 years (interquartile range; IQR: 28–57 years), with 87 (82.9%) patients being males. For dosing distribution of continuous infusions, see Additional file 1: Appendix A. These TBI demographics are in keeping with normal TBI cohorts. The core patient demographics can be seen in Table 1.

**Table 1:** Patient demographics and clinical characteristics for entire cohort

Characteristics	Number (%) or median (interquartile range)
*n* (patients)	103
Age (years)	42 (28–57)
Sex (Male)	87 (82.9%)
Admission GCS	7 (4–8)
Admission GCS-motor	4 (2–5)
Pupillary light reflex	
Bilateral reactive	63 (61%)
Unilateral unreactive	23 (22%)
Bilateral unreactive	17 (17%)
Pre-hospital hypoxia	38 (37%)
Pre-hospital hypotension	12 (12%)
Subarachnoid hemorrhage	101 (97%)
Epidural hematoma	11 (11%)
Marshall classification category of 1st head-CT	
V	50 (49%)
IV	20 (20%)
III	30 (29%)
II	3 (2%)

Demographic information of the patient cohort. *CT* computed tomography, *GCS* Glasgow Coma Score, *GOSE* Glasgow outcome scale-extended

### Continuous infusions physiological associations

**Fig. 1:**
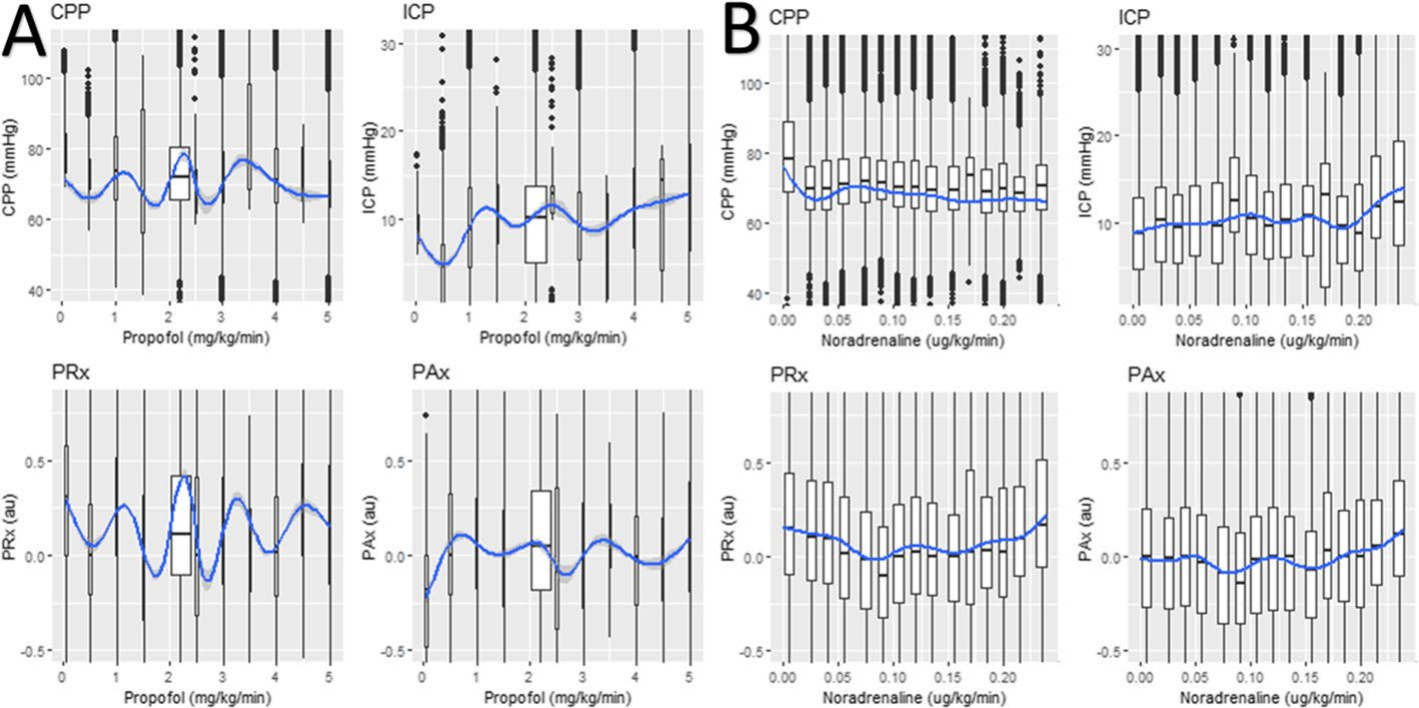
Boxplots with LOESS curve—propofol. The figure shows boxplots of different dose amounts of propofol/norepinephrine and their associations with different physiological variables (i.e., minute-by-minute data paired with continuous infusion rate). “**A**” is propofol and “**B**” is norepinephrine with the key physiologies minute data, highlighting the minimal impact of these agents on physiology. *au* arbitrary units, *CPP* cerebral perfusion pressure, *hr* hour, *ICP* intracranial pressure; kg, kilogram, *PAx* pulse amplitude index, *PRx* pressure reactivity; mg, milligram, *mmHg* millimeter of mercury, *ug* microgram

In general, there was a limited relation between the continuously infused agents and any cerebrovascular reactivity measure (PRx/PAx/RAC/COx). Norepinephrine did show some significance with respect to its impact on CPP (see Additional file 1: Appendix D). Figure 1 shows the boxplots of propofol and noradrenaline, other data for this analysis can be found in Additional file 1: Appendix B and C.

### Overall dose response

**Fig. 2:**
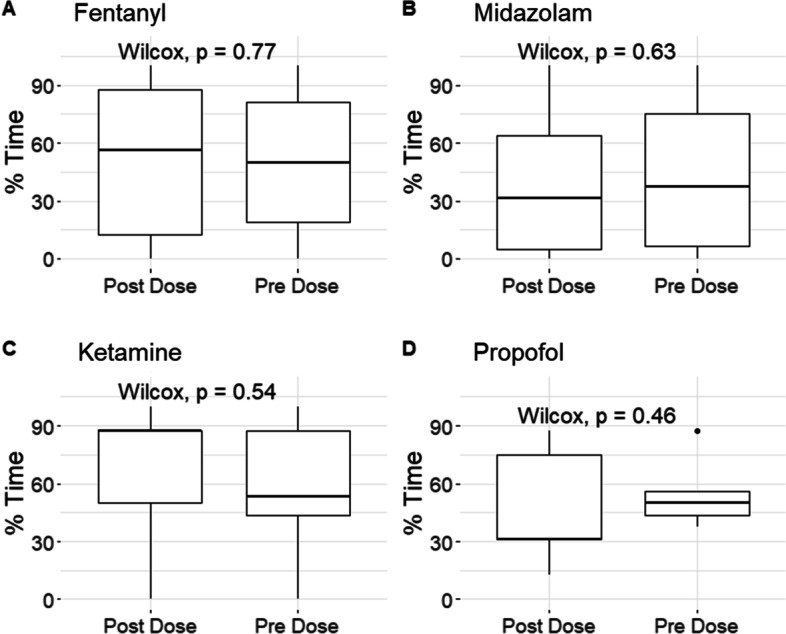
Box plots for bolus doses—% time PRx > 0. Figure shows boxplots of different bolus agents (only bolus infusions), with the Wilcoxon signed-ranked value between the pre- vs post-doses. “**A**” is fentanyl, “**B**” is midazolam, “**C**” is ketamine and “**D**” is propofol, with each drug there is a limited impact from a bolus dose on the change in physiology. *au* arbitrary units, *PRx* pressure reactivity, *mins* minutes

Overall, there was little-to-no impact of changes in bolus or continuous infusions of drugs (vasopressors or sedatives) on continuously measured metrics of cerebrovascular reactivity. Figure 2 and Additional file 1: Appendix D show the bolus and continuous infusion agents for the full monitoring time.

### Dichotomization based on age and sex

No incremental dose changes or bolus doses demonstrated any influence on cerebrovascular reactivity or cerebral physiology, regardless of group adjustment based on age or biological sex. Subcategorization of the data by age and sex can be found in Additional file 1: Appendix E–H.

### Latent profile analysis

Through the LPA of norepinephrine/propofol change in dose vs mean variable, some interesting results deserve highlighting. First, in general the cerebrovascular reactivity measures of PRx/PAx/RAC/COx had similar locations of clusters across these variables. Moreover, since there were only 2 clusters for propofol, it is quite apparent that the impaired cerebrovascular reactivity values (> 0) were grouped together. The main physiological variables of MAP/ICP/AMP/CPP/rSO_2_ appeared to cluster together on the extreme ends of the groups. All of this work is still limited and thus should be considered preliminary in nature, with the dataset currently available but still insufficient to make robust comments on the nature of individual patterns (all of this can be seen in Additional file 1: Appendix I and J).

### Time series analysis

#### Granger causality testing

The comparison of the Granger causality results between the pre/post window for norepinephrine, propofol, and fentanyl were tallied and given as a percent of total number that stayed the same (Table 2). Given that the individual dose changes had a limited impact on Granger directionality (> 95% were unaffected by the drug change).

**Table 2:** Granger causality testing

Agent	Mean dose change	ICP and ABP	rSO2_L and ABP	rSO2_R and ABP	AMP and ABP	AMP and CPP
Norepinephrine	Increase	99.6	99.2	99.8	99.6	99.5
Norepinephrine	Decrease	99.8	99.6	99.7	99.8	99.6
Propofol	Increase	99.4	98.9	99.4	99.2	99.2
Propofol	Decrease	100	98.9	99.6	99.1	99.6
Fentanyl	Increase	98.9	98.9	98.9	98.9	98.9
Fentanyl	Decrease	98.9	98.4	98.9	98.9	98.9
Fentanyl	Bolus	98.9	98.4	98.9	98.9	98.9

The table shows from Granger testing the percent that the directional relationship remained the same pre/post dose change for each type of mean dose change, window type and agent. Given the similar proportions of number in the pre/post windows for each agent, it can be seen that the impact of said agent on the physiological relationship was minimal. *ABP* arterial blood pressure, *AMP* pulse amplitude of intracranial pressure, *CPP* cerebral perfusion pressure, *ICP* intracranial pressure, *rSO2_L* regional oxygen saturation on left side, *rSO2_R* regional oxygen saturation on right side

#### Impulse response function

Given the similarity in the plots both pre/post dose change and the overall similarity in the response signals, there is limited modeled difference between these physiological pre/post drug infusion changes. Additional file 1: Appendix K–M demonstrates the impulse response function plots for norepinephrine, propofol, and fentanyl using VAR models for the impact of ABP on ICP/rSO2/AMP and CPP on AMP, and vice versa.

## Discussion

From the temporally resolved high-resolution dataset from the Winnipeg Acute TBI database, we were able to retrospectively analyze the relationships between various sedative/vasopressor agents and cerebrovascular reactivity. Although fentanyl and ketamine can be used as both analgesic and sedative medications, in this study they were administered for the purpose of sedation. To perform this evaluation, the relationships between the physiological responses and incremental medication dose change (both bolus and infusion) were assessed, employing standard statistical comparison, LPA, and time-series analyses. Some important aspects were identified through this work and need to be highlighted.

Like past studies that assessed cerebrovascular reactivity measures and vasopressor agents, cerebrovascular reactivity indices were not significantly associated with mean hourly or incremental dose changes in vasopressor agents [31, 32]. Moreover, this was the first work to assess cerebrovascular reactivity measures determined through both AMP-based measures (PAx and RAC) and rSO_2_-based measures (COx_a). Notably, these measures demonstrated a similar, non-significant relationship between vasopressors and cerebrovascular reactivity response. Thus, confirming their limited overall relationships helps to better highlight the fact that such continuously derived cerebrovascular reactivity measures remain relatively independent to current critical care therapies [7, 29, 30, 32, 47]. Moreover, this work helps confirm that there may not be a need to account for these small incremental or daily dosing changes in future studies that review cerebrovascular reactivity or other individualized physiological targets derived from cerebrovascular reactivity, including those based on different methodologies of cerebrovascular reactivity assessment. However, it must again be acknowledged that this is only the second study with a unique population dataset and thus further investigation is required.

Like vasopressor agents, sedative agents had a relatively minimal impact on cerebrovascular reactivity, despite various dosing changes or bolus doses given throughout a patient’s critical care. As noted by the limited pre/post dose change in these agents, as well as time spent above given physiological thresholds remaining consistent between both windows, PRx/PAx/RAC/COx_a all had a limited overall impact from these given agents. This included a significant number of fentanyl and propofol doses that lacked any statistical relationship, further enhancing our work and understanding [32].

The subcategorization of this data was performed in conjunction with past literature from our group that documented the association between age/sex and cerebrovascular reactivity measures. Within this study, it was documented that PRx was less reactive in older age demographics than PAx or RAC [43]. Thus, through this study, we reconfirmed that despite the effect that age may have on cerebrovascular reactivity, there is limited impact from currently utilized medication and guideline-based therapies on these measures.

Using LPA, both cerebrovascular reactivity measures and main physiological variables clustered in similar patterns on the measured responses. This clustering indicates that similar physiological variables responses are linked to similar patient profiles. Likewise, the uniform nature of the cerebrovascular reactivity clusters helps confirm that these measures are sufficiently similar as to document the same patterns. However, this work is still highly preliminary and requires larger datasets to better highlight the underlying cluster seen in these variables.

Finally using time-series relationships through Granger causality testing and impulse response function testing, there was a limited overall physiological relationship between these variables and the impact of sedative/vasopressor agents. The directional response of Granger causality between the measure physiology was not significantly impacted by the sedative and vasopressor agents. Furthermore, as can be seen by the similarity within the pre/post window impulse response for both bidirectional relationships, the individualized dose given has a limited overall impact on the resulting waveform, and ABP is a more consistent driver of other cerebral physiological variables then the other way.

## Limitations/future directions

As this study is utilizing a retrospectively maintained observational dataset that is relatively new, it lacks a robust number of both individualized patients as well as multi-center demographic profiles. Thus, this is a goal and focus of an ongoing collaborative research groups in moderate/severe TBI[9, 31, 48, 49]. Next, given the nature of this retrospective study, it is difficult to ascribe the direction of causality, whether the results are due to the limited impact from agents or that the physiological response is tapered through the use of drugs. The use of higher resolution more accurate datasets and leveraging both paired time and data collection techniques is imperative if we wish to determine this discrepancy. The use of computational driven data collection may be a desired route for future studies that wish to assess and mediate cerebrovascular reactivity [50].

Finally, though there was limited novel insights gleaned from utilizing complex time-series analysis or latent profile analysis, these methods do offer higher resolution more accurate methods of determining individualized patient response. A further application of such methods on a large dataset would be beneficial as these methods are supported by more data. Moreover, these methods are important in highlighting outliers and unique patient characteristics. Thus, utilization of such methods with demographics and pharmacodynamic profiles would better outline more unique relationships.

## Conclusions

In this study, we assessed the impact of dose changes in sedative agents (propofol, fentanyl, midazolam, and ketamine) and vasopressor agents (norepinephrine, phenylephrine, and vasopressin), determining that overall, they do not have a significant impact on multi-modal continuously measured cerebrovascular reactivity. In general, this study indicates that commonly administered sedative and vasopressor agents (given according to current critical care guidelines) do not significantly impact cerebrovascular reactivity in TBI. However, this study’s results should be considered preliminary, more work is required in order to identify the unique pharmacodynamic profiles that each drug may have.

## Supplementary Information

**Additional file 1**: **A**. Histogram distributions of continuous infusion agents. **B**. Boxplots and LOESS curves of continuous infusions—CPP/ICP/PRx/Pax. **C**. Boxplots and LOESS curves of continuous infusions—MAP/RAC/COx_R_a/COx_L_a. **D**. Infusions of all data. **E**. Age<60. **F**. Age≥60. **G**. Female. **H**. Males. **I**. Latent profile analysis—norepinephrine. **J**. Latent profile analysis—propofol. **K**. Norepinephrine impulse response functions. **L**. Propofol impulse response functions. **M**. Fentanyl impulse response functions.

## Data Availability

All data generated or analyzed during this study are included in this published article [and its supplementary information files].

## Abbreviations

ABP: Arterial blood pressure
AMP: Pulse amplitude of ICP
au: Arbitrary units
BIS: Bispectral index
BISopt: Optimal BIS index
CPP: Cerebral perfusion pressure
CPPopt: Optimal cerebral perfusion pressure
COx_a: Cerebral oximetry index from MAP
COx_a_l: Cerebral oximetry index from MAP on left side
COx_a_r: Cerebral oximetry index from MAP on right side
EEG: Electroencephalogram
GCS: Glasgow Coma Scale
Hz: Hertz
ICM+: Intensive care monitoring “plus” software
ICP: Intracranial pressure
kg: Kilogram
LOESS: Locally estimated scatterplot smoothing
MAP: Mean arterial pressure
MAPopt: Optimal MAP
mg: Milligram
min: Minute
mmHg: Millimeter of mercury
NIRS: Near-infrared spectroscopy
PAx: Pulse amplitude index
PRx: Pressure reactivity index
Sec: Seconds
rSO_2_: Regional oxygen saturation
RAC: Correlation between AMP and CPP
ug: Micrograms
VAR: Vector autoregressive
VARMA: Vector autoregressive moving average
