# Supplementary material for: Temporal relationship between vasopressor and sedative administration and cerebrovascular response in traumatic brain injury: a time-series analysis
Source: Intensive Care Med Exp. 2023 May 29;11:30. doi: 10.1186/s40635-023-00515-5 (PMC10225391; doi:10.1186/s40635-023-00515-5)
Supplement: Supplementary file 1 — Additional file 1: A. Histogram distributions of continuous infusion agents. B. Boxplots and LOESS curves of continuous infusions—CPP/ICP/PRx/Pax. C. Boxplots and LOESS curves of continuous infusions—MAP/RAC/COx_R_a/COx_L_a. D. Infusions of all data. E. Age<60. F. Age≥60. G. Female. H. Males. I. Latent profile analysis—norepinephrine. J. Latent profile analysis—propofol. K. Norepinephrine impulse response functions. L. Propofol impulse response functions. M. Fentanyl impulse response functions. [file 40635_2023_515_MOESM1_ESM.docx]

Table of Contents

Contents

[Appendix A. Histogram Distributions of Continuous Infusion Agents 3](#_Toc125449827)

[Appendix B. Boxplots and LOESS Curves of Continuous Infusions – CPP/ICP/PRx/PAx 7](#_Toc125449828)

[Appendix C. Boxplots and LOESS Curves of Continuous Infusions – MAP/RAC/COx_R_a/COx_L_a 10](#_Toc125449829)

[Appendix D. Infusions of All Data 14](#_Toc125449830)

[Appendix E. Age < 60 22](#_Toc125449831)

[Appendix F. Age >= 60 29](#_Toc125449832)

[Appendix G. Females 37](#_Toc125449833)

[Appendix H. Males 44](#_Toc125449834)

[Appendix I. Latent Profile Analysis - Norepinephrine 52](#_Toc125449835)

[Appendix I1. Decrease in Agent - MAP/ICP/CPP/AMP/rSO2_l/rSO2_r 53](#_Toc125449836)

[Appendix I2. Decrease in Agent - PRx/PAx/RAC/COx_R_a /COx_L_a 54](#_Toc125449837)

[Appendix I3. Increase in Agent - MAP/ICP/CPP/AMP/rSO2_l/rSO2_r 55](#_Toc125449838)

[Appendix I4. Increase in Agent - PRx/PAx/RAC/COx_R_a /COx_L_a 56](#_Toc125449839)

[Appendix J. Latent Profile Analysis - Propofol 57](#_Toc125449840)

[Appendix J1. Decrease in Agent - MAP/ICP/CPP/AMP/rSO2_l/rSO2_r 58](#_Toc125449841)

[Appendix J2. Decrease in Agent - PRx/PAx/RAC/COx_R_a /COx_L_a 59](#_Toc125449842)

[Appendix J3. Increase in Agent - MAP/ICP/CPP/AMP/rSO2_l/rSO2_r 60](#_Toc125449843)

[Appendix J4. Increase in Agent - PRx/PAx/RAC/COx_R_a /COx_L_a 61](#_Toc125449844)

[Appendix K. Norepinephrine Impulse Response Functions 62](#_Toc125449845)

[Appendix K1. Increase in Agent 62](#_Toc125449846)

[Appendix K2. Decrease in Agent 72](#_Toc125449847)

[Appendix L. Propofol Impulse Response Functions 82](#_Toc125449848)

[Appendix L1. Increase in Agent 82](#_Toc125449849)

[Appendix L2. Decrease in Agent 92](#_Toc125449850)

[Appendix M. Fentanyl Impulse Response Functions 102](#_Toc125449851)

[Appendix M1. Bolus 102](#_Toc125449852)

The Appendices E-H compares the pre-post dose change for various indicated physiologies. The time window is taken as the value average over a 30-minute window, with the post-window taken 30 minutes after the infusion change. Each comparison has the p-value for a Wilcox Signed ranked test, and the Bonferroni adjusted p-value. Table C is the table of all the data, and D-H are all different subdivisions of the original cohort.

# Appendix A. Histogram Distributions of Continuous Infusion Agents

*The histograms contain all continuous infusions given with the cohort. hr, hour; IU, infusion units; kg, kilogram; mg, milligram; min, minutes; ml, millilitres; ug, micrograms.*

*
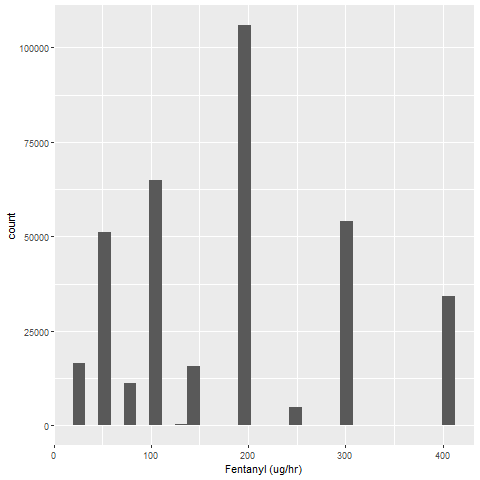

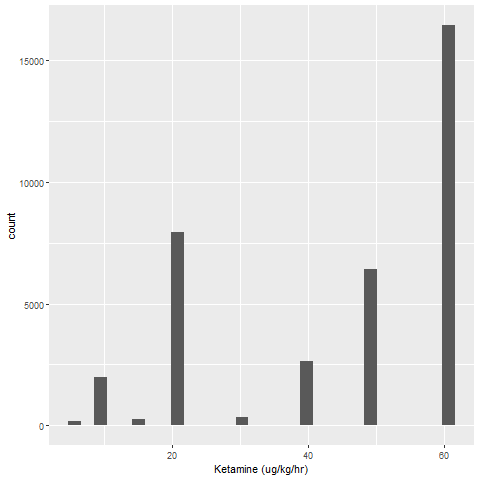
*

*
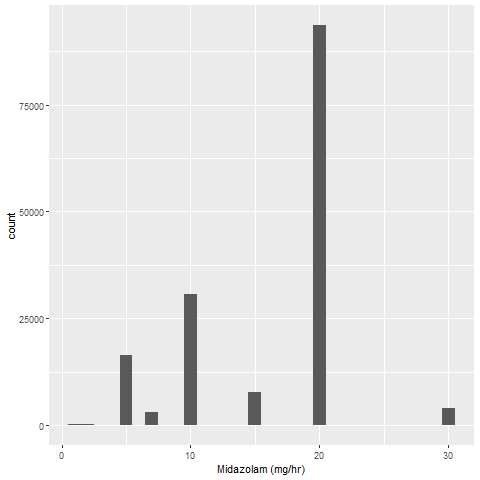

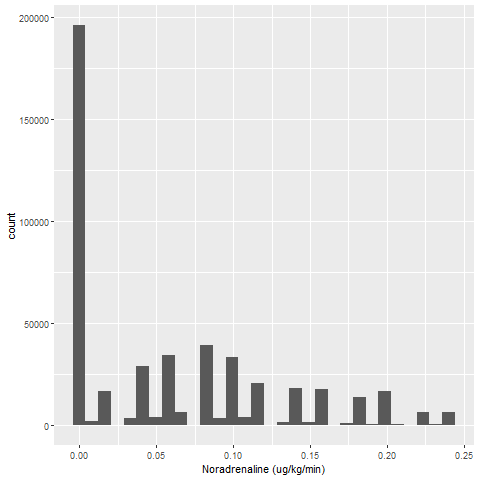

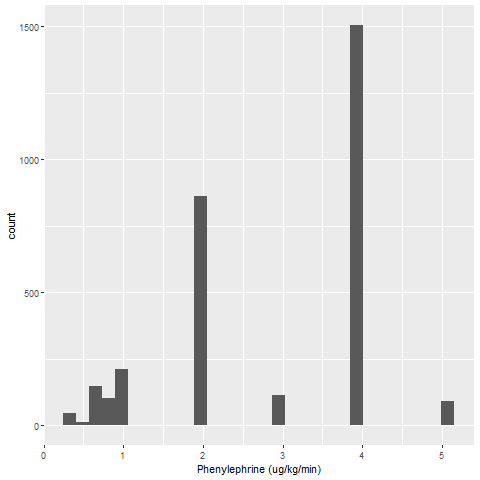

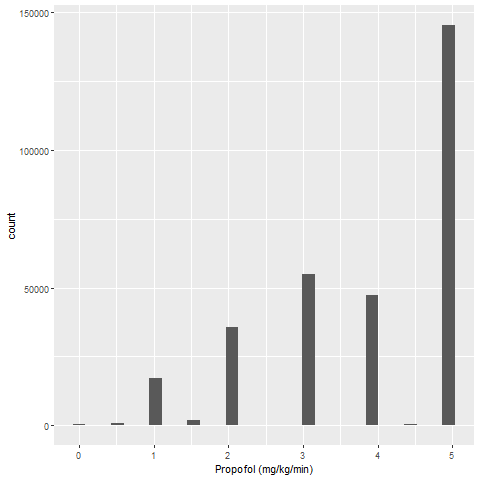
*

*
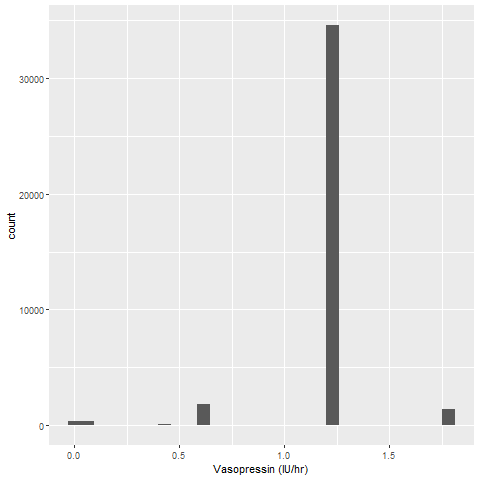
*

# Appendix B. Boxplots and LOESS Curves of Continuous Infusions – CPP/ICP/PRx/PAx


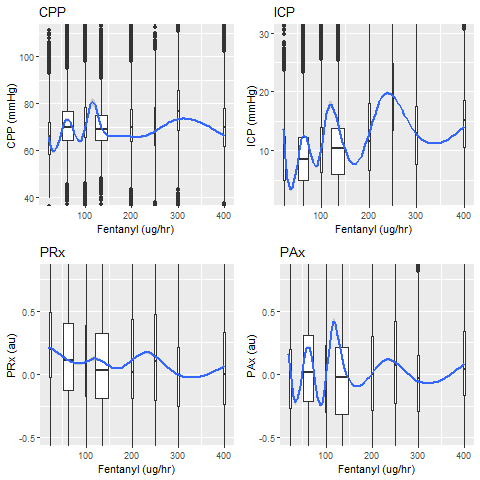

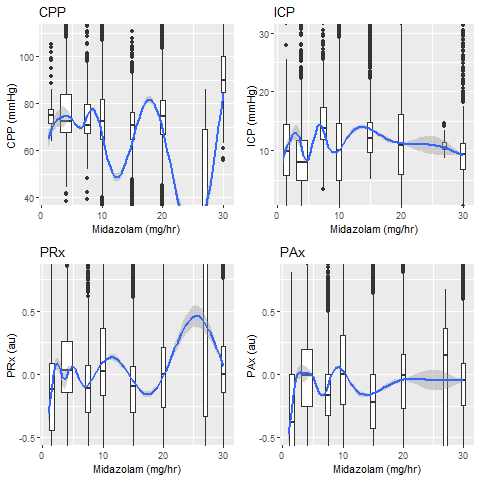


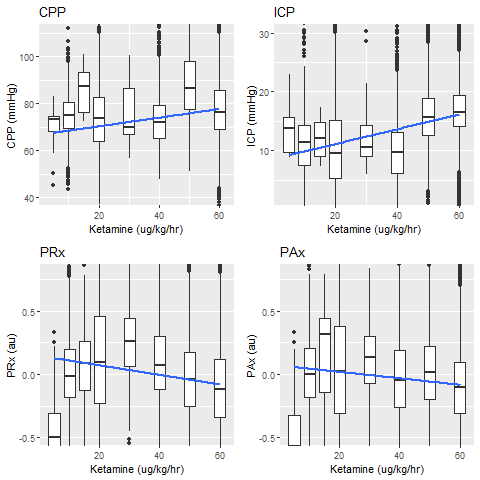

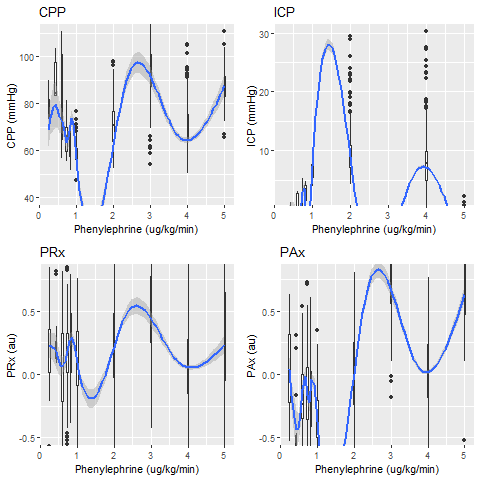


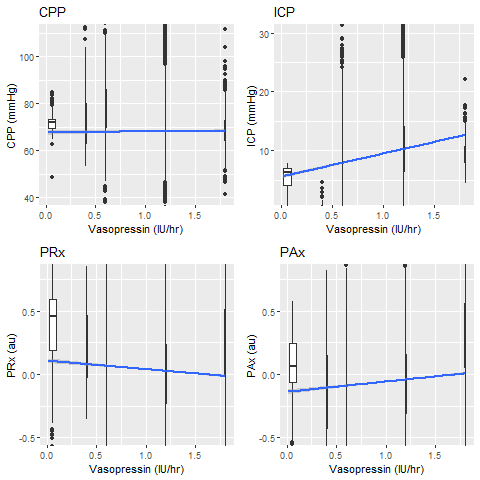


*Figure of the boxplot and LOESS curves of different dose amounts and different physiological associations. Note for these, there are limited doses thus the result will not be accurate to the global response. Au, arbitrary units; CPP, cerebral prefusion pressure; hr, hour; ICP, intracranial pressure; IU, infusion units; kg, kilogram; PAx, pulse amplitude index; PRx, pressure reactivity; mg, milligram; min, minutes; ml, millilitres; mmHg, millimeter of mercury; ug, micrograms;*

# Appendix C. Boxplots and LOESS Curves of Continuous Infusions – MAP/RAC/COx_R_a/COx_L_a


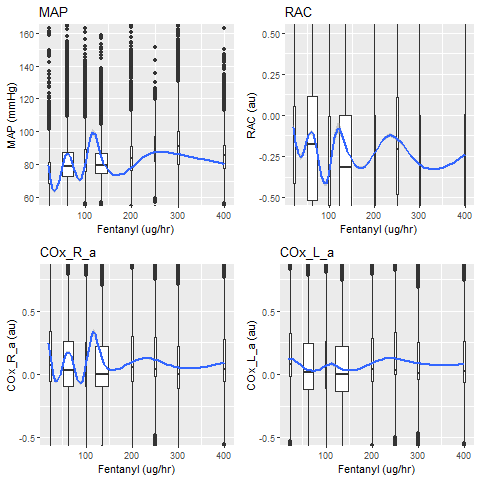

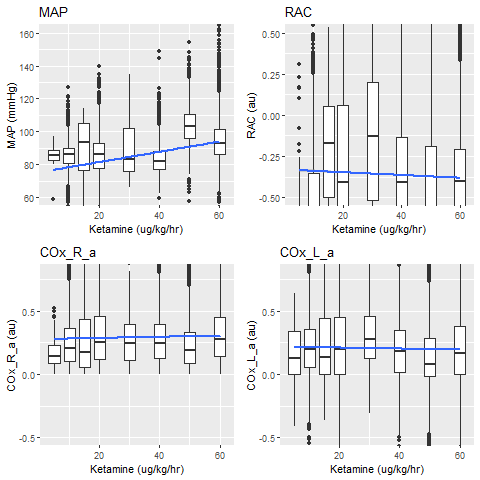


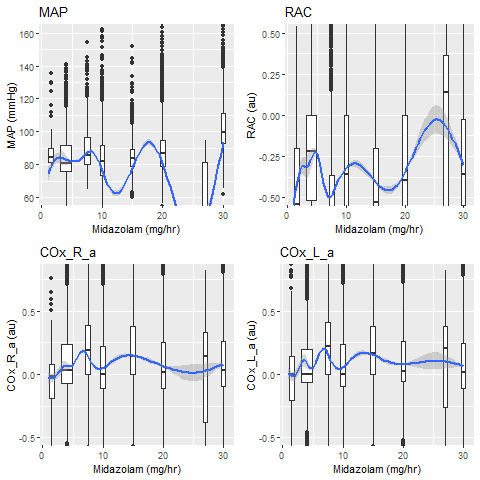

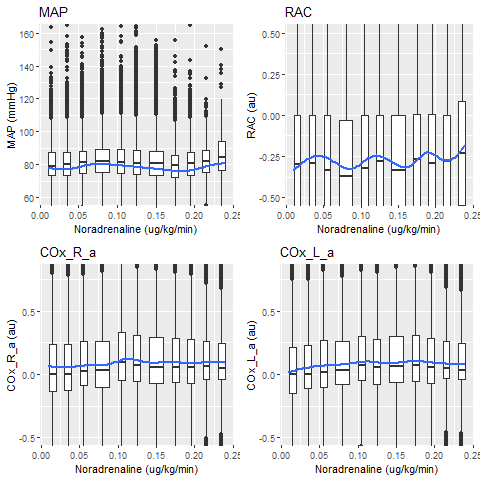

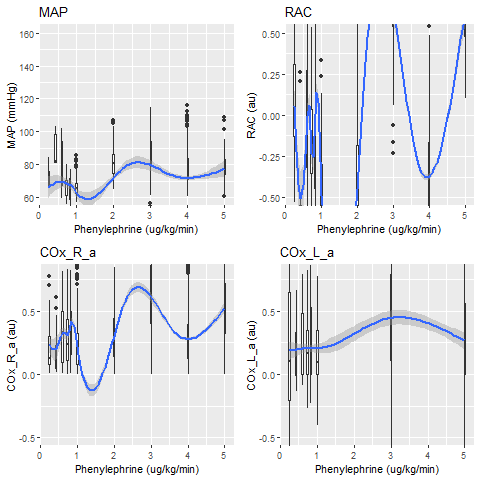

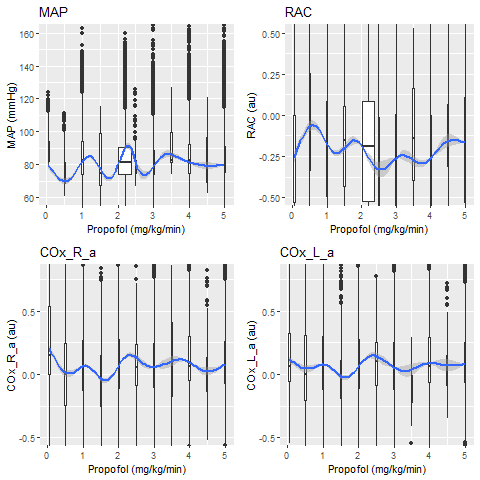

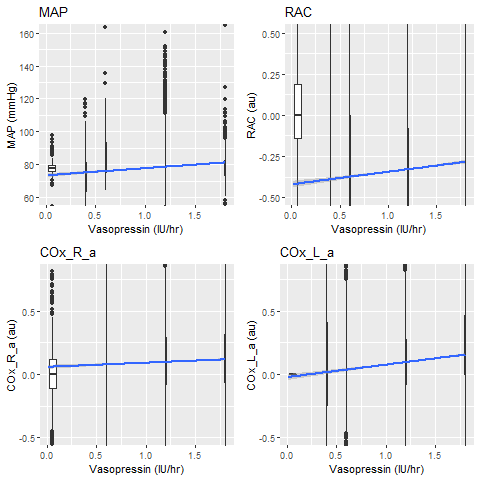


*Figure of the boxplot and LOESS curves of different dose amounts and different physiological associations. Note for these, there are limited doses thus the result will not be accurate to the global response. Au, arbitrary units; COx_L_a, cerebral oximetry index of left side using MAP; COx_R_a, cerebral oximetry index of right side using MAP; CPP, cerebral prefusion pressure; hr, hour; IU, infusion units; kilogram; MAP, mean arterial blood pressure; mg, milligram; min, minutes; ml, millilitres; mmHg, millimeter of mercury; ug, micrograms.*

# Appendix D. Infusions of All Data

The table contains all infusions given within this cohort, separated into the continuous infusion doses then the bolus doses.

| **Continuous Infusion** | | | | | | | | | | | | | | |
| --- | --- | --- | --- | --- | --- | --- | --- | --- | --- | --- | --- | --- | --- | --- |
| **Name** | **Doses** | **Mean Dose Change** | **Mean MAP** | | | | **Mean ICP** | | | | **Mean rSO2_R** | | | |
|  |  |  | **Pre Dose** | **Post Dose** | **P value** | **Adj P Value** | **Pre Dose** | **Post Dose** | **P value** | **Adj P Value** | **Pre Dose** | **Post Dose** | **P value** | **Adj P Value** |
| Fentanyl | 168 | Decrease | 75.8 (70.3-86.1) | 79.2 (72.5-89.1) | 0.0771 | 1 | 9.7 (5.44-13.6) | 9.74 (5.56-14.3) | 0.681 | 1 | 67.2 (55.7-72) | 64.9 (55.9-72) | 0.963 | 1 |
| Fentanyl | 163 | Increase | 78.8 (71.1-87.9) | 78 (69.5-86) | 0.374 | 1 | 11.8 (5.27-16.8) | 10.9 (5.52-14.6) | 0.538 | 1 | 64.2 (53.1-71.4) | 65.9 (54.4-70.2) | 0.644 | 1 |
| Ketamine | 37 | Increase | 77.5 (72.5-89.4) | 77.5 (74.3-88.9) | 0.494 | 1 | 11.4 (7.38-19.2) | 12.2 (7.09-19.9) | 0.96 | 1 | 56.2 (51.5-70.1) | 59.1 (51.2-70) | 0.923 | 1 |
| Ketamine | 13 | Decrease | 76.5 (72.3-83.1) | 76.7 (71-84) | 0.943 | 1 | 8.89 (4.68-14.4) | 9.96 (6.57-18.3) | 0.662 | 1 | 69.2 (61.3-73.8) | 69 (62.4-74.6) | 0.801 | 1 |
| Midazolam | 83 | Increase | 75.4 (69.2-86.2) | 77.6 (72.2-85.7) | 0.491 | 1 | 11.4 (8.75-14.3) | 10.6 (7.06-14.9) | 0.149 | 1 | 60 (40.6-67.6) | 59.6 (38.7-67.4) | 0.81 | 1 |
| Midazolam | 73 | Decrease | 79.1 (70.3-97) | 80.8 (70.5-96.3) | 0.768 | 1 | 13.4 (10.3-17.7) | 11.6 (9.51-17.4) | 0.609 | 1 | 55.9 (16.7-67.7) | 55.1 (0-67.2) | 0.803 | 1 |
| Norepinephrine | 1553 | Decrease | 79.1 (71.6-87.4) | 77.9 (71.3-85.7) | 0.0175 | 0.456 | 10.3 (6.27-14.3) | 10.6 (6.35-14.6) | 0.44 | 1 | 61.1 (53-68.7) | 60.8 (53-68.4) | 0.658 | 1 |
| Norepinephrine | 1303 | Increase | 76 (69.5-83.9) | 76.5 (70.1-84.6) | 0.19 | 1 | 10.8 (6.27-15.8) | 10.6 (6.37-15.2) | 0.654 | 1 | 61 (53.5-68.2) | 61 (53.5-68.6) | 0.777 | 1 |
| Phenylephrine | 15 | Increase | 59.8 (29.2-67.2) | 59.9 (29.7-64.5) | 0.9 | 1 | 5.27 (0.492-12) | 6.02 (1.34-14.9) | 0.902 | 1 | 60.8 (27.8-79.7) | 60.5 (26.8-78.6) | 0.803 | 1 |
| Phenylephrine | 11 | Decrease | 71.8 (64-81.5) | 67.5 (62.2-73.3) | 0.347 | 1 | 7.65 (0.879-11.1) | 7.97 (4.66-10.9) | 0.74 | 1 | 71.3 (52.8-79.2) | 71.4 (54.1-80.2) | 0.799 | 1 |
| Propofol | 409 | Decrease | 77.9 (70.5-85.9) | 78.6 (71.1-86.9) | 0.265 | 1 | 8.63 (4.33-12.9) | 9.02 (4.38-13.4) | 0.509 | 1 | 64.5 (55.5-70.8) | 64 (55.7-70.7) | 0.962 | 1 |
| Propofol | 301 | Increase | 79 (69.5-89.9) | 77.7 (69.3-87.3) | 0.133 | 1 | 9.35 (4.11-13.4) | 8.99 (4.64-13) | 0.802 | 1 | 66.2 (57.2-71.4) | 65.8 (57.1-71.3) | 0.552 | 1 |
| Vasopressin | 58 | Decrease | 78.4 (70.1-87.2) | 78.6 (69.6-88.1) | 0.874 | 1 | 6.44 (4.15-14.7) | 8.54 (5.27-16.6) | 0.308 | 1 | 54.6 (38.6-64.8) | 56.5 (42.1-65.5) | 0.825 | 1 |
| Vasopressin | 44 | Increase | 73.2 (69-84.7) | 72.3 (68-83.9) | 0.537 | 1 | 11.7 (6.13-18.9) | 11.2 (5.97-15) | 0.493 | 1 | 56.3 (0-65.2) | 55.4 (0-65.5) | 0.792 | 1 |
| **Name** | **Mean Dose Change** | **Mean rSO2_L** | | | | **Mean CPP** | | | | **Mean AMP** | | | |  |
|  |  | **Pre Dose** | **Post Dose** | **P value** | **Adj P Value** | **Pre Dose** | **Post Dose** | **P value** | **Adj P Value** | **Pre Dose** | **Post Dose** | **P value** | **Adj P Value** |  |
| Fentanyl | Decrease | 65.7 (58.1-73.2) | 65.1 (58.2-73.2) | 0.912 | 1 | 67.9 (60.4-75.8) | 69.8 (62.5-77.5) | 0.175 | 1 | 1.59 (1.09-2.12) | 1.64 (1.14-2.17) | 0.431 | 1 |  |
| Fentanyl | Increase | 67.1 (59.9-73.6) | 67.1 (58.6-73.1) | 0.801 | 1 | 66.9 (61.8-74.4) | 67.2 (60.1-72.8) | 0.467 | 1 | 1.79 (1.15-3.11) | 1.72 (1.05-3.04) | 0.78 | 1 |  |
| Ketamine | Increase | 64.5 (0-68.1) | 65.3 (0-67.4) | 0.746 | 1 | 65.1 (54.2-80.8) | 66.3 (60.1-76.6) | 0.699 | 1 | 1.87 (1.09-3.9) | 1.36 (1.12-4.6) | 0.969 | 1 |  |
| Ketamine | Decrease | 69.3 (68.5-74.2) | 69.6 (69.2-75.6) | 0.57 | 1 | 68.1 (64.8-76.2) | 67.2 (60.3-71.4) | 0.576 | 1 | 1.78 (1.59-2.92) | 1.71 (1.38-4.25) | 0.884 | 1 |  |
| Midazolam | Increase | 63.1 (58.7-70.1) | 64 (59.3-70.3) | 0.874 | 1 | 63.3 (58.5-72.3) | 67.2 (59.9-74.3) | 0.117 | 1 | 2.19 (1.22-4.8) | 2.01 (1.18-4.32) | 0.403 | 1 |  |
| Midazolam | Decrease | 63.3 (58.1-66.2) | 63 (52.8-65.8) | 0.517 | 1 | 69.9 (59.8-79) | 70.6 (59.8-80.2) | 0.84 | 1 | 2.05 (1.03-4.02) | 1.42 (0.987-4.62) | 0.933 | 1 |  |
| Norepinephrine | Decrease | 64.1 (56.6-70.8) | 64.1 (56.2-70.4) | 0.635 | 1 | 69 (61.4-76) | 67.8 (60.8-74.2) | 0.00331 | 0.0861 | 1.89 (1.12-3.29) | 1.85 (1.12-3.2) | 0.841 | 1 |  |
| Norepinephrine | Increase | 63.4 (55.9-70.7) | 63.4 (56.1-70.7) | 0.808 | 1 | 65.1 (59.4-70.8) | 65.6 (59.8-71.8) | 0.064 | 1 | 2.01 (1.14-3.74) | 2.02 (1.14-3.74) | 0.644 | 1 |  |
| Phenylephrine | Increase | 55.6 (42.6-82) | 55.6 (39.7-80.2) | 0.744 | 1 | 59.8 (27.3-63.2) | 58.2 (26.9-64.9) | 1 | 1 | 0.786 (0.602-1.97) | 1 (0.606-1.99) | 0.744 | 1 |  |
| Phenylephrine | Decrease | 69.6 (56.4-81.3) | 61.2 (45.9-84.5) | 0.976 | 1 | 66.4 (63.9-87.7) | 63 (59.3-75.4) | 0.288 | 1 | 0.713 (0.426-0.846) | 0.779 (0.425-1.01) | 0.833 | 1 |  |
| Propofol | Decrease | 66.7 (59.4-73.7) | 67 (59.2-73.6) | 0.802 | 1 | 68.5 (62-76.4) | 69.6 (61.4-78.4) | 0.474 | 1 | 1.46 (0.77-2.44) | 1.56 (0.839-2.53) | 0.394 | 1 |  |
| Propofol | Increase | 66 (59.2-72.4) | 65.3 (58.1-72.5) | 0.502 | 1 | 70.6 (62.3-78.9) | 68.7 (61-76.7) | 0.0984 | 1 | 1.76 (0.993-2.64) | 1.74 (0.905-2.67) | 0.7 | 1 |  |
| Vasopressin | Decrease | 62.8 (56.6-67.2) | 63.8 (52.9-69.2) | 0.966 | 1 | 68.9 (62.4-76.5) | 66.5 (61.9-72.3) | 0.34 | 1 | 1.24 (0.757-2) | 1.26 (0.782-2.13) | 0.601 | 1 |  |
| Vasopressin | Increase | 65.7 (58.1-71.1) | 65.4 (58.1-70.9) | 0.952 | 1 | 63.1 (57.9-68) | 62.7 (56.9-69.7) | 0.904 | 1 | 1.65 (0.99-3.1) | 1.38 (0.715-3.29) | 0.669 | 1 |  |
| **Name** | **Mean Dose Change** | **Mean PRx** | | | | **Mean PAx** | | | | **Mean RAC** | | | |  |
|  |  | **Pre Dose** | **Post Dose** | **P value** | **Adj P Value** | **Pre Dose** | **Post Dose** | **P value** | **Adj P Value** | **Pre Dose** | **Post Dose** | **P value** | **Adj P Value** |  |
| Fentanyl | Decrease | 0.0777 (-0.0602-0.296) | 0.089 (-0.07-0.315) | 0.949 | 1 | -0.0129 (-0.216-0.161) | -0.0331 (-0.212-0.116) | 0.456 | 1 | -0.292 (-0.5--0.0483) | -0.277 (-0.482--0.0371) | 0.697 | 1 |  |
| Fentanyl | Increase | 0.0724 (-0.096-0.305) | 0.0432 (-0.0881-0.237) | 0.72 | 1 | -0.0336 (-0.189-0.139) | -0.0476 (-0.2-0.106) | 0.474 | 1 | -0.269 (-0.46-0) | -0.297 (-0.476--0.0549) | 0.488 | 1 |  |
| Ketamine | Increase | 0.129 (-0.061-0.408) | 0.125 (-0.171-0.293) | 0.566 | 1 | 0.0725 (-0.18-0.264) | 0.0695 (-0.236-0.283) | 0.903 | 1 | -0.211 (-0.407-0.0112) | -0.204 (-0.474-0) | 0.604 | 1 |  |
| Ketamine | Decrease | -0.0291 (-0.0996-0.253) | 0.0181 (-0.213-0.18) | 1 | 1 | -0.0477 (-0.084-0.0359) | -0.0773 (-0.21-0) | 0.369 | 1 | -0.279 (-0.384--0.121) | -0.404 (-0.607--0.193) | 0.216 | 1 |  |
| Midazolam | Increase | -0.0153 (-0.112-0.285) | -0.0325 (-0.225-0.222) | 0.114 | 1 | -0.0891 (-0.205-0.146) | -0.166 (-0.268-0.105) | 0.0846 | 1 | -0.36 (-0.486--0.163) | -0.371 (-0.48--0.195) | 0.869 | 1 |  |
| Midazolam | Decrease | 0 (-0.127-0.239) | 0 (-0.164-0.236) | 0.849 | 1 | -0.0637 (-0.252-0.0992) | -0.00232 (-0.186-0.115) | 0.329 | 1 | -0.289 (-0.443--0.102) | -0.243 (-0.444--0.0266) | 0.663 | 1 |  |
| Norepinephrine | Decrease | 0.0713 (-0.103-0.284) | 0.0655 (-0.104-0.28) | 0.823 | 1 | 0 (-0.187-0.192) | -0.00354 (-0.198-0.19) | 0.489 | 1 | -0.247 (-0.477--0.0181) | -0.264 (-0.484--0.00878) | 0.481 | 1 |  |
| Norepinephrine | Increase | 0.0586 (-0.125-0.293) | 0.0323 (-0.131-0.28) | 0.277 | 1 | -0.0234 (-0.209-0.199) | -0.0153 (-0.198-0.2) | 0.495 | 1 | -0.271 (-0.511-0) | -0.267 (-0.501-0) | 0.593 | 1 |  |
| Phenylephrine | Increase | 0.11 (0.00189-0.278) | 0.132 (0-0.283) | 0.706 | 1 | -0.0777 (-0.289-0) | -0.072 (-0.164-0) | 0.967 | 1 | -0.186 (-0.411-0) | -0.141 (-0.227-0) | 0.558 | 1 |  |
| Phenylephrine | Decrease | 0.264 (0.145-0.428) | 0.11 (0.00189-0.301) | 0.0512 | 1 | -0.137 (-0.273--0.0504) | -0.104 (-0.347-0.187) | 0.928 | 1 | -0.163 (-0.319--0.0128) | -0.174 (-0.346-0.174) | 0.928 | 1 |  |
| Propofol | Decrease | 0.125 (-0.0312-0.324) | 0.129 (-0.0334-0.323) | 0.911 | 1 | 0 (-0.168-0.166) | 0 (-0.16-0.176) | 0.84 | 1 | -0.248 (-0.436-0) | -0.236 (-0.431-0) | 0.973 | 1 |  |
| Propofol | Increase | 0.134 (-0.0219-0.321) | 0.165 (-0.0103-0.338) | 0.394 | 1 | -0.00257 (-0.182-0.197) | 0 (-0.166-0.213) | 0.339 | 1 | -0.26 (-0.453--0.0224) | -0.207 (-0.446-0) | 0.201 | 1 |  |
| Vasopressin | Decrease | 0.0597 (-0.111-0.273) | 0.104 (-0.0493-0.277) | 0.756 | 1 | -0.0288 (-0.225-0.137) | -0.00606 (-0.211-0.0881) | 0.711 | 1 | -0.327 (-0.472--0.11) | -0.258 (-0.507--0.107) | 0.978 | 1 |  |
| Vasopressin | Increase | 0.0725 (-0.0424-0.395) | 0.183 (-0.00691-0.357) | 0.52 | 1 | 0 (-0.129-0.163) | -0.0346 (-0.17-0.14) | 0.473 | 1 | -0.241 (-0.428--0.0243) | -0.169 (-0.388--0.0251) | 0.622 | 1 |  |
| **Name** | **Mean Dose Change** | **Mean COx_L_a** | | | | **Mean COx_R_a** | | | | **% time ICP > 20** | | | |  |
|  |  | **Pre Dose** | **Post Dose** | **P value** | **Adj P Value** | **Pre Dose** | **Post Dose** | **P value** | **Adj P Value** | **Pre Dose** | **Post Dose** | **P value** | **Adj P Value** |  |
| Fentanyl | Decrease | 0.0648 (-0.0267-0.19) | 0.0655 (-0.0184-0.169) | 0.501 | 1 | 0.0882 (0-0.185) | 0.0368 (-0.043-0.211) | 0.229 | 1 | 0 (0-0) | 0 (0-0) | 0.285 | 1 |  |
| Fentanyl | Increase | 0.0707 (0-0.2) | 0.0479 (-0.0268-0.173) | 0.213 | 1 | 0.0952 (0-0.194) | 0.0332 (-0.00972-0.206) | 0.152 | 1 | 0 (0-11.3) | 0 (0-3.23) | 0.241 | 1 |  |
| Ketamine | Increase | 0 (-0.0102-0.131) | 0.0123 (0-0.192) | 0.349 | 1 | 0.00415 (0-0.124) | 0 (0-0.133) | 0.672 | 1 | 0 (0-29) | 0 (0-58.1) | 0.34 | 1 |  |
| Ketamine | Decrease | 0.198 (0-0.338) | 0.0927 (0.00427-0.214) | 0.719 | 1 | 0.0549 (-0.00336-0.342) | 0.0907 (0.0137-0.205) | 0.615 | 1 | 0 (0-0) | 9.68 (0-38.7) | 0.117 | 1 |  |
| Midazolam | Increase | 0.0789 (-0.00301-0.155) | 0.0227 (-0.0366-0.18) | 0.481 | 1 | 0.0737 (-0.0234-0.2) | 0.017 (-0.0603-0.135) | 0.0773 | 1 | 0 (0-4.84) | 0 (0-0) | 0.401 | 1 |  |
| Midazolam | Decrease | 0 (0-0.17) | 0.0101 (0-0.118) | 0.973 | 1 | 0.031 (-0.0579-0.104) | 0.0215 (-0.0616-0.15) | 0.508 | 1 | 0 (0-19.4) | 0 (0-6.45) | 0.759 | 1 |  |
| Norepinephrine | Decrease | 0.0471 (-0.0105-0.163) | 0.0434 (-0.0103-0.162) | 0.854 | 1 | 0.0522 (-0.0187-0.17) | 0.0492 (-0.021-0.166) | 0.587 | 1 | 0 (0-0) | 0 (0-0) | 0.674 | 1 |  |
| Norepinephrine | Increase | 0.0419 (-0.0166-0.162) | 0.0341 (-0.0276-0.165) | 0.375 | 1 | 0.0502 (-0.00905-0.17) | 0.0356 (-0.03-0.164) | 0.125 | 1 | 0 (0-6.45) | 0 (0-3.23) | 0.134 | 1 |  |
| Phenylephrine | Increase | 0 (0-0.0602) | 0 (0-0.206) | 0.732 | 1 | 0 (-0.031-0.0779) | 0 (0-0.0297) | 0.899 | 1 | 0 (0-9.68) | 0 (0-25.8) | 0.838 | 1 |  |
| Phenylephrine | Decrease | 0.115 (-0.0628-0.254) | 0.0991 (-0.0344-0.174) | 0.689 | 1 | 0.154 (-0.0274-0.337) | 0.0468 (-0.0347-0.221) | 0.833 | 1 | 0 (0-0) | 0 (0-0) | 1 | 1 |  |
| Propofol | Decrease | 0.0218 (-0.051-0.17) | 0.036 (-0.0466-0.175) | 0.575 | 1 | 0.0257 (-0.0494-0.158) | 0.0406 (-0.0388-0.176) | 0.553 | 1 | 0 (0-0) | 0 (0-0) | 0.181 | 1 |  |
| Propofol | Increase | 0.0489 (-0.00567-0.177) | 0.0337 (-0.0263-0.171) | 0.34 | 1 | 0.0428 (-0.0373-0.164) | 0.0171 (-0.0659-0.17) | 0.389 | 1 | 0 (0-0) | 0 (0-0) | 0.211 | 1 |  |
| Vasopressin | Decrease | 0.0206 (0-0.174) | 0 (-0.0267-0.087) | 0.185 | 1 | 0.0526 (-0.0638-0.182) | 0.0871 (-0.0897-0.197) | 0.825 | 1 | 0 (0-0) | 0 (0-0) | 0.129 | 1 |  |
| Vasopressin | Increase | 0.00128 (0-0.0873) | 0.0425 (0-0.134) | 0.38 | 1 | 0.035 (-0.0243-0.143) | 0.032 (-0.0156-0.137) | 0.8 | 1 | 0 (0-25.8) | 0 (0-0) | 0.104 | 1 |  |
| **Name** | **Mean Dose Change** | **% time ICP > 22** | | | | **% time CPP > 60** | | | | **% time CPP > 70** | | | |  |
|  |  | **Pre Dose** | **Post Dose** | **P value** | **Adj P Value** | **Pre Dose** | **Post Dose** | **P value** | **Adj P Value** | **Pre Dose** | **Post Dose** | **P value** | **Adj P Value** |  |
| Fentanyl | Decrease | 0 (0-0) | 0 (0-0) | 0.2 | 1 | 3.23 (0-12.9) | 6.45 (0-12.9) | 0.782 | 1 | 41.9 (3.23-87.1) | 54.8 (6.45-87.1) | 0.181 | 1 |  |
| Fentanyl | Increase | 0 (0-3.23) | 0 (0-0) | 0.459 | 1 | 9.68 (0-12.9) | 9.68 (0-12.9) | 0.584 | 1 | 45.2 (6.45-87.1) | 41.9 (3.23-87.1) | 0.902 | 1 |  |
| Ketamine | Increase | 0 (0-6.45) | 0 (0-3.23) | 0.894 | 1 | 12.9 (0-45.2) | 12.9 (0-12.9) | 0.364 | 1 | 83.9 (0-90.3) | 61.3 (0-87.1) | 0.529 | 1 |  |
| Ketamine | Decrease | 0 (0-0) | 0 (0-12.9) | 0.0454 | 1 | 4.84 (0-12.9) | 3.23 (0-19.4) | 0.547 | 1 | 69.4 (15.3-94.4) | 54.8 (0-58.1) | 0.329 | 1 |  |
| Midazolam | Increase | 0 (0-1.61) | 0 (0-0) | 0.265 | 1 | 12.9 (1.61-17.7) | 12.9 (0-12.9) | 0.155 | 1 | 29 (0-87.1) | 74.2 (6.45-88.7) | 0.184 | 1 |  |
| Midazolam | Decrease | 0 (0-3.23) | 0 (0-0) | 0.285 | 1 | 12.9 (0-12.9) | 12.9 (0-12.9) | 0.884 | 1 | 80.6 (0-96.8) | 80.6 (9.68-93.5) | 0.918 | 1 |  |
| Norepinephrine | Decrease | 0 (0-0) | 0 (0-0) | 0.583 | 1 | 9.68 (0-12.9) | 9.68 (0-12.9) | 0.091 | 1 | 64.5 (9.68-87.1) | 51.6 (6.45-87.1) | 0.000281 | 0.00731 |  |
| Norepinephrine | Increase | 0 (0-0) | 0 (0-0) | 0.249 | 1 | 12.9 (0-19.4) | 12.9 (0-12.9) | 0.00117 | 0.0303 | 29 (0-74.2) | 35.5 (0-83.9) | 0.0131 | 0.34 |  |
| Phenylephrine | Increase | 0 (0-0) | 0 (0-25.8) | 0.737 | 1 | 12.9 (12.9-38.7) | 12.9 (12.9-48.4) | 0.709 | 1 | 0 (0-27.4) | 0 (0-40.3) | 0.574 | 1 |  |
| Phenylephrine | Decrease | 0 (0-0) | 0 (0-0) | 1 | 1 | 9.68 (0-21.8) | 12.9 (8.06-30.6) | 0.397 | 1 | 54.8 (4.84-87.1) | 0 (0-87.1) | 0.35 | 1 |  |
| Propofol | Decrease | 0 (0-0) | 0 (0-0) | 0.323 | 1 | 9.68 (0-12.9) | 9.68 (0-12.9) | 0.704 | 1 | 54.8 (3.23-87.1) | 64.5 (6.45-87.1) | 0.177 | 1 |  |
| Propofol | Increase | 0 (0-0) | 0 (0-0) | 0.531 | 1 | 9.68 (0-12.9) | 12.9 (0-12.9) | 0.359 | 1 | 67.7 (6.45-87.1) | 58.1 (6.45-87.1) | 0.203 | 1 |  |
| Vasopressin | Decrease | 0 (0-0) | 0 (0-0) | 0.134 | 1 | 9.68 (0-12.9) | 12.9 (0-12.9) | 0.0956 | 1 | 74.2 (12.9-87.1) | 61.3 (9.68-87.1) | 0.334 | 1 |  |
| Vasopressin | Increase | 0 (0-0) | 0 (0-0) | 0.135 | 1 | 12.9 (8.06-16.9) | 12.9 (0-12.9) | 0.387 | 1 | 17.7 (0-81.5) | 11.3 (0-87.1) | 0.777 | 1 |  |
| **Name** | **Mean Dose Change** | **% time PRx > 0** | | | | **% time PRx > 0.25** | | | | **% time PRx > 0.35** | | | |  |
|  |  | **Pre Dose** | **Post Dose** | **P value** | **Adj P Value** | **Pre Dose** | **Post Dose** | **P value** | **Adj P Value** | **Pre Dose** | **Post Dose** | **P value** | **Adj P Value** |  |
| Fentanyl | Decrease | 56.5 (29-80.6) | 54.8 (32.3-77.4) | 0.972 | 1 | 25.8 (6.45-54.8) | 29 (8.87-58.1) | 0.731 | 1 | 19.4 (0-41.9) | 19.4 (3.23-41.9) | 0.775 | 1 |  |
| Fentanyl | Increase | 51.6 (25.8-74.2) | 48.4 (25.8-79) | 0.996 | 1 | 27.4 (6.45-58.1) | 25.8 (6.45-45.2) | 0.831 | 1 | 16.1 (3.23-43.5) | 16.1 (0-38.7) | 0.62 | 1 |  |
| Ketamine | Increase | 66.1 (28.2-87.1) | 61.3 (22.6-83.9) | 0.674 | 1 | 37.1 (9.68-66.9) | 32.3 (0-61.3) | 0.502 | 1 | 16.1 (2.42-61.3) | 19.4 (0-54.8) | 0.599 | 1 |  |
| Ketamine | Decrease | 30.6 (19.4-75) | 54.8 (22.6-74.2) | 0.981 | 1 | 8.06 (3.23-45.2) | 25.8 (12.9-32.3) | 0.353 | 1 | 3.23 (0.806-31.5) | 19.4 (9.68-25.8) | 0.365 | 1 |  |
| Midazolam | Increase | 48.4 (27.4-74.2) | 38.7 (11.3-75.8) | 0.204 | 1 | 16.1 (3.23-54.8) | 12.9 (0-46.8) | 0.287 | 1 | 6.45 (0-43.5) | 6.45 (0-32.3) | 0.715 | 1 |  |
| Midazolam | Decrease | 41.9 (12.9-74.2) | 45.2 (12.9-67.7) | 0.829 | 1 | 12.9 (0-51.6) | 16.1 (0-45.2) | 0.909 | 1 | 6.45 (0-38.7) | 9.68 (0-32.3) | 0.596 | 1 |  |
| Norepinephrine | Decrease | 54.8 (29-80.6) | 54.8 (25.8-80.6) | 0.63 | 1 | 25.8 (6.45-54.8) | 25.8 (6.45-54.8) | 0.916 | 1 | 16.1 (0-41.9) | 16.1 (0-41.9) | 0.899 | 1 |  |
| Norepinephrine | Increase | 51.6 (25.8-80.6) | 51.6 (22.6-77.4) | 0.487 | 1 | 25.8 (3.23-58.1) | 22.6 (3.23-54.8) | 0.0847 | 1 | 16.1 (0-45.2) | 12.9 (0-41.9) | 0.0362 | 0.94 |  |
| Phenylephrine | Increase | 67.7 (21-87.1) | 64.5 (9.68-82.3) | 0.753 | 1 | 25.8 (4.84-54.8) | 35.5 (1.61-64.5) | 0.769 | 1 | 9.68 (1.61-33.9) | 25.8 (0-43.5) | 0.642 | 1 |  |
| Phenylephrine | Decrease | 71 (61.3-90.3) | 64.5 (45.2-77.4) | 0.165 | 1 | 51.6 (31.5-65.3) | 22.6 (14.5-62.9) | 0.09 | 1 | 37.1 (21.8-47.6) | 9.68 (4.84-56.5) | 0.206 | 1 |  |
| Propofol | Decrease | 64.5 (35.5-83.9) | 64.5 (35.5-87.1) | 0.828 | 1 | 32.3 (9.68-58.1) | 32.3 (6.45-61.3) | 0.841 | 1 | 22.6 (0-48.4) | 22.6 (3.23-48.4) | 0.698 | 1 |  |
| Propofol | Increase | 61.3 (35.5-83.9) | 67.7 (38.7-83.9) | 0.309 | 1 | 32.3 (6.45-61.3) | 35.5 (12.9-64.5) | 0.39 | 1 | 22.6 (3.23-48.4) | 25.8 (3.23-54.8) | 0.276 | 1 |  |
| Vasopressin | Decrease | 61.3 (27.4-83.9) | 53.2 (32.3-80.6) | 0.941 | 1 | 25.8 (3.23-61.3) | 24.2 (3.23-53.2) | 0.95 | 1 | 16.1 (0-45.2) | 14.5 (0-35.5) | 0.877 | 1 |  |
| Vasopressin | Increase | 53.2 (34.7-78.2) | 71 (41.1-87.1) | 0.217 | 1 | 29 (6.45-62.1) | 40.3 (8.06-61.3) | 0.595 | 1 | 14.5 (0-57.3) | 27.4 (2.42-50) | 0.622 | 1 |  |
| **Name** | **Mean Dose Change** | **% time PAx > 0** | | | | **% time PAx > 0.25** | | | | **% time RAC > -0.1** | | | |  |
|  |  | **Pre Dose** | **Post Dose** | **P value** | **Adj P Value** | **Pre Dose** | **Post Dose** | **P value** | **Adj P Value** | **Pre Dose** | **Post Dose** | **P value** | **Adj P Value** |  |
| Fentanyl | Decrease | 41.9 (16.1-67.7) | 35.5 (16.1-64.5) | 0.598 | 1 | 12.9 (0-35.5) | 12.9 (0-32.3) | 0.372 | 1 | 22.6 (12.9-54.8) | 22.6 (12.1-54.8) | 0.71 | 1 |  |
| Fentanyl | Increase | 35.5 (19.4-71) | 35.5 (17.7-62.9) | 0.527 | 1 | 12.9 (0-32.3) | 9.68 (0-33.9) | 0.413 | 1 | 22.6 (12.9-52.4) | 25.8 (12.9-48.4) | 0.873 | 1 |  |
| Ketamine | Increase | 62.9 (9.68-75.8) | 58.1 (12.9-80.6) | 0.996 | 1 | 29 (3.23-58.1) | 22.6 (0-58.1) | 0.759 | 1 | 33.9 (12.9-58.1) | 29 (12.9-58.1) | 0.633 | 1 |  |
| Ketamine | Decrease | 38.7 (23.4-54.8) | 32.3 (19.4-38.7) | 0.355 | 1 | 8.06 (3.23-19.4) | 12.9 (3.23-22.6) | 1 | 1 | 25.8 (9.68-37.9) | 12.9 (3.23-16.1) | 0.342 | 1 |  |
| Midazolam | Increase | 32.3 (16.1-64.5) | 22.6 (6.45-59.7) | 0.193 | 1 | 12.9 (0-38.7) | 6.45 (0-29) | 0.22 | 1 | 12.9 (12.9-45.2) | 16.1 (12.9-45.2) | 0.717 | 1 |  |
| Midazolam | Decrease | 29 (6.45-64.5) | 45.2 (12.9-64.5) | 0.236 | 1 | 9.68 (0-32.3) | 16.1 (0-29) | 0.648 | 1 | 16.1 (12.9-48.4) | 25.8 (12.9-54.8) | 0.26 | 1 |  |
| Norepinephrine | Decrease | 45.2 (19.4-71) | 45.2 (16.1-71) | 0.563 | 1 | 16.1 (0-41.9) | 16.1 (0-41.9) | 0.486 | 1 | 25.8 (12.9-58.1) | 25.8 (12.9-58.1) | 0.259 | 1 |  |
| Norepinephrine | Increase | 41.9 (14.5-71) | 41.9 (16.1-71) | 0.767 | 1 | 12.9 (0-45.2) | 16.1 (0-45.2) | 0.779 | 1 | 25.8 (12.9-58.1) | 25.8 (12.9-58.1) | 0.857 | 1 |  |
| Phenylephrine | Increase | 12.9 (0-37.1) | 25.8 (6.45-45.2) | 0.395 | 1 | 0 (0-1.61) | 0 (0-6.45) | 0.486 | 1 | 12.9 (9.68-40.3) | 29 (12.9-50) | 0.147 | 1 |  |
| Phenylephrine | Decrease | 32.3 (11.3-49.2) | 12.9 (0-59.7) | 0.577 | 1 | 11.3 (0-19.4) | 0 (0-32.3) | 0.445 | 1 | 35.5 (17.7-71) | 25.8 (6.45-61.3) | 0.478 | 1 |  |
| Propofol | Decrease | 41.9 (19.4-71) | 45.2 (19.4-74.2) | 0.577 | 1 | 16.1 (3.23-35.5) | 16.1 (3.23-38.7) | 0.926 | 1 | 25.8 (12.9-58.1) | 25.8 (12.9-58.1) | 0.751 | 1 |  |
| Propofol | Increase | 45.2 (16.1-67.7) | 41.9 (19.4-74.2) | 0.429 | 1 | 12.9 (0-41.9) | 16.1 (0-48.4) | 0.533 | 1 | 25.8 (9.68-54.8) | 29 (12.9-61.3) | 0.131 | 1 |  |
| Vasopressin | Decrease | 41.9 (11.3-69.4) | 40.3 (16.9-62.9) | 0.704 | 1 | 6.45 (0-32.3) | 6.45 (0-30.6) | 0.703 | 1 | 16.1 (6.45-43.5) | 22.6 (12.9-46.8) | 0.458 | 1 |  |
| Vasopressin | Increase | 43.5 (14.5-72.6) | 32.3 (16.1-67.7) | 0.504 | 1 | 11.3 (0-39.5) | 9.68 (0-32.3) | 0.289 | 1 | 21 (12.9-45.2) | 22.6 (12.9-52.4) | 0.785 | 1 |  |
| **Name** | **Mean Dose Change** | **% time RAC > -0.05** | | | | **% time COx_R_a > 0** | | | | **% time COx_R_a > 0.3** | | | |  |
|  |  | **Pre Dose** | **Post Dose** | **P value** | **Adj P Value** | **Pre Dose** | **Post Dose** | **P value** | **Adj P Value** | **Pre Dose** | **Post Dose** | **P value** | **Adj P Value** |  |
| Fentanyl | Decrease | 22.6 (9.68-47.6) | 17.7 (9.68-48.4) | 0.424 | 1 | 54.8 (29.8-71) | 48.4 (23.4-71) | 0.587 | 1 | 21 (6.45-35.5) | 16.1 (3.23-35.5) | 0.28 | 1 |  |
| Fentanyl | Increase | 19.4 (9.68-52.4) | 19.4 (11.3-41.9) | 0.678 | 1 | 54.8 (35.5-74.2) | 51.6 (29-74.2) | 0.536 | 1 | 22.6 (6.45-38.7) | 19.4 (3.23-41.9) | 0.272 | 1 |  |
| Ketamine | Increase | 29 (12.9-58.1) | 25.8 (12.9-48.4) | 0.555 | 1 | 48.4 (0-64.5) | 41.9 (0-64.5) | 0.969 | 1 | 16.1 (0-29) | 9.68 (0-25.8) | 0.848 | 1 |  |
| Ketamine | Decrease | 22.6 (6.45-33.9) | 9.68 (0-16.1) | 0.352 | 1 | 58.1 (41.9-80.6) | 59.7 (43.5-73.4) | 0.827 | 1 | 22.6 (4.84-58.1) | 24.2 (13.7-33.9) | 0.792 | 1 |  |
| Midazolam | Increase | 12.9 (12.9-40.3) | 12.9 (12.9-37.1) | 0.958 | 1 | 48.4 (32.3-71) | 48.4 (24.2-67.7) | 0.182 | 1 | 22.6 (6.45-40.3) | 12.9 (1.61-29) | 0.0427 | 1 |  |
| Midazolam | Decrease | 12.9 (12.9-38.7) | 16.1 (12.9-45.2) | 0.201 | 1 | 45.2 (19.4-61.3) | 41.9 (25.8-61.3) | 0.691 | 1 | 9.68 (0-29) | 12.9 (0-32.3) | 0.234 | 1 |  |
| Norepinephrine | Decrease | 22.6 (12.9-51.6) | 19.4 (9.68-51.6) | 0.254 | 1 | 51.6 (29-71) | 51.6 (25.8-67.7) | 0.479 | 1 | 16.1 (3.23-32.3) | 16.1 (3.23-32.3) | 0.519 | 1 |  |
| Norepinephrine | Increase | 19.4 (12.9-54.8) | 19.4 (9.68-53.2) | 0.942 | 1 | 51.6 (25.8-67.7) | 48.4 (22.6-67.7) | 0.277 | 1 | 16.1 (3.23-32.3) | 12.9 (3.23-32.3) | 0.118 | 1 |  |
| Phenylephrine | Increase | 12.9 (8.06-32.3) | 22.6 (12.9-40.3) | 0.119 | 1 | 29 (3.23-40.3) | 16.1 (0-33.9) | 0.31 | 1 | 0 (0-9.68) | 0 (0-9.68) | 0.885 | 1 |  |
| Phenylephrine | Decrease | 30.6 (12.9-63.7) | 22.6 (6.45-59.7) | 0.496 | 1 | 38.7 (19.4-66.1) | 48.4 (29-66.1) | 0.951 | 1 | 30.6 (9.68-53.2) | 19.4 (1.61-43.5) | 0.597 | 1 |  |
| Propofol | Decrease | 22.6 (9.68-51.6) | 22.6 (12.9-51.6) | 0.918 | 1 | 48.4 (25.8-67.7) | 48.4 (29-67.7) | 0.982 | 1 | 16.1 (3.23-32.3) | 16.1 (3.23-35.5) | 0.933 | 1 |  |
| Propofol | Increase | 22.6 (9.68-51.6) | 22.6 (12.9-54.8) | 0.147 | 1 | 48.4 (29-64.5) | 45.2 (25.8-67.7) | 0.57 | 1 | 16.1 (3.23-35.5) | 12.9 (0-32.3) | 0.526 | 1 |  |
| Vasopressin | Decrease | 12.9 (6.45-33.9) | 17.7 (8.06-37.9) | 0.391 | 1 | 58.1 (25.8-74.2) | 53.2 (31.5-71) | 0.686 | 1 | 12.9 (0-36.3) | 16.1 (2.42-35.5) | 0.626 | 1 |  |
| Vasopressin | Increase | 16.1 (12.9-42.7) | 17.7 (9.68-46) | 0.973 | 1 | 46.8 (22.6-66.1) | 51.6 (29-74.2) | 0.525 | 1 | 11.3 (0.806-25.8) | 12.9 (0-25.8) | 0.731 | 1 |  |
| **Name** | **Mean Dose Change** | **% time COx_L_a > 0** | | | | **% time COx_L_a > 0.3** | | | |  | | | |  |
|  |  | **Pre Dose** | **Post Dose** | **P value** | **Adj P Value** | **Pre Dose** | **Post Dose** | **P value** | **Adj P Value** |  |  |  |  |  |
| Fentanyl | Decrease | 51.6 (35.5-74.2) | 54.8 (35.5-67.7) | 0.705 | 1 | 19.4 (6.45-35.5) | 17.7 (5.65-32.3) | 0.637 | 1 |  |  |  |  |  |
| Fentanyl | Increase | 51.6 (35.5-67.7) | 51.6 (25.8-71) | 0.614 | 1 | 19.4 (6.45-35.5) | 19.4 (0.806-37.9) | 0.292 | 1 |  |  |  |  |  |
| Ketamine | Increase | 38.7 (0-58.1) | 45.2 (12.9-64.5) | 0.902 | 1 | 12.9 (0-32.3) | 6.45 (0-32.3) | 0.628 | 1 |  |  |  |  |  |
| Ketamine | Decrease | 67.7 (32.3-74.2) | 51.6 (41.9-71.8) | 0.705 | 1 | 41.9 (12.9-61.3) | 25.8 (16.1-33.1) | 0.415 | 1 |  |  |  |  |  |
| Midazolam | Increase | 46.8 (25.8-71) | 41.9 (20.2-66.9) | 0.652 | 1 | 22.6 (6.45-36.3) | 14.5 (3.23-32.3) | 0.122 | 1 |  |  |  |  |  |
| Midazolam | Decrease | 25.8 (0-61.3) | 41.9 (0-54.8) | 0.939 | 1 | 9.68 (0-32.3) | 12.9 (0-27.4) | 0.687 | 1 |  |  |  |  |  |
| Norepinephrine | Decrease | 48.4 (25.8-67.7) | 48.4 (25.8-67.7) | 0.622 | 1 | 16.1 (0-29) | 16.1 (0-29) | 0.59 | 1 |  |  |  |  |  |
| Norepinephrine | Increase | 48.4 (25.8-67.7) | 48.4 (22.6-67.7) | 0.557 | 1 | 16.1 (0-32.3) | 12.9 (0-32.3) | 0.459 | 1 |  |  |  |  |  |
| Phenylephrine | Increase | 9.68 (0-27.4) | 19.4 (0-61.3) | 0.381 | 1 | 0 (0-6.45) | 0 (0-33.9) | 0.514 | 1 |  |  |  |  |  |
| Phenylephrine | Decrease | 46.8 (31.5-64.5) | 29 (19.4-48.4) | 0.294 | 1 | 32.3 (15.3-37.9) | 16.1 (1.61-25.8) | 0.214 | 1 |  |  |  |  |  |
| Propofol | Decrease | 48.4 (25.8-67.7) | 48.4 (25.8-71) | 0.734 | 1 | 16.1 (3.23-32.3) | 16.1 (0-35.5) | 0.965 | 1 |  |  |  |  |  |
| Propofol | Increase | 51.6 (29-71) | 51.6 (29-67.7) | 0.336 | 1 | 16.1 (3.23-32.3) | 12.9 (0-32.3) | 0.354 | 1 |  |  |  |  |  |
| Vasopressin | Decrease | 45.2 (0-71) | 35.5 (3.23-61.3) | 0.299 | 1 | 12.9 (0-23.4) | 6.45 (0-29) | 0.826 | 1 |  |  |  |  |  |
| Vasopressin | Increase | 45.2 (0-62.1) | 48.4 (0-72.6) | 0.692 | 1 | 9.68 (0-26.6) | 12.9 (0-27.4) | 0.583 | 1 |  |  |  |  |  |
| **Bolus** | | | | | | | | | | | | | | |
| **Name** | **Doses** | **Mean MAP** | | | | **Mean ICP** | | | | **Mean rSO2_R** | | | |  |
|  |  | **Pre Dose** | **Post Dose** | **P value** | **Adj P Value** | **Pre Dose** | **Post Dose** | **P value** | **Adj P Value** | **Pre Dose** | **Post Dose** | **P value** | **Adj P Value** |  |
| Fentanyl | 167 | 81.9 (72.7-90.5) | 79.6 (72.4-89.1) | 0.367 | 1 | 11.8 (6.16-17.4) | 11.4 (6.25-15.6) | 0.652 | 1 | 65.8 (56.7-72.7) | 66.7 (56.9-72.5) | 0.801 | 1 |  |
| Ketamine | 13 | 78.2 (73-89.6) | 79.3 (74.5-95.3) | 0.605 | 1 | 17.7 (8.45-21.2) | 19.2 (9.7-20.8) | 0.978 | 1 | 65.8 (0-70.4) | 65.9 (0-70.2) | 0.905 | 1 |  |
| Midazolam | 43 | 73.7 (69.2-89.1) | 78.9 (72.2-87.9) | 0.528 | 1 | 11.8 (8.75-16.6) | 10.8 (8-15.8) | 0.254 | 1 | 59 (52.5-67.1) | 59.1 (49.7-65.4) | 0.87 | 1 |  |
| Propofol | 5 | 83.7 (66.6-89.6) | 83.7 (72.4-84.7) | 1 | 1 | 11 (10.5-14) | 10.4 (9.2-11) | 0.6 | 1 | 70.6 (62.3-70.6) | 70.6 (62.8-71.9) | 1 | 1 |  |
| **Name** | **Mean rSO2_L** | | | | **Mean CPP** | | | | **Mean AMP** | | | |  |  |
|  | **Pre Dose** | **Post Dose** | **P value** | **Adj P Value** | **Pre Dose** | **Post Dose** | **P value** | **Adj P Value** | **Pre Dose** | **Post Dose** | **P value** | **Adj P Value** |  |  |
| Fentanyl | 67.8 (61.3-73.6) | 67.5 (61.1-73.4) | 0.797 | 1 | 68.9 (62.9-76.5) | 68.4 (61.9-76) | 0.362 | 1 | 1.92 (1.31-3.31) | 1.97 (1.29-3.6) | 0.949 | 1 |  |  |
| Ketamine | 65.2 (0-68.4) | 65 (0-67.5) | 0.719 | 1 | 66.1 (55.6-81.3) | 69.6 (60.1-79.1) | 0.807 | 1 | 1.15 (1.01-1.88) | 1.22 (0.994-1.56) | 0.765 | 1 |  |  |
| Midazolam | 62.3 (58-67.8) | 63.6 (58-67.2) | 0.969 | 1 | 63.7 (58.8-75) | 67.7 (61-75.4) | 0.21 | 1 | 2.54 (1.21-4.97) | 2.01 (1.14-4.35) | 0.598 | 1 |  |  |
| Propofol | 66.1 (64.9-72.8) | 66 (64.9-72.7) | 1 | 1 | 63.3 (60.1-78.6) | 61.5 (60.1-74.3) | 0.917 | 1 | 2.07 (0.491-3.85) | 1.72 (0.452-4.26) | 1 | 1 |  |  |
| **Name** | **Mean PRx** | | | | **Mean PAx** | | | | **Mean RAC** | | | |  |  |
|  | **Pre Dose** | **Post Dose** | **P value** | **Adj P Value** | **Pre Dose** | **Post Dose** | **P value** | **Adj P Value** | **Pre Dose** | **Post Dose** | **P value** | **Adj P Value** |  |  |
| Fentanyl | 0.0321 (-0.125-0.295) | 0.0409 (-0.0921-0.251) | 0.863 | 1 | -0.0581 (-0.199-0.145) | -0.0507 (-0.239-0.11) | 0.821 | 1 | -0.319 (-0.488--0.0845) | -0.311 (-0.516--0.0742) | 0.888 | 1 |  |  |
| Ketamine | 0.119 (-0.0544-0.409) | 0.205 (-0.0572-0.326) | 0.849 | 1 | 0.132 (-0.0431-0.323) | 0.234 (-0.159-0.283) | 0.765 | 1 | 0.0124 (-0.286-0.191) | -0.0547 (-0.368-0.0832) | 0.683 | 1 |  |  |
| Midazolam | -0.0421 (-0.171-0.328) | -0.0865 (-0.229-0.201) | 0.369 | 1 | -0.102 (-0.209-0.188) | -0.166 (-0.282-0.0963) | 0.325 | 1 | -0.36 (-0.555--0.15) | -0.392 (-0.498--0.176) | 0.931 | 1 |  |  |
| Propofol | 0.235 (-0.0794-0.463) | -0.0794 (-0.172-0.0638) | 0.173 | 1 | -0.0265 (-0.0627-0.0792) | -0.108 (-0.126--0.0627) | 0.116 | 1 | -0.0581 (-0.616--0.00202) | -0.141 (-0.483--0.118) | 0.6 | 1 |  |  |
| **Name** | **Mean COx_L_a** | | | | **Mean COx_R_a** | | | | **% time ICP > 20** | | | |  |  |
|  | **Pre Dose** | **Post Dose** | **P value** | **Adj P Value** | **Pre Dose** | **Post Dose** | **P value** | **Adj P Value** | **Pre Dose** | **Post Dose** | **P value** | **Adj P Value** |  |  |
| Fentanyl | 0.0806 (0.00031-0.207) | 0.0505 (-0.037-0.18) | 0.0664 | 1 | 0.0757 (0-0.195) | 0.037 (-0.0182-0.206) | 0.207 | 1 | 0 (0-16.1) | 0 (0-4.84) | 0.151 | 1 |  |  |
| Ketamine | 0 (0-0.0979) | 0.00617 (0-0.112) | 0.764 | 1 | 0.00351 (0-0.125) | 0 (0-0.0934) | 0.549 | 1 | 14.5 (0-87.1) | 22.6 (0-87.1) | 0.932 | 1 |  |  |
| Midazolam | 0.0789 (0-0.152) | 0.0535 (-0.018-0.18) | 0.672 | 1 | 0.0194 (-0.0445-0.176) | 0.017 (-0.0517-0.106) | 0.39 | 1 | 0 (0-8.06) | 0 (0-0) | 0.431 | 1 |  |  |
| Propofol | 0.124 (0.124-0.349) | -0.000913 (-0.0315-0) | 0.0586 | 1 | 0.0172 (-0.00438-0.339) | -0.038 (-0.101--0.00438) | 0.249 | 1 | 0 (0-0) | 0 (0-0) | 1 | 1 |  |  |
| **Name** | **% time ICP > 22** | | | | **% time CPP > 60** | | | | **% time CPP > 70** | | | |  |  |
|  | **Pre Dose** | **Post Dose** | **P value** | **Adj P Value** | **Pre Dose** | **Post Dose** | **P value** | **Adj P Value** | **Pre Dose** | **Post Dose** | **P value** | **Adj P Value** |  |  |
| Fentanyl | 0 (0-3.23) | 0 (0-0) | 0.283 | 1 | 9.68 (0-12.9) | 9.68 (0-12.9) | 0.782 | 1 | 51.6 (12.9-87.1) | 48.4 (4.84-87.1) | 0.632 | 1 |  |  |
| Ketamine | 3.23 (0-87.1) | 0 (0-87.1) | 0.654 | 1 | 8.06 (0-20.2) | 0 (0-12.9) | 0.684 | 1 | 12.9 (0-97.6) | 54.8 (0-77.4) | 0.779 | 1 |  |  |
| Midazolam | 0 (0-4.84) | 0 (0-0) | 0.449 | 1 | 12.9 (3.23-12.9) | 12.9 (3.23-12.9) | 0.486 | 1 | 45.2 (8.06-87.1) | 83.9 (24.2-90.3) | 0.254 | 1 |  |  |
| Propofol | 0 (0-0) | 0 (0-0) | 1 | 1 | 12.9 (12.9-22.6) | 12.9 (12.9-22.6) | 1 | 1 | 80.6 (38.7-87.1) | 51.6 (38.7-87.1) | 0.914 | 1 |  |  |
| **Name** | **% time PRx > 0** | | | | **% time PRx > 0.25** | | | | **% time PRx > 0.35** | | | |  |  |
|  | **Pre Dose** | **Post Dose** | **P value** | **Adj P Value** | **Pre Dose** | **Post Dose** | **P value** | **Adj P Value** | **Pre Dose** | **Post Dose** | **P value** | **Adj P Value** |  |  |
| Fentanyl | 51.6 (25.8-80.6) | 48.4 (29-77.4) | 0.814 | 1 | 22.6 (3.23-54.8) | 25.8 (4.84-48.4) | 0.996 | 1 | 12.9 (0-41.9) | 16.1 (0-40.3) | 0.615 | 1 |  |  |
| Ketamine | 61.3 (31.5-87.1) | 64.5 (41.9-83.9) | 1 | 1 | 33.9 (12.1-79) | 38.7 (9.68-61.3) | 0.743 | 1 | 25.8 (5.65-64.5) | 38.7 (3.23-58.1) | 0.848 | 1 |  |  |
| Midazolam | 35.5 (21-75.8) | 35.5 (9.68-71) | 0.346 | 1 | 6.45 (1.61-54.8) | 16.1 (0-43.5) | 0.851 | 1 | 3.23 (0-46.8) | 6.45 (0-32.3) | 0.777 | 1 |  |  |
| Propofol | 61.3 (29-74.2) | 29 (25.8-51.6) | 0.139 | 1 | 51.6 (3.23-64.5) | 12.9 (3.23-35.5) | 0.401 | 1 | 48.4 (0-61.3) | 9.68 (0-19.4) | 0.389 | 1 |  |  |
| **Name** | **% time PAx > 0** | | | | **% time PAx > 0.25** | | | | **% time RAC > -0.1** | | | |  |  |
|  | **Pre Dose** | **Post Dose** | **P value** | **Adj P Value** | **Pre Dose** | **Post Dose** | **P value** | **Adj P Value** | **Pre Dose** | **Post Dose** | **P value** | **Adj P Value** |  |  |
| Fentanyl | 35.5 (16.1-71) | 35.5 (16.1-64.5) | 0.778 | 1 | 12.9 (0-35.5) | 9.68 (0-35.5) | 0.73 | 1 | 16.1 (9.68-41.9) | 19.4 (12.9-48.4) | 0.458 | 1 |  |  |
| Ketamine | 71 (40.3-87.1) | 71 (22.6-83.9) | 1 | 1 | 38.7 (16.9-64.5) | 45.2 (9.68-58.1) | 0.978 | 1 | 66.1 (25-87.9) | 54.8 (22.6-77.4) | 0.662 | 1 |  |  |
| Midazolam | 32.3 (8.06-66.1) | 25.8 (6.45-58.1) | 0.542 | 1 | 9.68 (0-40.3) | 6.45 (0-32.3) | 0.78 | 1 | 12.9 (12.9-51.6) | 16.1 (12.9-46.8) | 0.587 | 1 |  |  |
| Propofol | 38.7 (35.5-58.1) | 29 (22.6-35.5) | 0.173 | 1 | 9.68 (6.45-9.68) | 3.23 (0-6.45) | 0.338 | 1 | 54.8 (12.9-61.3) | 45.2 (12.9-58.1) | 0.829 | 1 |  |  |
| **Name** | **% time RAC > -0.05** | | | | **% time COx_R_a > 0** | | | | **% time COx_R_a > 0.3** | | | |  |  |
|  | **Pre Dose** | **Post Dose** | **P value** | **Adj P Value** | **Pre Dose** | **Post Dose** | **P value** | **Adj P Value** | **Pre Dose** | **Post Dose** | **P value** | **Adj P Value** |  |  |
| Fentanyl | 16.1 (9.68-35.5) | 16.1 (9.68-41.9) | 0.613 | 1 | 58.1 (35.5-74.2) | 51.6 (29.8-73.4) | 0.46 | 1 | 22.6 (6.45-38.7) | 19.4 (3.23-41.1) | 0.433 | 1 |  |  |
| Ketamine | 62.9 (25-79.8) | 48.4 (16.1-74.2) | 0.604 | 1 | 40.3 (0-64.5) | 29 (0-66.9) | 0.834 | 1 | 14.5 (0-24.2) | 3.23 (0-16.1) | 0.523 | 1 |  |  |
| Midazolam | 12.9 (12.9-43.5) | 12.9 (12.9-45.2) | 0.853 | 1 | 45.2 (32.3-66.1) | 48.4 (25.8-56.5) | 0.394 | 1 | 22.6 (3.23-38.7) | 12.9 (3.23-25.8) | 0.154 | 1 |  |  |
| Propofol | 51.6 (12.9-58.1) | 41.9 (12.9-45.2) | 0.449 | 1 | 54.8 (41.9-74.2) | 32.3 (29-45.2) | 0.249 | 1 | 3.23 (0-54.8) | 9.68 (6.45-12.9) | 1 | 1 |  |  |
| **Name** | **% time COx_L_a > 0** | | | | **% time COx_L_a > 0.3** | | | |  | | | |  |  |
|  | **Pre Dose** | **Post Dose** | **P value** | **Adj P Value** | **Pre Dose** | **Post Dose** | **P value** | **Adj P Value** |  |  |  |  |  |  |
| Fentanyl | 56.5 (38.7-73.4) | 54.8 (29-70.2) | 0.381 | 1 | 19.4 (6.45-38.7) | 17.7 (3.23-37.9) | 0.241 | 1 |  |  |  |  |  |  |
| Ketamine | 37.1 (0-62.1) | 24.2 (0-59.7) | 0.976 | 1 | 9.68 (0-27.4) | 3.23 (0-16.9) | 0.524 | 1 |  |  |  |  |  |  |
| Midazolam | 54.8 (25.8-71) | 43.5 (20.2-67.7) | 0.763 | 1 | 22.6 (6.45-32.3) | 14.5 (4.03-32.3) | 0.325 | 1 |  |  |  |  |  |  |
| Propofol | 64.5 (64.5-71) | 41.9 (32.3-48.4) | 0.292 | 1 | 32.3 (22.6-51.6) | 6.45 (0-16.1) | 0.168 | 1 |  |  |  |  |  |  |

*The table demonstrates the median and interquartile range of the pre/pose dose windows as well as the Wilcox signed ranked test between these windows with p-values adjusted using the Bonferroni analysis. The “change” indicates if the continuous infusion was increase/decrease or was a bolus dose. AMP, intracranial pulse amplitude; COx_R_a, cerebral oximetry index of right side using MAP; COx_L_a, cerebral oximetry index of left side using MAP; CPP, cerebral prefusion pressure; ICP, intracranial pressure; MAP, mean arterial blood pressure; PAx, pulse amplitude index; PRx, pressure reactivity; RAC, correlation between intracranial pulse amplitude and CPP; rSO2_L, regional oxygen saturation on left side; rSO2_R, regional oxygen saturation on right side.*

# Appendix E. Age < 60

The table contains all infusions given with the patient < 60 years, separated into the continuous infusion doses then the bolus doses.

| **Continuous Infusion** | | | | | | | | | | | | | | |
| --- | --- | --- | --- | --- | --- | --- | --- | --- | --- | --- | --- | --- | --- | --- |
| **Name** | **Doses** | **Mean Dose Change** | **Mean MAP** | | | | **Mean ICP** | | | | **Mean rSO2_R** | | | |
|  |  |  | **Pre Dose** | **Post Dose** | **P value** | **Adj P Value** | **Pre Dose** | **Post Dose** | **P value** | **Adj P Value** | **Pre Dose** | **Post Dose** | **P value** | **Adj P Value** |
| Fentanyl | 33 | Increase | 82.5 (72.7-97.3) | 76.9 (69.3-93.4) | 0.277 | 1 | 12.1 (6.24-19) | 11.9 (6.15-19.8) | 0.924 | 1 | 68.6 (63.6-84.9) | 69.4 (63-79) | 0.807 | 1 |
| Fentanyl | 32 | Decrease | 82.5 (73.5-91.9) | 79.8 (75-92.8) | 0.374 | 1 | 9.78 (8.07-14.2) | 12.5 (9.3-16.4) | 0.19 | 1 | 69 (63.2-73.2) | 69.1 (60.2-73.3) | 0.992 | 1 |
| Ketamine | 8 | Increase | 96.6 (85.6-104) | 96.5 (90.8-101) | 0.712 | 1 | 17.7 (14.1-19.6) | 18.2 (13.9-20) | 1 | 1 | 71.8 (68.3-76.5) | 70.8 (68.8-75) | 0.712 | 1 |
| Ketamine | 4 | Decrease | 76.6 (74.5-87.1) | 78.6 (75.5-88) | 0.748 | 1 | 17.3 (14.8-19.9) | 20.4 (18.3-22.6) | 0.301 | 1 | 67.3 (64.3-89.7) | 68.4 (65.4-89.8) | 0.653 | 1 |
| Midazolam | 16 | Decrease | 97 (91.3-105) | 101 (92.3-104) | 0.792 | 1 | 19.3 (16.9-22.4) | 19 (15.3-23.1) | 0.792 | 1 | 68 (66-69.6) | 67.7 (67-69.8) | 0.88 | 1 |
| Midazolam | 12 | Increase | 102 (92.4-105) | 96.6 (94.4-107) | 0.887 | 1 | 19.4 (16.1-22.9) | 20.4 (17.1-22.3) | 0.713 | 1 | 67.8 (65.7-69.6) | 68.2 (65.3-69.9) | 0.977 | 1 |
| Norepinephrine | 321 | Decrease | 78.2 (71.2-86.8) | 77.5 (72.3-85.1) | 0.5 | 1 | 10 (7.09-14.8) | 10.5 (6.64-14.6) | 0.833 | 1 | 62.2 (51.7-68.2) | 61.7 (50.7-67.9) | 0.84 | 1 |
| Norepinephrine | 269 | Increase | 76 (69.5-83) | 75.5 (69.4-84.6) | 0.921 | 1 | 10.4 (6.43-14.7) | 9.97 (5.95-14.1) | 0.493 | 1 | 62.5 (51.7-69.4) | 63 (50.9-69.2) | 0.986 | 1 |
| Propofol | 45 | Decrease | 77.4 (72.8-83.8) | 77.1 (73.2-83.9) | 0.836 | 1 | 9.27 (6.48-13.4) | 9.56 (5.72-12.5) | 0.838 | 1 | 67.2 (61.4-78.3) | 67.2 (59.7-78.6) | 0.812 | 1 |
| Propofol | 31 | Increase | 81.4 (72.3-90.9) | 77.7 (72.8-84.5) | 0.245 | 1 | 10.5 (5.79-16.1) | 10.1 (5.85-16.4) | 0.98 | 1 | 69.8 (65.5-78.8) | 68.5 (66.1-84.4) | 0.945 | 1 |
| Vasopressin | 5 | Increase | 76.7 (72.8-80.9) | 80.5 (74.1-84.3) | 0.699 | 1 | 13.4 (13.1-13.9) | 11.8 (11.2-14.4) | 0.841 | 1 | 80.1 (54.6-88) | 77.6 (73.8-79.8) | 1 | 1 |
| Vasopressin | 5 | Decrease | 73.8 (73.1-91.1) | 69.6 (63.7-89.1) | 0.421 | 1 | 10.7 (9.65-18.3) | 9.66 (8.28-22.3) | 0.69 | 1 | 63.9 (32-65.5) | 65.5 (32.8-66.9) | 0.825 | 1 |
| **Name** | **Mean Dose Change** | **Mean rSO2_L** | | | | **Mean CPP** | | | | **Mean AMP** | | | |  |
|  |  | **Pre Dose** | **Post Dose** | **P value** | **Adj P Value** | **Pre Dose** | **Post Dose** | **P value** | **Adj P Value** | **Pre Dose** | **Post Dose** | **P value** | **Adj P Value** |  |
| Fentanyl | Increase | 69 (64.8-75.7) | 69 (65.3-74.5) | 0.74 | 1 | 69 (62.8-77.7) | 68.2 (61.2-71.6) | 0.138 | 1 | 3.13 (1.49-6.8) | 2.69 (1.33-7.43) | 0.949 | 1 |  |
| Fentanyl | Decrease | 65.5 (63.2-74.7) | 66 (64-74.7) | 0.814 | 1 | 67.9 (62.6-76.2) | 70 (61.7-80.5) | 0.925 | 1 | 2.16 (1.38-5.09) | 2.54 (1.82-5.03) | 0.321 | 1 |  |
| Ketamine | Increase | 65.2 (64.3-66.7) | 66.6 (65.4-67.8) | 0.712 | 1 | 83.3 (74.5-85.5) | 78 (74.8-82.9) | 0.712 | 1 | 4.27 (3.76-4.66) | 5.67 (4.43-5.87) | 0.102 | 1 |  |
| Ketamine | Decrease | 77.1 (68.9-78.4) | 77.4 (70.2-78.4) | 0.598 | 1 | 69.7 (61.8-77.5) | 65.9 (60.3-71.4) | 0.301 | 1 | 4.75 (2.92-6.57) | 6.17 (4.25-8.1) | 0.301 | 1 |  |
| Midazolam | Decrease | 64.3 (62.5-66.5) | 64.8 (63.8-67.6) | 0.346 | 1 | 78.4 (74.4-84.1) | 79.9 (76.6-83.8) | 0.522 | 1 | 5.81 (4.75-7.37) | 5.74 (4.7-7.59) | 0.91 | 1 |  |
| Midazolam | Increase | 64.3 (61.9-67.5) | 64.9 (61.7-67.5) | 0.799 | 1 | 82 (74.5-85.9) | 78.4 (75.7-84.2) | 0.59 | 1 | 5.46 (4.59-7.69) | 6.67 (4.96-8.36) | 0.347 | 1 |  |
| Norepinephrine | Decrease | 64.3 (57.7-68.7) | 64.1 (57.4-68.7) | 0.675 | 1 | 68.8 (61.7-76.5) | 68 (61.5-74.8) | 0.327 | 1 | 2.93 (1.51-4.61) | 2.9 (1.43-4.64) | 0.857 | 1 |  |
| Norepinephrine | Increase | 64.6 (58.3-71.1) | 64.7 (58.1-71.3) | 0.936 | 1 | 66.2 (60.5-71.6) | 66.6 (61.1-72.6) | 0.548 | 1 | 2.82 (1.38-4.42) | 2.95 (1.41-4.44) | 0.983 | 1 |  |
| Propofol | Decrease | 68.4 (62.9-77) | 67.8 (62.3-77.4) | 0.843 | 1 | 68.7 (63.2-73.4) | 67.4 (61.5-79.4) | 0.875 | 1 | 2.51 (1.26-3.89) | 2.54 (1.43-4.1) | 0.966 | 1 |  |
| Propofol | Increase | 67.1 (63.3-76.9) | 66.9 (64-74.8) | 0.757 | 1 | 76.2 (63.8-81.3) | 68.7 (59.8-75.2) | 0.0532 | 1 | 2.59 (1.63-5.79) | 3.22 (1.53-4.76) | 0.973 | 1 |  |
| Vasopressin | Increase | 72.1 (66.5-74.1) | 70.8 (69-70.9) | 0.841 | 1 | 65.4 (61.5-67.4) | 65.1 (63.4-73.2) | 0.69 | 1 | 2.47 (1.84-3.49) | 1.99 (1.29-2.95) | 0.841 | 1 |  |
| Vasopressin | Decrease | 66.7 (63.6-69.6) | 69.1 (57.1-71.3) | 1 | 1 | 65.3 (61.9-68.6) | 61.3 (58.3-65.9) | 0.421 | 1 | 3.39 (2.83-5.55) | 2.96 (2.21-7.66) | 1 | 1 |  |
| **Name** | **Mean Dose Change** | **Mean PRx** | | | | **Mean PAx** | | | | **Mean RAC** | | | |  |
|  |  | **Pre Dose** | **Post Dose** | **P value** | **Adj P Value** | **Pre Dose** | **Post Dose** | **P value** | **Adj P Value** | **Pre Dose** | **Post Dose** | **P value** | **Adj P Value** |  |
| Fentanyl | Increase | 0.206 (0.00161-0.389) | 0.19 (0-0.363) | 0.865 | 1 | 0.139 (0.0459-0.328) | 0.107 (-0.0248-0.363) | 0.875 | 1 | -0.0413 (-0.197-0.226) | 0 (-0.209-0.255) | 0.713 | 1 |  |
| Fentanyl | Decrease | 0.127 (0.0158-0.312) | 0.156 (-0.0362-0.386) | 0.904 | 1 | 0.107 (-0.00719-0.366) | 0.0173 (-0.145-0.195) | 0.104 | 1 | -0.0405 (-0.235-0.234) | -0.136 (-0.325-0.0457) | 0.143 | 1 |  |
| Ketamine | Increase | 0.195 (-0.0283-0.418) | 0.193 (-0.00192-0.216) | 0.429 | 1 | 0.213 (0.149-0.256) | 0.243 (0.0934-0.297) | 0.792 | 1 | 0.0607 (-0.123-0.0918) | -0.0147 (-0.162-0.0625) | 0.712 | 1 |  |
| Ketamine | Decrease | -0.228 (-0.378--0.0785) | -0.26 (-0.306--0.213) | 1 | 1 | -0.0959 (-0.24-0.0479) | -0.166 (-0.229--0.103) | 1 | 1 | -0.34 (-0.384--0.296) | -0.505 (-0.607--0.404) | 0.0265 | 0.689 |  |
| Midazolam | Decrease | -0.147 (-0.253--0.0293) | -0.207 (-0.35--0.021) | 0.624 | 1 | -0.163 (-0.246--0.00293) | -0.152 (-0.282-0.08) | 0.97 | 1 | -0.356 (-0.397--0.316) | -0.438 (-0.47--0.222) | 0.474 | 1 |  |
| Midazolam | Increase | -0.0849 (-0.187--0.0396) | -0.169 (-0.342-0.0323) | 0.63 | 1 | -0.0617 (-0.227-0.0842) | -0.148 (-0.287-0.0014) | 0.443 | 1 | -0.338 (-0.384--0.134) | -0.432 (-0.516--0.28) | 0.178 | 1 |  |
| Norepinephrine | Decrease | 0.117 (-0.0882-0.283) | 0.0996 (-0.106-0.285) | 0.56 | 1 | 0.0505 (-0.114-0.237) | 0.0243 (-0.128-0.226) | 0.485 | 1 | -0.123 (-0.305-0.0505) | -0.116 (-0.324-0.057) | 0.966 | 1 |  |
| Norepinephrine | Increase | 0.0975 (-0.135-0.268) | 0.0784 (-0.099-0.258) | 0.987 | 1 | 0.0288 (-0.139-0.228) | 0.0378 (-0.119-0.228) | 0.662 | 1 | -0.127 (-0.345-0.0651) | -0.117 (-0.336-0.0592) | 0.675 | 1 |  |
| Propofol | Decrease | 0.126 (-0.0275-0.328) | 0.162 (-0.0163-0.341) | 0.802 | 1 | 0.0889 (-0.103-0.25) | 0.0428 (-0.0791-0.278) | 0.757 | 1 | -0.0386 (-0.215-0.154) | -0.0352 (-0.245-0.147) | 0.983 | 1 |  |
| Propofol | Increase | 0.172 (0.0313-0.363) | 0.179 (-0.0162-0.423) | 0.862 | 1 | 0.0355 (-0.112-0.217) | 0.197 (-0.052-0.375) | 0.163 | 1 | -0.00202 (-0.212-0.121) | -0.0075 (-0.115-0.189) | 0.621 | 1 |  |
| Vasopressin | Increase | 0.306 (0.142-0.64) | 0.408 (0.396-0.621) | 0.69 | 1 | 0.0399 (-0.106-0.233) | -0.0585 (-0.139-0.084) | 0.548 | 1 | -0.116 (-0.182--0.0249) | -0.128 (-0.262--0.0287) | 0.841 | 1 |  |
| Vasopressin | Decrease | -0.147 (-0.229-0.214) | 0.26 (-0.000332-0.269) | 0.31 | 1 | -0.108 (-0.17-0.197) | 0.0158 (-0.0913-0.085) | 1 | 1 | -0.409 (-0.423--0.198) | -0.123 (-0.227--0.092) | 0.31 | 1 |  |
| **Name** | **Mean Dose Change** | **Mean COx_L_a** | | | | **Mean COx_R_a** | | | | **% time ICP > 20** | | | |  |
|  |  | **Pre Dose** | **Post Dose** | **P value** | **Adj P Value** | **Pre Dose** | **Post Dose** | **P value** | **Adj P Value** | **Pre Dose** | **Post Dose** | **P value** | **Adj P Value** |  |
| Fentanyl | Increase | 0.0582 (0-0.121) | 0.055 (0-0.147) | 0.829 | 1 | 0.179 (0.102-0.285) | 0.178 (0.0116-0.243) | 0.338 | 1 | 0 (0-32.3) | 0 (0-44.4) | 0.717 | 1 |  |
| Fentanyl | Decrease | 0.0924 (0-0.17) | 0.106 (0.0347-0.223) | 0.333 | 1 | 0.129 (0.0428-0.266) | 0.193 (0.0685-0.299) | 0.607 | 1 | 0 (0-0) | 0 (0-24.2) | 0.0761 | 1 |  |
| Ketamine | Increase | 0.138 (0.0583-0.218) | 0.2 (0.14-0.211) | 0.792 | 1 | 0.124 (0.0565-0.178) | 0.274 (0.219-0.377) | 0.0132 | 0.343 | 14.5 (0-29.8) | 14.5 (4.84-37.1) | 0.705 | 1 |  |
| Ketamine | Decrease | 0.198 (0.131-0.502) | 0.09 (0.0151-0.314) | 0.404 | 1 | 0.658 (-0.00336-0.681) | 0.378 (0.0944-0.72) | 0.752 | 1 | 22.6 (0-45.2) | 61.3 (38.7-83.9) | 0.301 | 1 |  |
| Midazolam | Decrease | 0.0816 (-0.00019-0.143) | 0.0684 (0.0359-0.14) | 0.94 | 1 | 0.0622 (0.00333-0.102) | 0.0762 (0.0137-0.142) | 0.451 | 1 | 37.1 (0-94.4) | 14.5 (0-95.2) | 0.892 | 1 |  |
| Midazolam | Increase | 0.0198 (-0.00667-0.094) | 0.0479 (-0.0522-0.206) | 0.671 | 1 | 0.0363 (-0.0414-0.145) | 0.0255 (-0.0187-0.119) | 0.932 | 1 | 41.9 (0-90.3) | 48.4 (8.87-72.6) | 0.816 | 1 |  |
| Norepinephrine | Decrease | 0.0555 (0-0.176) | 0.0688 (0-0.175) | 0.834 | 1 | 0.0944 (0.0118-0.191) | 0.0962 (0.0137-0.211) | 0.515 | 1 | 0 (0-0) | 0 (0-0) | 0.831 | 1 |  |
| Norepinephrine | Increase | 0.0585 (0-0.198) | 0.046 (0-0.18) | 0.816 | 1 | 0.121 (0.0102-0.22) | 0.116 (0-0.196) | 0.352 | 1 | 0 (0-0) | 0 (0-0) | 0.0683 | 1 |  |
| Propofol | Decrease | 0.0704 (-0.0135-0.195) | 0.102 (0.0368-0.216) | 0.126 | 1 | 0.0798 (-0.0235-0.235) | 0.122 (0.0151-0.211) | 0.427 | 1 | 0 (0-0) | 0 (0-0) | 0.681 | 1 |  |
| Propofol | Increase | 0.0877 (0.0258-0.235) | 0.0942 (0-0.287) | 0.969 | 1 | 0.101 (0.027-0.274) | 0.19 (-0.0277-0.359) | 0.931 | 1 | 0 (0-11.3) | 0 (0-6.45) | 0.656 | 1 |  |
| Vasopressin | Increase | 0.053 (0.0358-0.0747) | 0 (-0.0212-0.0589) | 0.623 | 1 | 0.174 (0.124-0.204) | 0.047 (-0.0169-0.0744) | 0.222 | 1 | 0 (0-0) | 0 (0-0) | 0.424 | 1 |  |
| Vasopressin | Decrease | 0.0207 (0.0104-0.0458) | 0.292 (0.146-0.301) | 0.507 | 1 | 0.0587 (0.0264-0.204) | 0.179 (0.145-0.272) | 0.4 | 1 | 0 (0-12.9) | 0 (0-74.2) | 1 | 1 |  |
| **Name** | **Mean Dose Change** | **% time ICP > 22** | | | | **% time CPP > 60** | | | | **% time CPP > 70** | | | |  |
|  |  | **Pre Dose** | **Post Dose** | **P value** | **Adj P Value** | **Pre Dose** | **Post Dose** | **P value** | **Adj P Value** | **Pre Dose** | **Post Dose** | **P value** | **Adj P Value** |  |
| Fentanyl | Increase | 0 (0-0) | 0 (0-32.3) | 0.0869 | 1 | 6.45 (0-19.4) | 9.68 (0-12.9) | 0.664 | 1 | 51.6 (0-87.9) | 12.9 (0-61.3) | 0.354 | 1 |  |
| Fentanyl | Decrease | 0 (0-0) | 0 (0-0) | 0.199 | 1 | 0 (0-12.9) | 4.84 (0-12.9) | 0.436 | 1 | 43.5 (2.42-87.1) | 46.8 (0-94.4) | 0.869 | 1 |  |
| Ketamine | Increase | 3.23 (0-11.3) | 0 (0-0) | 0.0323 | 0.839 | 3.23 (0-21.8) | 0 (0-0.806) | 0.164 | 1 | 95.2 (67.7-100) | 88.7 (66.1-100) | 1 | 1 |  |
| Ketamine | Decrease | 0 (0-0) | 32.3 (0-64.5) | 0.181 | 1 | 21 (0-41.9) | 24.2 (3.23-45.2) | 0.301 | 1 | 51.6 (6.45-96.8) | 27.4 (0-54.8) | 0.301 | 1 |  |
| Midazolam | Decrease | 3.23 (0-60.5) | 0 (0-87.1) | 0.671 | 1 | 0 (0-1.61) | 0 (0-3.23) | 0.757 | 1 | 96.8 (79-100) | 98.4 (79-100) | 0.735 | 1 |  |
| Midazolam | Increase | 8.06 (0-58.9) | 11.3 (0-55.6) | 0.811 | 1 | 0 (0-3.23) | 0 (0-1.61) | 0.857 | 1 | 96.8 (92.7-100) | 98.4 (87.1-100) | 0.855 | 1 |  |
| Norepinephrine | Decrease | 0 (0-0) | 0 (0-0) | 0.738 | 1 | 3.23 (0-12.9) | 6.45 (0-12.9) | 0.0981 | 1 | 56.5 (9.68-93.5) | 46.8 (6.45-87.1) | 0.111 | 1 |  |
| Norepinephrine | Increase | 0 (0-0) | 0 (0-0) | 0.441 | 1 | 9.68 (0-19.4) | 6.45 (0-12.9) | 0.0316 | 0.823 | 29 (0-70.2) | 32.3 (0.806-76.6) | 0.237 | 1 |  |
| Propofol | Decrease | 0 (0-0) | 0 (0-0) | 0.898 | 1 | 0 (0-12.9) | 9.68 (0-12.9) | 0.318 | 1 | 43.5 (0-87.1) | 54.8 (0-90.3) | 0.7 | 1 |  |
| Propofol | Increase | 0 (0-2.42) | 0 (0-5.65) | 0.647 | 1 | 0 (0-11.3) | 3.23 (0-38.7) | 0.0709 | 1 | 80.6 (21-90.3) | 45.2 (0-80.6) | 0.0771 | 1 |  |
| Vasopressin | Increase | 0 (0-0) | 0 (0-0) | 0.424 | 1 | 12.9 (0-19.4) | 0 (0-12.9) | 0.576 | 1 | 3.23 (0-83.9) | 87.1 (0-96.8) | 0.449 | 1 |  |
| Vasopressin | Decrease | 0 (0-0) | 0 (0-58.1) | 0.797 | 1 | 12.9 (0-25.8) | 16.1 (12.9-38.7) | 0.344 | 1 | 9.68 (6.45-48.4) | 22.6 (0-48.4) | 1 | 1 |  |
| **Name** | **Mean Dose Change** | **% time PRx > 0** | | | | **% time PRx > 0.25** | | | | **% time PRx > 0.35** | | | |  |
|  |  | **Pre Dose** | **Post Dose** | **P value** | **Adj P Value** | **Pre Dose** | **Post Dose** | **P value** | **Adj P Value** | **Pre Dose** | **Post Dose** | **P value** | **Adj P Value** |  |
| Fentanyl | Increase | 74.2 (50.8-84.7) | 58.1 (38.7-87.1) | 0.808 | 1 | 38.7 (12.9-71) | 45.2 (25.8-77.4) | 0.524 | 1 | 29 (12.9-64.5) | 29 (19.4-71) | 0.442 | 1 |  |
| Fentanyl | Decrease | 64.5 (45.2-93.5) | 67.7 (37.9-90.3) | 0.638 | 1 | 32.3 (11.3-67.7) | 37.1 (15.3-66.1) | 0.914 | 1 | 27.4 (6.45-41.9) | 25.8 (6.45-58.9) | 0.84 | 1 |  |
| Ketamine | Increase | 72.6 (60.5-80.6) | 56.5 (19.4-81.5) | 0.562 | 1 | 46.8 (25-71) | 32.3 (12.1-44.4) | 0.225 | 1 | 37.1 (12.9-66.9) | 22.6 (12.1-31.5) | 0.426 | 1 |  |
| Ketamine | Decrease | 22.6 (16.1-29) | 19.4 (16.1-22.6) | 0.64 | 1 | 3.23 (3.23-3.23) | 12.9 (6.45-19.4) | 0.0194 | 0.503 | 1.61 (0-3.23) | 11.3 (3.23-19.4) | 0.0864 | 1 |  |
| Midazolam | Decrease | 21 (8.87-45.2) | 29 (8.06-42.7) | 0.88 | 1 | 0 (0-12.9) | 4.84 (0-13.7) | 0.564 | 1 | 0 (0-7.26) | 0 (0-11.3) | 0.917 | 1 |  |
| Midazolam | Increase | 29 (18.5-53.2) | 21 (9.68-47.6) | 0.47 | 1 | 9.68 (3.23-17.7) | 6.45 (0-21.8) | 0.77 | 1 | 4.84 (0-12.9) | 3.23 (0-14.5) | 1 | 1 |  |
| Norepinephrine | Decrease | 61.3 (32.3-83.9) | 61.3 (29-80.6) | 0.338 | 1 | 29 (9.68-54.8) | 29 (6.45-54.8) | 0.565 | 1 | 19.4 (3.23-38.7) | 19.4 (3.23-41.9) | 0.723 | 1 |  |
| Norepinephrine | Increase | 54.8 (25.8-77.4) | 58.1 (25.8-80.6) | 0.443 | 1 | 32.3 (6.45-51.6) | 25.8 (3.23-54.8) | 0.67 | 1 | 19.4 (3.23-38.7) | 12.9 (0-41.9) | 0.337 | 1 |  |
| Propofol | Decrease | 66.1 (35.5-90.3) | 67.7 (41.9-87.1) | 0.868 | 1 | 33.9 (6.45-58.1) | 38.7 (16.1-61.3) | 0.542 | 1 | 19.4 (0-50.8) | 22.6 (6.45-54.8) | 0.438 | 1 |  |
| Propofol | Increase | 61.3 (41.9-79) | 71 (43.5-79) | 0.944 | 1 | 32.3 (21-58.1) | 38.7 (25.8-64.5) | 0.653 | 1 | 22.6 (9.68-51.6) | 32.3 (12.9-61.3) | 0.511 | 1 |  |
| Vasopressin | Increase | 96.8 (54.8-100) | 100 (87.1-100) | 0.504 | 1 | 48.4 (35.5-96.8) | 90.3 (83.9-100) | 0.398 | 1 | 32.3 (25.8-90.3) | 74.2 (54.8-100) | 0.398 | 1 |  |
| Vasopressin | Decrease | 22.6 (12.9-83.9) | 83.9 (48.4-93.5) | 0.173 | 1 | 16.1 (12.9-38.7) | 45.2 (45.2-58.1) | 0.295 | 1 | 12.9 (12.9-29) | 35.5 (32.3-35.5) | 0.343 | 1 |  |
| **Name** | **Mean Dose Change** | **% time PAx > 0** | | | | **% time PAx > 0.25** | | | | **% time RAC > -0.1** | | | |  |
|  |  | **Pre Dose** | **Post Dose** | **P value** | **Adj P Value** | **Pre Dose** | **Post Dose** | **P value** | **Adj P Value** | **Pre Dose** | **Post Dose** | **P value** | **Adj P Value** |  |
| Fentanyl | Increase | 66.1 (51.6-83.9) | 64.5 (45.2-90.3) | 0.603 | 1 | 30.6 (12.9-57.3) | 35.5 (9.68-61.3) | 0.585 | 1 | 59.7 (31.5-77.4) | 54.8 (32.3-90.3) | 0.545 | 1 |  |
| Fentanyl | Decrease | 64.5 (49.2-90.3) | 51.6 (28.2-74.2) | 0.0634 | 1 | 35.5 (9.68-61.3) | 24.2 (5.65-35.5) | 0.0911 | 1 | 59.7 (25.8-91.1) | 40.3 (19.4-71) | 0.129 | 1 |  |
| Ketamine | Increase | 71 (70.2-75) | 71 (41.9-83.1) | 0.71 | 1 | 50 (38.7-58.1) | 29 (19.4-38.7) | 0.0759 | 1 | 64.5 (43.5-76.6) | 54.8 (38.7-58.9) | 0.29 | 1 |  |
| Ketamine | Decrease | 40.3 (22.6-58.1) | 29 (19.4-38.7) | 0.301 | 1 | 11.3 (3.23-19.4) | 11.3 (9.68-12.9) | 1 | 1 | 17.7 (9.68-25.8) | 9.68 (3.23-16.1) | 0.301 | 1 |  |
| Midazolam | Decrease | 29 (17.7-54.8) | 37.1 (11.3-62.9) | 0.91 | 1 | 4.84 (0-13.7) | 11.3 (0-24.2) | 0.645 | 1 | 9.68 (2.42-19.4) | 12.9 (3.23-25.8) | 0.608 | 1 |  |
| Midazolam | Increase | 48.4 (12.9-60.5) | 30.6 (17.7-51.6) | 0.685 | 1 | 9.68 (2.42-36.3) | 8.06 (0-32.3) | 0.815 | 1 | 21 (5.65-55.6) | 14.5 (12.1-23.4) | 0.908 | 1 |  |
| Norepinephrine | Decrease | 54.8 (29-77.4) | 51.6 (29-77.4) | 0.279 | 1 | 22.6 (3.23-48.4) | 19.4 (3.23-45.2) | 0.473 | 1 | 48.4 (19.4-74.2) | 48.4 (16.1-77.4) | 0.646 | 1 |  |
| Norepinephrine | Increase | 51.6 (25.8-76.6) | 54.8 (25.8-77.4) | 0.579 | 1 | 19.4 (3.23-48.4) | 22.6 (3.23-45.2) | 0.706 | 1 | 45.2 (16.1-71) | 45.2 (19.4-74.2) | 0.389 | 1 |  |
| Propofol | Decrease | 62.9 (29-86.3) | 54.8 (29-80.6) | 0.462 | 1 | 25.8 (3.23-51.6) | 29 (3.23-51.6) | 0.841 | 1 | 62.9 (22.6-83.1) | 58.1 (29-80.6) | 0.848 | 1 |  |
| Propofol | Increase | 51.6 (30.6-77.4) | 64.5 (38.7-88.7) | 0.148 | 1 | 32.3 (9.68-53.2) | 41.9 (12.9-64.5) | 0.271 | 1 | 54.8 (32.3-71) | 58.1 (43.5-87.1) | 0.252 | 1 |  |
| Vasopressin | Increase | 58.1 (32.3-77.4) | 35.5 (19.4-54.8) | 0.548 | 1 | 6.45 (6.45-51.6) | 25.8 (0-32.3) | 0.832 | 1 | 41.9 (35.5-64.5) | 41.9 (22.6-48.4) | 0.917 | 1 |  |
| Vasopressin | Decrease | 41.9 (22.6-83.9) | 54.8 (32.3-58.1) | 1 | 1 | 6.45 (0-32.3) | 22.6 (0-32.3) | 1 | 1 | 9.68 (6.45-25.8) | 54.8 (51.6-58.1) | 0.209 | 1 |  |
| **Name** | **Mean Dose Change** | **% time RAC > -0.05** | | | | **% time COx_R_a > 0** | | | | **% time COx_R_a > 0.3** | | | |  |
|  |  | **Pre Dose** | **Post Dose** | **P value** | **Adj P Value** | **Pre Dose** | **Post Dose** | **P value** | **Adj P Value** | **Pre Dose** | **Post Dose** | **P value** | **Adj P Value** |  |
| Fentanyl | Increase | 59.7 (30.6-71) | 51.6 (22.6-90.3) | 0.664 | 1 | 72.6 (55.6-80.6) | 69.4 (44.4-78.2) | 0.513 | 1 | 30.6 (15.3-48.4) | 29 (9.68-46.8) | 0.493 | 1 |  |
| Fentanyl | Decrease | 48.4 (25.8-80.6) | 37.1 (16.1-66.1) | 0.134 | 1 | 58.1 (35.5-71) | 71 (48.4-83.9) | 0.361 | 1 | 29 (9.68-38.7) | 45.2 (12.9-51.6) | 0.378 | 1 |  |
| Ketamine | Increase | 61.3 (43.5-69.4) | 41.9 (29-55.6) | 0.139 | 1 | 56.5 (44.4-66.1) | 80.6 (68.5-87.9) | 0.0232 | 0.604 | 30.6 (21.8-40.3) | 41.9 (28.2-49.2) | 0.428 | 1 |  |
| Ketamine | Decrease | 14.5 (6.45-22.6) | 9.68 (3.23-16.1) | 0.301 | 1 | 83.9 (51.6-90.3) | 77.4 (61.3-90.3) | 0.833 | 1 | 77.4 (19.4-90.3) | 64.5 (29-90.3) | 0.833 | 1 |  |
| Midazolam | Decrease | 6.45 (0-16.1) | 9.68 (3.23-16.1) | 0.444 | 1 | 53.2 (39.5-55.6) | 51.6 (25-61.3) | 0.97 | 1 | 8.06 (3.23-15.3) | 12.9 (4.84-32.3) | 0.304 | 1 |  |
| Midazolam | Increase | 17.7 (3.23-37.1) | 11.3 (4.84-21.8) | 0.793 | 1 | 38.7 (31.5-65.3) | 53.2 (21.8-63.7) | 0.686 | 1 | 25.8 (12.1-33.1) | 9.68 (2.42-23.4) | 0.485 | 1 |  |
| Norepinephrine | Decrease | 41.9 (16.1-67.7) | 41.9 (12.9-67.7) | 0.628 | 1 | 54.8 (38.7-74.2) | 54.8 (38.7-71) | 0.992 | 1 | 19.4 (8.87-35.5) | 22.6 (7.26-35.5) | 0.745 | 1 |  |
| Norepinephrine | Increase | 38.7 (12.9-64.5) | 41.9 (12.9-67.7) | 0.387 | 1 | 58.1 (38.7-74.2) | 54.8 (38.7-74.2) | 0.611 | 1 | 22.6 (9.68-41.9) | 22.6 (9.68-37.1) | 0.447 | 1 |  |
| Propofol | Decrease | 51.6 (19.4-77.4) | 45.2 (22.6-77.4) | 0.845 | 1 | 53.2 (32.3-77.4) | 59.7 (44.4-75) | 0.426 | 1 | 17.7 (6.45-45.2) | 21 (5.65-46) | 0.719 | 1 |  |
| Propofol | Increase | 51.6 (24.2-69.4) | 51.6 (38.7-80.6) | 0.341 | 1 | 59.7 (38.7-77.4) | 71 (35.5-83.9) | 0.795 | 1 | 22.6 (9.68-46) | 45.2 (6.45-54.8) | 0.856 | 1 |  |
| Vasopressin | Increase | 32.3 (25.8-54.8) | 22.6 (12.9-41.9) | 0.69 | 1 | 67.7 (67.7-74.2) | 48.4 (38.7-58.1) | 0.248 | 1 | 25.8 (25.8-32.3) | 3.23 (0-19.4) | 0.207 | 1 |  |
| Vasopressin | Decrease | 9.68 (0-19.4) | 38.7 (35.5-41.9) | 0.141 | 1 | 71 (58.1-72.6) | 41.9 (37.1-62.9) | 0.7 | 1 | 9.68 (9.68-40.3) | 29 (17.7-40.3) | 1 | 1 |  |
| **Name** | **Mean Dose Change** | **% time COx_L_a > 0** | | | | **% time COx_L_a > 0.3** | | | |  | | | |  |
|  |  | **Pre Dose** | **Post Dose** | **P value** | **Adj P Value** | **Pre Dose** | **Post Dose** | **P value** | **Adj P Value** |  |  |  |  |  |
| Fentanyl | Increase | 48.4 (22.6-62.9) | 51.6 (25.8-61.3) | 0.891 | 1 | 12.9 (0-29) | 17.7 (0.806-35.5) | 0.696 | 1 |  |  |  |  |  |
| Fentanyl | Decrease | 48.4 (30.6-75.8) | 53.2 (45.2-74.2) | 0.53 | 1 | 12.9 (3.23-27.4) | 25.8 (12.9-37.9) | 0.147 | 1 |  |  |  |  |  |
| Ketamine | Increase | 62.9 (47.6-78.2) | 40.3 (15.3-59.7) | 0.102 | 1 | 37.1 (26.6-43.5) | 25.8 (12.1-36.3) | 0.224 | 1 |  |  |  |  |  |
| Ketamine | Decrease | 67.7 (56.5-79) | 48.4 (43.5-66.1) | 0.334 | 1 | 45.2 (33.9-72.6) | 22.6 (16.1-50) | 0.172 | 1 |  |  |  |  |  |
| Midazolam | Decrease | 40.3 (25.8-56.5) | 45.2 (40.3-52.4) | 0.762 | 1 | 12.9 (8.87-19.4) | 21 (9.68-29) | 0.416 | 1 |  |  |  |  |  |
| Midazolam | Increase | 27.4 (25.8-46) | 50 (16.1-65.3) | 0.369 | 1 | 16.1 (6.45-20.2) | 17.7 (8.87-36.3) | 0.399 | 1 |  |  |  |  |  |
| Norepinephrine | Decrease | 45.2 (16.1-67.7) | 45.2 (16.1-64.5) | 0.611 | 1 | 12.9 (0-32.3) | 12.9 (0-32.3) | 0.896 | 1 |  |  |  |  |  |
| Norepinephrine | Increase | 41.9 (9.68-67.7) | 41.9 (10.5-71) | 0.724 | 1 | 16.1 (0-35.5) | 12.9 (0-32.3) | 0.753 | 1 |  |  |  |  |  |
| Propofol | Decrease | 51.6 (29-72.6) | 61.3 (31.5-73.4) | 0.45 | 1 | 9.68 (4.84-30.6) | 19.4 (9.68-37.9) | 0.113 | 1 |  |  |  |  |  |
| Propofol | Increase | 58.1 (42.7-72.6) | 58.1 (35.5-71.8) | 0.773 | 1 | 22.6 (12.9-41.1) | 27.4 (5.65-46) | 0.938 | 1 |  |  |  |  |  |
| Vasopressin | Increase | 58.1 (43.5-59.7) | 45.2 (35.5-51.6) | 0.621 | 1 | 17.7 (9.68-22.6) | 9.68 (3.23-45.2) | 1 | 1 |  |  |  |  |  |
| Vasopressin | Decrease | 29 (14.5-33.9) | 64.5 (32.3-69.4) | 0.507 | 1 | 3.23 (1.61-8.06) | 54.8 (27.4-56.5) | 0.507 | 1 |  |  |  |  |  |
| **Bolus** | | | | | | | | | | | | | | |
| **Name** | **Doses** | **Mean MAP** | | | | **Mean ICP** | | | | **Mean rSO2_R** | | | |  |
|  |  | **Pre Dose** | **Post Dose** | **P value** | **Adj P Value** | **Pre Dose** | **Post Dose** | **P value** | **Adj P Value** | **Pre Dose** | **Post Dose** | **P value** | **Adj P Value** |  |
| Fentanyl | 32 | 84 (75.9-98.3) | 82.3 (75.3-94.5) | 0.404 | 1 | 15.1 (9.79-19.2) | 14.5 (9.49-21.6) | 0.886 | 1 | 71.4 (65.3-84.9) | 70 (66.1-83.9) | 0.767 | 1 |  |
| Ketamine | 3 | 103 (96.6-105) | 97.1 (96.5-105) | 1 | 1 | 16.3 (11.8-17.7) | 19.9 (18.2-20.2) | 0.2 | 1 | 69.8 (66.8-71.8) | 70 (67.7-70.8) | 1 | 1 |  |
| Midazolam | 8 | 104 (99.9-106) | 101 (94.4-108) | 1 | 1 | 19.4 (16.7-21.7) | 21.4 (18.6-22.3) | 0.574 | 1 | 67.8 (66.6-70.2) | 68.2 (65.4-69.9) | 0.878 | 1 |  |
| **Name** | **Mean rSO2_L** | | | | **Mean CPP** | | | | **Mean AMP** | | | |  |  |
|  | **Pre Dose** | **Post Dose** | **P value** | **Adj P Value** | **Pre Dose** | **Post Dose** | **P value** | **Adj P Value** | **Pre Dose** | **Post Dose** | **P value** | **Adj P Value** |  |  |
| Fentanyl | 68.3 (64.7-74.1) | 68.8 (64.9-74) | 0.898 | 1 | 70.9 (63.5-78.6) | 69.4 (62.2-74.5) | 0.284 | 1 | 4.06 (1.85-6.88) | 4.31 (2.02-7.65) | 0.757 | 1 |  |  |
| Ketamine | 66 (65.2-67.5) | 65.7 (65-66.6) | 0.7 | 1 | 84.2 (83.3-86.8) | 79.4 (78-86.3) | 0.7 | 1 | 4.5 (3.73-4.81) | 5.85 (5.67-5.89) | 0.1 | 1 |  |  |
| Midazolam | 64.3 (61.7-67.6) | 63.7 (61.7-66.5) | 0.959 | 1 | 84.3 (78.4-88.2) | 80.6 (75.7-85.4) | 0.574 | 1 | 5.46 (4.77-7.69) | 6.67 (4.96-8.41) | 0.442 | 1 |  |  |
| **Name** | **Mean PRx** | | | | **Mean PAx** | | | | **Mean RAC** | | | |  |  |
|  | **Pre Dose** | **Post Dose** | **P value** | **Adj P Value** | **Pre Dose** | **Post Dose** | **P value** | **Adj P Value** | **Pre Dose** | **Post Dose** | **P value** | **Adj P Value** |  |  |
| Fentanyl | 0.205 (-0.0108-0.396) | 0.173 (-0.00616-0.381) | 0.989 | 1 | 0.141 (-0.0365-0.296) | 0.103 (-0.0305-0.333) | 0.762 | 1 | -0.0465 (-0.232-0.216) | -0.086 (-0.214-0.251) | 0.68 | 1 |  |  |
| Ketamine | -0.0173 (-0.0392-0.195) | 0.205 (-0.0506-0.227) | 1 | 1 | 0.188 (0.109-0.213) | 0.203 (-0.0162-0.243) | 1 | 1 | 0.0766 (-0.0932-0.107) | -0.0547 (-0.269--0.0147) | 0.4 | 1 |  |  |
| Midazolam | -0.169 (-0.217--0.0396) | -0.169 (-0.387-0.0323) | 0.798 | 1 | -0.137 (-0.23-0.0109) | -0.119 (-0.331-0.0014) | 0.878 | 1 | -0.356 (-0.386--0.134) | -0.432 (-0.563--0.279) | 0.442 | 1 |  |  |
| **Name** | **Mean COx_L_a** | | | | **Mean COx_R_a** | | | | **% time ICP > 20** | | | |  |  |
|  | **Pre Dose** | **Post Dose** | **P value** | **Adj P Value** | **Pre Dose** | **Post Dose** | **P value** | **Adj P Value** | **Pre Dose** | **Post Dose** | **P value** | **Adj P Value** |  |  |
| Fentanyl | 0.0589 (0.00828-0.124) | 0.0826 (-0.00586-0.173) | 0.881 | 1 | 0.163 (0.0587-0.242) | 0.162 (0.0121-0.24) | 0.676 | 1 | 0 (0-36.3) | 3.23 (0-54.8) | 0.623 | 1 |  |  |
| Ketamine | 0.0867 (0.03-0.157) | 0.205 (0.109-0.216) | 1 | 1 | 0.0744 (0.0386-0.124) | 0.248 (0.191-0.274) | 0.2 | 1 | 0 (0-14.5) | 22.6 (14.5-51.6) | 0.376 | 1 |  |  |
| Midazolam | 0.0198 (-0.00667-0.0761) | 0.0479 (-0.00495-0.121) | 0.721 | 1 | -0.0248 (-0.0511-0.0971) | 0.0213 (-0.0187-0.0702) | 0.645 | 1 | 41.9 (0-87.9) | 56.5 (33.9-72.6) | 0.916 | 1 |  |  |
| **Name** | **% time ICP > 22** | | | | **% time CPP > 60** | | | | **% time CPP > 70** | | | |  |  |
|  | **Pre Dose** | **Post Dose** | **P value** | **Adj P Value** | **Pre Dose** | **Post Dose** | **P value** | **Adj P Value** | **Pre Dose** | **Post Dose** | **P value** | **Adj P Value** |  |  |
| Fentanyl | 0 (0-4.03) | 0 (0-38.7) | 0.0552 | 1 | 0 (0-17.7) | 8.06 (0-20.2) | 0.238 | 1 | 51.6 (0-93.5) | 30.6 (3.23-91.1) | 0.652 | 1 |  |  |
| Ketamine | 0 (0-3.23) | 0 (0-0) | 0.505 | 1 | 0 (0-3.23) | 0 (0-0) | 0.505 | 1 | 100 (95.2-100) | 100 (88.7-100) | 1 | 1 |  |  |
| Midazolam | 8.06 (0-42.7) | 30.6 (4.84-55.6) | 0.666 | 1 | 0 (0-3.23) | 0 (0-1.61) | 0.898 | 1 | 95.2 (92.7-100) | 98.4 (95.2-100) | 0.582 | 1 |  |  |
| **Name** | **% time PRx > 0** | | | | **% time PRx > 0.25** | | | | **% time PRx > 0.35** | | | |  |  |
|  | **Pre Dose** | **Post Dose** | **P value** | **Adj P Value** | **Pre Dose** | **Post Dose** | **P value** | **Adj P Value** | **Pre Dose** | **Post Dose** | **P value** | **Adj P Value** |  |  |
| Fentanyl | 74.2 (46.8-83.9) | 56.5 (38.7-92.7) | 0.918 | 1 | 38.7 (14.5-69.4) | 38.7 (25.8-80.6) | 0.433 | 1 | 29 (11.3-61.3) | 27.4 (19.4-71.8) | 0.449 | 1 |  |  |
| Ketamine | 71 (50-85.5) | 80.6 (45.2-82.3) | 1 | 1 | 29 (21-59.7) | 38.7 (19.4-50) | 1 | 1 | 12.9 (12.9-43.5) | 22.6 (11.3-35.5) | 1 | 1 |  |  |
| Midazolam | 22.6 (12.9-50.8) | 21 (8.87-47.6) | 0.875 | 1 | 3.23 (2.42-8.87) | 11.3 (0-24.2) | 0.708 | 1 | 0 (0-4.03) | 6.45 (0-20.2) | 0.524 | 1 |  |  |
| **Name** | **% time PAx > 0** | | | | **% time PAx > 0.25** | | | | **% time RAC > -0.1** | | | |  |  |
|  | **Pre Dose** | **Post Dose** | **P value** | **Adj P Value** | **Pre Dose** | **Post Dose** | **P value** | **Adj P Value** | **Pre Dose** | **Post Dose** | **P value** | **Adj P Value** |  |  |
| Fentanyl | 74.2 (41.9-83.9) | 64.5 (47.6-90.3) | 0.659 | 1 | 32.3 (11.3-48.4) | 37.1 (14.5-61.3) | 0.363 | 1 | 45.2 (25.8-75.8) | 51.6 (32.3-84.7) | 0.312 | 1 |  |  |
| Ketamine | 71 (71-79) | 80.6 (46.8-85.5) | 1 | 1 | 41.9 (35.5-50) | 29 (19.4-43.5) | 0.653 | 1 | 74.2 (51.6-79) | 54.8 (32.3-58.1) | 0.4 | 1 |  |  |
| Midazolam | 30.6 (11.3-60.5) | 37.1 (17.7-51.6) | 1 | 1 | 6.45 (0-14.5) | 11.3 (2.42-34.7) | 0.455 | 1 | 9.68 (3.23-55.6) | 14.5 (9.68-26.6) | 0.833 | 1 |  |  |
| **Name** | **% time RAC > -0.05** | | | | **% time COx_R_a > 0** | | | | **% time COx_R_a > 0.3** | | | |  |  |
|  | **Pre Dose** | **Post Dose** | **P value** | **Adj P Value** | **Pre Dose** | **Post Dose** | **P value** | **Adj P Value** | **Pre Dose** | **Post Dose** | **P value** | **Adj P Value** |  |  |
| Fentanyl | 38.7 (19.4-71) | 43.5 (29.8-76.6) | 0.386 | 1 | 67.7 (35.5-74.2) | 64.5 (43.5-77.4) | 0.681 | 1 | 25.8 (12.9-45.2) | 25.8 (14.5-48.4) | 0.928 | 1 |  |  |
| Ketamine | 67.7 (48.4-71) | 38.7 (19.4-48.4) | 0.4 | 1 | 48.4 (40.3-56.5) | 87.1 (80.6-88.7) | 0.1 | 1 | 22.6 (21-30.6) | 35.5 (21-41.9) | 1 | 1 |  |  |
| Midazolam | 3.23 (3.23-37.1) | 11.3 (4.84-21.8) | 0.873 | 1 | 32.3 (27.4-42.7) | 53.2 (21.8-58.9) | 0.916 | 1 | 19.4 (0-28.2) | 9.68 (5.65-16.1) | 0.75 | 1 |  |  |
| **Name** | **% time COx_L_a > 0** | | | | **% time COx_L_a > 0.3** | | | |  | | | |  |  |
|  | **Pre Dose** | **Post Dose** | **P value** | **Adj P Value** | **Pre Dose** | **Post Dose** | **P value** | **Adj P Value** |  |  |  |  |  |  |
| Fentanyl | 48.4 (29-61.3) | 54.8 (25.8-65.3) | 0.771 | 1 | 12.9 (6.45-25.8) | 19.4 (6.45-39.5) | 0.382 | 1 |  |  |  |  |  |  |
| Ketamine | 54.8 (40.3-67.7) | 58.1 (35.5-61.3) | 1 | 1 | 32.3 (21-40.3) | 35.5 (17.7-37.1) | 1 | 1 |  |  |  |  |  |  |
| Midazolam | 27.4 (25-43.5) | 41.9 (16.1-60.5) | 0.636 | 1 | 16.1 (6.45-19.4) | 12.9 (8.87-28.2) | 0.711 | 1 |  |  |  |  |  |  |

*The table demonstrates the median and interquartile range of the pre/pose dose windows as well as the Wilcox signed ranked test between these windows with p-values adjusted using the Bonferroni analysis. The “change” indicates if the continuous infusion was increase/decrease or was a bolus dose. AMP, intracranial pulse amplitude; COx_R_a, cerebral oximetry index of right side using MAP; COx_L_a, cerebral oximetry index of left side using MAP; CPP, cerebral prefusion pressure; ICP, intracranial pressure; MAP, mean arterial blood pressure; PAx, pulse amplitude index; PRx, pressure reactivity; RAC, correlation between intracranial pulse amplitude and CPP; rSO2_L, regional oxygen saturation on left side; rSO2_R, regional oxygen saturation on right side.*

# Appendix F. Age >= 60

The table contains all infusions given with the patient >= 60 years, separated into the continuous infusion doses then the bolus doses.

| **Continuous Infusion** | | | | | | | | | | | | | | |
| --- | --- | --- | --- | --- | --- | --- | --- | --- | --- | --- | --- | --- | --- | --- |
| **Name** | **Doses** | **Mean Dose Change** | **Mean MAP** | | | | **Mean ICP** | | | | **Mean rSO2_R** | | | |
|  |  |  | **Pre Dose** | **Post Dose** | **P value** | **Adj P Value** | **Pre Dose** | **Post Dose** | **P value** | **Adj P Value** | **Pre Dose** | **Post Dose** | **P value** | **Adj P Value** |
| Fentanyl | 136 | Decrease | 75.5 (68.9-84.9) | 78.9 (70.4-87.8) | 0.114 | 1 | 8.74 (4.6-13.3) | 8.81 (4.87-13.2) | 0.92 | 1 | 64 (55.3-71.6) | 63.4 (55.6-71.5) | 0.991 | 1 |
| Fentanyl | 130 | Increase | 78.1 (70-87.1) | 78.1 (70-85.3) | 0.713 | 1 | 11.7 (5.18-14.4) | 10.8 (5.52-14.2) | 0.55 | 1 | 62 (52.4-70.2) | 64.7 (54.2-69.6) | 0.493 | 1 |
| Ketamine | 29 | Increase | 74.9 (72.4-84) | 76.8 (73.8-84.3) | 0.513 | 1 | 8.55 (7.23-18) | 9.7 (7.09-19.2) | 0.805 | 1 | 54.9 (0-60.2) | 55 (0-64.2) | 0.945 | 1 |
| Ketamine | 9 | Decrease | 75.9 (68.2-81.5) | 71.7 (66.8-82.7) | 0.653 | 1 | 5.81 (3.83-9.54) | 6.96 (3.96-9.96) | 0.935 | 1 | 69.2 (59.4-71.1) | 69.6 (56.5-72.8) | 0.902 | 1 |
| Midazolam | 71 | Increase | 73.2 (68.1-82.2) | 75.1 (70.3-79.8) | 0.365 | 1 | 11 (8.54-13.8) | 9.67 (6.47-12.4) | 0.0526 | 1 | 56.8 (38.4-65.4) | 56.1 (36.5-64.1) | 0.828 | 1 |
| Midazolam | 57 | Decrease | 75.3 (67.3-90.6) | 76.5 (68.7-88.3) | 0.642 | 1 | 11.4 (8.57-14.9) | 11.4 (7.2-14) | 0.654 | 1 | 50.2 (0-58.2) | 49.2 (0-57.9) | 0.8 | 1 |
| Norepinephrine | 1232 | Decrease | 79.2 (71.7-87.6) | 78 (71-85.9) | 0.0196 | 0.509 | 10.4 (6.04-14.3) | 10.7 (6.18-14.6) | 0.446 | 1 | 60.9 (53.4-69) | 60.5 (53.4-68.8) | 0.716 | 1 |
| Norepinephrine | 1034 | Increase | 76 (69.5-84) | 76.7 (70.5-84.5) | 0.154 | 1 | 11.1 (6.26-16.1) | 10.8 (6.47-15.6) | 0.88 | 1 | 60.8 (53.7-67.8) | 60.8 (54-68.4) | 0.73 | 1 |
| Phenylephrine | 13 | Increase | 59.7 (0-66.9) | 59.9 (0-62.5) | 1 | 1 | 5.21 (0.366-15.2) | 6.02 (1.51-19.8) | 0.724 | 1 | 64.3 (27.9-80.1) | 60.5 (27.5-79.2) | 0.762 | 1 |
| Phenylephrine | 9 | Decrease | 70.3 (63.8-73.8) | 65.9 (60.4-70) | 0.243 | 1 | 8.36 (5.53-11.3) | 9.85 (7-11.8) | 0.78 | 1 | 77.3 (60.9-79.4) | 78.6 (60.1-81) | 0.853 | 1 |
| Propofol | 364 | Decrease | 78 (69.8-86.2) | 79 (70.5-87.5) | 0.273 | 1 | 8.4 (4.17-12.9) | 8.94 (4.28-13.4) | 0.432 | 1 | 64 (55.2-70.2) | 63.6 (55.4-70.5) | 0.916 | 1 |
| Propofol | 270 | Increase | 78.8 (69.3-89.2) | 77.7 (68.8-87.5) | 0.25 | 1 | 8.78 (3.93-13.1) | 8.56 (4.4-12.6) | 0.831 | 1 | 65.3 (56.1-70.8) | 64.6 (56.4-70.6) | 0.591 | 1 |
| Vasopressin | 53 | Decrease | 78.6 (69.2-86.8) | 79.2 (69.9-87.7) | 0.94 | 1 | 5.98 (4.05-13.3) | 7.75 (5.2-16) | 0.257 | 1 | 54.5 (39.7-64.4) | 56 (43.6-62.9) | 0.817 | 1 |
| Vasopressin | 39 | Increase | 73.1 (68.8-85.3) | 70.4 (67.2-82.5) | 0.469 | 1 | 11.1 (6.07-18.9) | 11 (5.52-15.4) | 0.528 | 1 | 55 (0-64.7) | 52 (0-63.6) | 0.638 | 1 |
| **Name** | **Mean Dose Change** | **Mean rSO2_L** | | | | **Mean CPP** | | | | **Mean AMP** | | | |  |
|  |  | **Pre Dose** | **Post Dose** | **P value** | **Adj P Value** | **Pre Dose** | **Post Dose** | **P value** | **Adj P Value** | **Pre Dose** | **Post Dose** | **P value** | **Adj P Value** |  |
| Fentanyl | Decrease | 66.1 (53.2-72.3) | 65.1 (57.6-73.1) | 0.837 | 1 | 67.9 (60.1-75.4) | 69.8 (62.6-77.4) | 0.157 | 1 | 1.5 (0.947-1.99) | 1.55 (1.03-1.96) | 0.725 | 1 |  |
| Fentanyl | Increase | 65.8 (58.4-72.8) | 66.1 (56.6-71.7) | 0.765 | 1 | 66.4 (61.4-74.2) | 67.2 (60.1-73.7) | 0.849 | 1 | 1.7 (1.14-2.23) | 1.54 (1.04-2.52) | 0.711 | 1 |  |
| Ketamine | Increase | 63.3 (0-68.1) | 64.4 (0-67.3) | 0.937 | 1 | 64.3 (54.2-69.1) | 64.5 (55.4-69.6) | 0.774 | 1 | 1.38 (1.05-3.29) | 1.35 (1-3.05) | 0.968 | 1 |  |
| Ketamine | Decrease | 68.9 (66.8-69.9) | 69.3 (65.7-69.5) | 0.902 | 1 | 68.1 (66.4-71.7) | 67.2 (65.1-72.7) | 0.775 | 1 | 1.62 (1.28-1.81) | 1.48 (1.14-1.71) | 0.744 | 1 |  |
| Midazolam | Increase | 62.9 (57.3-72.8) | 64 (56.7-72.3) | 0.997 | 1 | 61.1 (58.1-67.5) | 66.1 (59.3-71.4) | 0.0426 | 1 | 2.01 (1.19-4.12) | 1.6 (1.13-3.27) | 0.245 | 1 |  |
| Midazolam | Decrease | 62.9 (48.4-66.2) | 61 (46.1-64.5) | 0.338 | 1 | 63.8 (58.3-77.8) | 65.4 (59.1-75.6) | 0.919 | 1 | 1.14 (0.92-2.53) | 1.16 (0.884-2.33) | 0.764 | 1 |  |
| Norepinephrine | Decrease | 64 (55.8-71.5) | 64.1 (56-70.9) | 0.733 | 1 | 69 (61.3-75.9) | 67.8 (60.6-74.1) | 0.00528 | 0.137 | 1.72 (1.08-2.74) | 1.69 (1.08-2.79) | 0.909 | 1 |  |
| Norepinephrine | Increase | 62.7 (55.1-70.4) | 62.9 (55.3-70.1) | 0.776 | 1 | 64.9 (59.3-70.4) | 65.2 (59.6-71.7) | 0.0774 | 1 | 1.83 (1.1-3.28) | 1.87 (1.1-3.42) | 0.596 | 1 |  |
| Phenylephrine | Increase | 61.2 (40.7-82.9) | 61.3 (39.7-80.9) | 0.92 | 1 | 59.1 (0-64.1) | 54.2 (0-64.9) | 0.876 | 1 | 0.74 (0.557-1.65) | 1 (0.579-1.95) | 0.545 | 1 |  |
| Phenylephrine | Decrease | 78.8 (60-83.3) | 82 (60.9-86.3) | 0.842 | 1 | 64.9 (62.8-72.3) | 62.2 (57.5-65.8) | 0.182 | 1 | 0.659 (0.377-0.817) | 0.647 (0.335-0.992) | 1 | 1 |  |
| Propofol | Decrease | 66.7 (58.2-73.1) | 66.8 (58.1-73.3) | 0.78 | 1 | 68.4 (61.8-76.7) | 69.7 (61.3-78.2) | 0.452 | 1 | 1.41 (0.729-2.2) | 1.46 (0.783-2.37) | 0.462 | 1 |  |
| Propofol | Increase | 65.9 (58-72.3) | 64.6 (57.1-72) | 0.479 | 1 | 69.5 (62-77.9) | 68.4 (61.5-76.7) | 0.285 | 1 | 1.68 (0.945-2.5) | 1.59 (0.854-2.45) | 0.678 | 1 |  |
| Vasopressin | Decrease | 62.8 (55.9-67.2) | 63.4 (53.8-68.3) | 0.97 | 1 | 69.1 (62.8-77) | 67.1 (62.9-72.8) | 0.4 | 1 | 1.15 (0.648-1.77) | 1.2 (0.709-1.81) | 0.579 | 1 |  |
| Vasopressin | Increase | 64.7 (58-68) | 64.7 (58-69.8) | 1 | 1 | 63.1 (57.8-68) | 62 (56.8-68.4) | 0.772 | 1 | 1.61 (0.714-2.97) | 1.36 (0.549-3.3) | 0.718 | 1 |  |
| **Name** | **Mean Dose Change** | **Mean PRx** | | | | **Mean PAx** | | | | **Mean RAC** | | | |  |
|  |  | **Pre Dose** | **Post Dose** | **P value** | **Adj P Value** | **Pre Dose** | **Post Dose** | **P value** | **Adj P Value** | **Pre Dose** | **Post Dose** | **P value** | **Adj P Value** |  |
| Fentanyl | Decrease | 0.0727 (-0.0682-0.292) | 0.0763 (-0.07-0.254) | 0.893 | 1 | -0.0681 (-0.236-0.111) | -0.0482 (-0.218-0.0928) | 0.998 | 1 | -0.328 (-0.534--0.125) | -0.299 (-0.542--0.103) | 0.927 | 1 |  |
| Fentanyl | Increase | 0.0308 (-0.109-0.271) | 0.0149 (-0.103-0.221) | 0.64 | 1 | -0.0836 (-0.205-0.106) | -0.0945 (-0.255-0.0315) | 0.378 | 1 | -0.338 (-0.471--0.131) | -0.363 (-0.524--0.131) | 0.375 | 1 |  |
| Ketamine | Increase | 0.129 (-0.158-0.348) | 0.103 (-0.171-0.326) | 0.823 | 1 | 0.00801 (-0.266-0.264) | 0 (-0.243-0.277) | 0.798 | 1 | -0.281 (-0.468--0.0755) | -0.368 (-0.474--0.0984) | 0.737 | 1 |  |
| Ketamine | Decrease | 0.0598 (-0.0437-0.31) | 0.158 (0.0181-0.187) | 0.902 | 1 | -0.0477 (-0.0719--0.00034) | -0.0588 (-0.0773-0.0316) | 0.838 | 1 | -0.188 (-0.366--0.0545) | -0.397 (-0.639-0) | 0.775 | 1 |  |
| Midazolam | Increase | -0.00089 (-0.0913-0.363) | -0.0325 (-0.174-0.245) | 0.117 | 1 | -0.0891 (-0.204-0.157) | -0.166 (-0.261-0.118) | 0.107 | 1 | -0.36 (-0.55--0.164) | -0.371 (-0.46--0.195) | 0.701 | 1 |  |
| Midazolam | Decrease | 0.00207 (-0.0979-0.302) | 0.0359 (-0.0982-0.285) | 0.892 | 1 | -0.0637 (-0.303-0.187) | -0.00232 (-0.181-0.115) | 0.414 | 1 | -0.242 (-0.444--0.0106) | -0.163 (-0.367--0.0161) | 0.642 | 1 |  |
| Norepinephrine | Decrease | 0.0557 (-0.108-0.284) | 0.0603 (-0.102-0.279) | 0.929 | 1 | -0.00448 (-0.204-0.189) | -0.0116 (-0.211-0.181) | 0.669 | 1 | -0.296 (-0.519--0.052) | -0.314 (-0.517--0.0515) | 0.49 | 1 |  |
| Norepinephrine | Increase | 0.0476 (-0.123-0.312) | 0.0238 (-0.133-0.287) | 0.199 | 1 | -0.0385 (-0.217-0.188) | -0.0348 (-0.217-0.195) | 0.589 | 1 | -0.322 (-0.545--0.0084) | -0.319 (-0.531--0.0286) | 0.65 | 1 |  |
| Phenylephrine | Increase | 0.106 (0-0.239) | 0.132 (0-0.283) | 1 | 1 | -0.0777 (-0.353-0) | -0.072 (-0.153-0) | 0.64 | 1 | -0.0972 (-0.349-0) | -0.14 (-0.189-0) | 0.64 | 1 |  |
| Phenylephrine | Decrease | 0.214 (0.131-0.39) | 0.0131 (0-0.15) | 0.0279 | 0.67 | -0.16 (-0.299--0.0982) | -0.265 (-0.382--0.00575) | 0.968 | 1 | -0.219 (-0.366--0.139) | -0.225 (-0.384--0.0972) | 0.968 | 1 |  |
| Propofol | Decrease | 0.123 (-0.0301-0.323) | 0.12 (-0.034-0.318) | 0.844 | 1 | -0.00446 (-0.169-0.158) | 0 (-0.164-0.165) | 0.759 | 1 | -0.279 (-0.463--0.0258) | -0.268 (-0.45--0.0349) | 0.912 | 1 |  |
| Propofol | Increase | 0.131 (-0.0247-0.32) | 0.163 (-0.00706-0.335) | 0.341 | 1 | -0.0058 (-0.187-0.192) | -0.00205 (-0.17-0.192) | 0.508 | 1 | -0.276 (-0.474--0.0527) | -0.225 (-0.471--0.00971) | 0.195 | 1 |  |
| Vasopressin | Decrease | 0.0712 (-0.0865-0.274) | 0.0893 (-0.0498-0.279) | 0.965 | 1 | -0.025 (-0.234-0.134) | -0.00978 (-0.216-0.0891) | 0.709 | 1 | -0.32 (-0.478--0.109) | -0.289 (-0.516--0.126) | 0.818 | 1 |  |
| Vasopressin | Increase | 0.0434 (-0.0557-0.361) | 0.155 (-0.0423-0.32) | 0.656 | 1 | 0 (-0.143-0.134) | -0.0136 (-0.174-0.141) | 0.621 | 1 | -0.27 (-0.432--0.0241) | -0.172 (-0.4--0.0249) | 0.593 | 1 |  |
| **Name** | **Mean Dose Change** | **Mean COx_L_a** | | | | **Mean COx_R_a** | | | | **% time ICP > 20** | | | |  |
|  |  | **Pre Dose** | **Post Dose** | **P value** | **Adj P Value** | **Pre Dose** | **Post Dose** | **P value** | **Adj P Value** | **Pre Dose** | **Post Dose** | **P value** | **Adj P Value** |  |
| Fentanyl | Decrease | 0.0633 (-0.0419-0.195) | 0.0573 (-0.0358-0.15) | 0.281 | 1 | 0.0822 (-0.0368-0.179) | 0 (-0.0886-0.157) | 0.107 | 1 | 0 (0-0) | 0 (0-0) | 0.889 | 1 |  |
| Fentanyl | Increase | 0.0836 (0-0.2) | 0.0461 (-0.0318-0.187) | 0.23 | 1 | 0.0662 (0-0.157) | 0.0222 (-0.0374-0.199) | 0.297 | 1 | 0 (0-8.87) | 0 (0-0) | 0.132 | 1 |  |
| Ketamine | Increase | 0 (-0.0102-0.114) | 0 (0-0.116) | 0.601 | 1 | 0 (0-0.104) | 0 (-0.0194-0.0453) | 0.12 | 1 | 0 (0-4.03) | 0 (0-58.1) | 0.499 | 1 |  |
| Ketamine | Decrease | 0.0753 (-0.0939-0.27) | 0.151 (0.00569-0.205) | 0.902 | 1 | 0.0275 (-0.042-0.153) | 0.0549 (0-0.087) | 1 | 1 | 0 (0-0) | 0 (0-9.68) | 0.252 | 1 |  |
| Midazolam | Increase | 0.103 (-0.00208-0.229) | 0.0136 (-0.018-0.177) | 0.344 | 1 | 0.0737 (-0.0192-0.203) | 0.017 (-0.0615-0.135) | 0.0634 | 1 | 0 (0-0) | 0 (0-0) | 0.0721 | 1 |  |
| Midazolam | Decrease | 0 (0-0.171) | 0 (0-0.0795) | 0.8 | 1 | 0 (-0.0779-0.104) | 0.00825 (-0.0779-0.15) | 0.596 | 1 | 0 (0-3.23) | 0 (0-0) | 0.533 | 1 |  |
| Norepinephrine | Decrease | 0.0433 (-0.02-0.161) | 0.0382 (-0.0198-0.16) | 0.78 | 1 | 0.0352 (-0.0302-0.159) | 0.0256 (-0.037-0.152) | 0.41 | 1 | 0 (0-0) | 0 (0-0) | 0.716 | 1 |  |
| Norepinephrine | Increase | 0.0407 (-0.023-0.155) | 0.0325 (-0.0383-0.158) | 0.382 | 1 | 0.0312 (-0.0181-0.148) | 0.0201 (-0.0494-0.148) | 0.198 | 1 | 0 (0-9.68) | 0 (0-3.23) | 0.397 | 1 |  |
| Phenylephrine | Increase | 0.0339 (0-0.0626) | 0 (0-0.238) | 0.603 | 1 | 0 (-0.062-0.0178) | 0 (0-0.0308) | 0.565 | 1 | 0 (0-16.1) | 0 (0-51.6) | 0.975 | 1 |  |
| Phenylephrine | Decrease | 0.0483 (-0.0819-0.233) | 0.0475 (-0.0689-0.119) | 0.54 | 1 | 0.0392 (-0.0917-0.242) | 0.0308 (-0.0694-0.157) | 0.72 | 1 | 0 (0-0) | 0 (0-0) | 1 | 1 |  |
| Propofol | Decrease | 0.0199 (-0.0531-0.167) | 0.0167 (-0.0509-0.165) | 0.931 | 1 | 0.0212 (-0.0553-0.146) | 0.0215 (-0.0472-0.155) | 0.808 | 1 | 0 (0-0) | 0 (0-0) | 0.201 | 1 |  |
| Propofol | Increase | 0.0385 (-0.0126-0.174) | 0.025 (-0.0311-0.165) | 0.355 | 1 | 0.0287 (-0.0493-0.154) | 0.00783 (-0.0707-0.145) | 0.443 | 1 | 0 (0-0) | 0 (0-0) | 0.277 | 1 |  |
| Vasopressin | Decrease | 0.0205 (0-0.187) | 0 (-0.034-0.0766) | 0.109 | 1 | 0.0499 (-0.0869-0.168) | 0.067 (-0.101-0.196) | 0.978 | 1 | 0 (0-0) | 0 (0-0) | 0.0975 | 1 |  |
| Vasopressin | Increase | 4.35e-05 (0-0.0873) | 0.0429 (0-0.138) | 0.209 | 1 | 0.0152 (-0.0479-0.0991) | 0.017 (-0.00756-0.141) | 0.354 | 1 | 0 (0-25.8) | 0 (0-0) | 0.156 | 1 |  |
| **Name** | **Mean Dose Change** | **% time ICP > 22** | | | | **% time CPP > 60** | | | | **% time CPP > 70** | | | |  |
|  |  | **Pre Dose** | **Post Dose** | **P value** | **Adj P Value** | **Pre Dose** | **Post Dose** | **P value** | **Adj P Value** | **Pre Dose** | **Post Dose** | **P value** | **Adj P Value** |  |
| Fentanyl | Decrease | 0 (0-0) | 0 (0-0) | 0.488 | 1 | 6.45 (0-12.9) | 6.45 (0-12.9) | 0.92 | 1 | 41.9 (3.23-87.1) | 59.7 (12.9-87.1) | 0.11 | 1 |  |
| Fentanyl | Increase | 0 (0-3.23) | 0 (0-0) | 0.0488 | 1 | 9.68 (0-12.9) | 9.68 (0-12.9) | 0.434 | 1 | 43.5 (12.1-87.1) | 48.4 (4.03-87.1) | 0.651 | 1 |  |
| Ketamine | Increase | 0 (0-0) | 0 (0-16.1) | 0.449 | 1 | 12.9 (9.68-45.2) | 12.9 (0-22.6) | 0.758 | 1 | 50 (0-87.1) | 54.8 (0-83.9) | 0.431 | 1 |  |
| Ketamine | Decrease | 0 (0-0) | 0 (0-9.68) | 0.208 | 1 | 4.84 (0-12.9) | 3.23 (0-12.9) | 0.796 | 1 | 69.4 (34.7-87.1) | 54.8 (22.6-87.1) | 0.681 | 1 |  |
| Midazolam | Increase | 0 (0-0) | 0 (0-0) | 0.0763 | 1 | 12.9 (12.9-22.6) | 12.9 (8.06-12.9) | 0.0815 | 1 | 22.6 (0-83.9) | 71 (0-87.1) | 0.107 | 1 |  |
| Midazolam | Decrease | 0 (0-0) | 0 (0-0) | 0.415 | 1 | 12.9 (9.68-12.9) | 12.9 (3.23-12.9) | 0.951 | 1 | 64.5 (0-87.1) | 67.7 (0-87.1) | 0.965 | 1 |  |
| Norepinephrine | Decrease | 0 (0-0) | 0 (0-0) | 0.659 | 1 | 9.68 (0-12.9) | 9.68 (0-12.9) | 0.303 | 1 | 64.5 (12.9-87.1) | 51.6 (6.45-87.1) | 0.000984 | 0.0256 |  |
| Norepinephrine | Increase | 0 (0-0) | 0 (0-0) | 0.349 | 1 | 12.9 (0-16.1) | 12.9 (0-12.9) | 0.00999 | 0.26 | 29 (0-77.4) | 35.5 (0-83.9) | 0.0301 | 0.783 |  |
| Phenylephrine | Increase | 0 (0-0) | 0 (0-51.6) | 0.509 | 1 | 12.9 (12.9-48.4) | 12.9 (12.9-77.4) | 0.574 | 1 | 0 (0-0) | 0 (0-38.7) | 0.616 | 1 |  |
| Phenylephrine | Decrease | 0 (0-0) | 0 (0-0) | 1 | 1 | 11.3 (2.42-39.5) | 12.9 (12.9-32.3) | 0.407 | 1 | 24.2 (1.61-83.1) | 0 (0-0) | 0.11 | 1 |  |
| Propofol | Decrease | 0 (0-0) | 0 (0-0) | 0.318 | 1 | 12.9 (0-12.9) | 9.68 (0-12.9) | 0.46 | 1 | 54.8 (6.45-87.1) | 64.5 (6.45-87.1) | 0.191 | 1 |  |
| Propofol | Increase | 0 (0-0) | 0 (0-0) | 0.404 | 1 | 12.9 (0-12.9) | 12.9 (0-12.9) | 0.781 | 1 | 62.9 (6.45-87.1) | 59.7 (6.45-87.1) | 0.474 | 1 |  |
| Vasopressin | Decrease | 0 (0-0) | 0 (0-0) | 0.163 | 1 | 8.06 (0-12.9) | 12.9 (0-12.9) | 0.15 | 1 | 80.6 (17.7-87.1) | 64.5 (9.68-87.1) | 0.303 | 1 |  |
| Vasopressin | Increase | 0 (0-0.806) | 0 (0-0) | 0.208 | 1 | 12.9 (11.3-14.5) | 12.9 (4.84-12.9) | 0.509 | 1 | 19.4 (0-79) | 9.68 (0-77.4) | 0.527 | 1 |  |
| **Name** | **Mean Dose Change** | **% time PRx > 0** | | | | **% time PRx > 0.25** | | | | **% time PRx > 0.35** | | | |  |
|  |  | **Pre Dose** | **Post Dose** | **P value** | **Adj P Value** | **Pre Dose** | **Post Dose** | **P value** | **Adj P Value** | **Pre Dose** | **Post Dose** | **P value** | **Adj P Value** |  |
| Fentanyl | Decrease | 54.8 (26.6-77.4) | 51.6 (32.3-77.4) | 0.769 | 1 | 25.8 (6.45-54) | 25.8 (6.45-52.4) | 0.762 | 1 | 16.1 (0-41.9) | 19.4 (3.23-38.7) | 0.858 | 1 |  |
| Fentanyl | Increase | 46.8 (19.4-71.8) | 46.8 (22.6-76.6) | 0.954 | 1 | 19.4 (3.23-50) | 19.4 (3.23-38.7) | 0.591 | 1 | 11.3 (0-41.9) | 12.9 (0-32.3) | 0.391 | 1 |  |
| Ketamine | Increase | 54.8 (20.2-87.1) | 61.3 (25.8-87.1) | 0.961 | 1 | 37.1 (3.23-64.5) | 32.3 (0-74.2) | 0.974 | 1 | 16.1 (0-52.4) | 16.1 (0-61.3) | 0.968 | 1 |  |
| Ketamine | Decrease | 56.5 (29.8-87.9) | 61.3 (54.8-77.4) | 1 | 1 | 19.4 (5.65-58.9) | 29 (25.8-35.5) | 0.935 | 1 | 14.5 (3.23-40.3) | 25.8 (9.68-29) | 0.967 | 1 |  |
| Midazolam | Increase | 48.4 (29-77.4) | 38.7 (12.9-79) | 0.285 | 1 | 22.6 (3.23-58.1) | 12.9 (0-51.6) | 0.348 | 1 | 9.68 (0-51.6) | 6.45 (0-37.1) | 0.716 | 1 |  |
| Midazolam | Decrease | 48.4 (12.9-74.2) | 51.6 (25.8-74.2) | 0.916 | 1 | 19.4 (0-58.1) | 22.6 (0-51.6) | 0.854 | 1 | 6.45 (0-48.4) | 16.1 (0-45.2) | 0.671 | 1 |  |
| Norepinephrine | Decrease | 54.8 (25.8-80.6) | 54.8 (25.8-80.6) | 0.951 | 1 | 22.6 (3.23-54.8) | 22.6 (3.23-54.8) | 0.654 | 1 | 12.9 (0-41.9) | 12.9 (0-41.9) | 0.713 | 1 |  |
| Norepinephrine | Increase | 51.6 (22.6-80.6) | 48.4 (22.6-77.4) | 0.26 | 1 | 22.6 (3.23-58.1) | 19.4 (3.23-54.8) | 0.0803 | 1 | 16.1 (0-48.4) | 12.9 (0-41.9) | 0.0643 | 1 |  |
| Phenylephrine | Increase | 64.5 (0-71) | 64.5 (0-87.1) | 1 | 1 | 22.6 (0-54.8) | 35.5 (0-71) | 0.584 | 1 | 6.45 (0-19.4) | 25.8 (0-45.2) | 0.463 | 1 |  |
| Phenylephrine | Decrease | 67.7 (61.3-75.8) | 58.1 (41.9-71) | 0.129 | 1 | 46.8 (29.8-56.5) | 19.4 (9.68-29) | 0.0368 | 0.882 | 33.9 (20.2-41.1) | 6.45 (3.23-22.6) | 0.11 | 1 |  |
| Propofol | Decrease | 64.5 (35.5-83.9) | 64.5 (35.5-87.1) | 0.763 | 1 | 32.3 (9.68-58.1) | 32.3 (6.45-61.3) | 0.974 | 1 | 22.6 (3.23-48.4) | 19.4 (3.23-48.4) | 0.91 | 1 |  |
| Propofol | Increase | 61.3 (35.5-83.9) | 67.7 (38.7-87.1) | 0.305 | 1 | 33.9 (6.45-61.3) | 35.5 (9.68-64.5) | 0.427 | 1 | 19.4 (3.23-48.4) | 25.8 (3.23-54.8) | 0.314 | 1 |  |
| Vasopressin | Decrease | 62.9 (29-83.9) | 51.6 (32.3-80.6) | 0.727 | 1 | 27.4 (3.23-61.3) | 22.6 (3.23-48.4) | 0.814 | 1 | 17.7 (0-45.2) | 12.9 (0-35.5) | 0.751 | 1 |  |
| Vasopressin | Increase | 51.6 (32.3-71) | 71 (32.3-83.9) | 0.286 | 1 | 25.8 (6.45-58.1) | 38.7 (1.61-56.5) | 0.849 | 1 | 12.9 (0-54.8) | 25.8 (0-41.9) | 0.781 | 1 |  |
| **Name** | **Mean Dose Change** | **% time PAx > 0** | | | | **% time PAx > 0.25** | | | | **% time RAC > -0.1** | | | |  |
|  |  | **Pre Dose** | **Post Dose** | **P value** | **Adj P Value** | **Pre Dose** | **Post Dose** | **P value** | **Adj P Value** | **Pre Dose** | **Post Dose** | **P value** | **Adj P Value** |  |
| Fentanyl | Decrease | 35.5 (12.9-61.3) | 35.5 (12.9-61.3) | 0.779 | 1 | 11.3 (0-32.3) | 9.68 (0-29) | 0.857 | 1 | 17.7 (9.68-45.2) | 22.6 (9.68-51.6) | 0.747 | 1 |  |
| Fentanyl | Increase | 32.3 (16.1-61.3) | 29 (12.9-51.6) | 0.467 | 1 | 12.9 (0-29) | 6.45 (0-25.8) | 0.299 | 1 | 16.1 (9.68-36.3) | 16.1 (9.68-38.7) | 0.69 | 1 |  |
| Ketamine | Increase | 50 (8.87-75.8) | 54.8 (6.45-80.6) | 0.835 | 1 | 12.9 (0-48.4) | 9.68 (0-58.1) | 0.942 | 1 | 19.4 (12.9-53.2) | 19.4 (12.9-51.6) | 0.981 | 1 |  |
| Ketamine | Decrease | 38.7 (27.4-44.4) | 32.3 (25.8-41.9) | 0.566 | 1 | 8.06 (3.23-27.4) | 12.9 (0-22.6) | 0.804 | 1 | 32.3 (8.06-50.8) | 12.9 (0-32.3) | 0.592 | 1 |  |
| Midazolam | Increase | 32.3 (19.4-64.5) | 22.6 (6.45-62.9) | 0.2 | 1 | 12.9 (0-41.9) | 6.45 (0-27.4) | 0.246 | 1 | 12.9 (12.9-41.9) | 16.1 (12.9-45.2) | 0.637 | 1 |  |
| Midazolam | Decrease | 29 (0-64.5) | 45.2 (22.6-64.5) | 0.229 | 1 | 9.68 (0-32.3) | 16.1 (0-32.3) | 0.804 | 1 | 22.6 (12.9-48.4) | 25.8 (12.9-61.3) | 0.3 | 1 |  |
| Norepinephrine | Decrease | 41.9 (12.9-71) | 41.9 (12.9-71) | 0.854 | 1 | 12.9 (0-41.9) | 12.9 (0-41.9) | 0.677 | 1 | 22.6 (12.9-54.8) | 19.4 (9.68-51.6) | 0.329 | 1 |  |
| Norepinephrine | Increase | 38.7 (12.9-67.7) | 38.7 (12.9-70.2) | 0.906 | 1 | 12.9 (0-41.9) | 12.9 (0-41.9) | 0.886 | 1 | 19.4 (12.9-54.8) | 19.4 (12.9-51.6) | 0.827 | 1 |  |
| Phenylephrine | Increase | 0 (0-22.6) | 25.8 (0-45.2) | 0.23 | 1 | 0 (0-0) | 0 (0-6.45) | 0.109 | 1 | 12.9 (9.68-35.5) | 32.3 (12.9-51.6) | 0.101 | 1 |  |
| Phenylephrine | Decrease | 22.6 (8.06-41.9) | 6.45 (0-41.9) | 0.457 | 1 | 4.84 (0-15.3) | 0 (0-0) | 0.257 | 1 | 32.3 (14.5-53.2) | 19.4 (0-45.2) | 0.346 | 1 |  |
| Propofol | Decrease | 41.9 (19.4-67.7) | 45.2 (19.4-71) | 0.446 | 1 | 16.1 (3.23-32.3) | 12.9 (0-38.7) | 0.835 | 1 | 22.6 (12.9-54.8) | 25.8 (12.9-52.4) | 0.903 | 1 |  |
| Propofol | Increase | 41.9 (12.9-67.7) | 38.7 (19.4-73.4) | 0.627 | 1 | 12.9 (0-38.7) | 14.5 (0-45.2) | 0.653 | 1 | 22.6 (9.68-51.6) | 25.8 (12.9-54.8) | 0.188 | 1 |  |
| Vasopressin | Decrease | 41.9 (9.68-66.9) | 38.7 (16.1-64.5) | 0.682 | 1 | 8.06 (0-31.5) | 6.45 (0-25.8) | 0.725 | 1 | 16.1 (8.06-44.4) | 19.4 (9.68-38.7) | 0.858 | 1 |  |
| Vasopressin | Increase | 41.9 (9.68-69.4) | 29 (16.1-67.7) | 0.634 | 1 | 12.9 (0-38.7) | 9.68 (0-30.6) | 0.295 | 1 | 19.4 (12.9-45.2) | 16.1 (12.9-53.2) | 0.845 | 1 |  |
| **Name** | **Mean Dose Change** | **% time RAC > -0.05** | | | | **% time COx_R_a > 0** | | | | **% time COx_R_a > 0.3** | | | |  |
|  |  | **Pre Dose** | **Post Dose** | **P value** | **Adj P Value** | **Pre Dose** | **Post Dose** | **P value** | **Adj P Value** | **Pre Dose** | **Post Dose** | **P value** | **Adj P Value** |  |
| Fentanyl | Decrease | 16.1 (9.68-37.9) | 16.1 (8.87-39.5) | 0.883 | 1 | 51.6 (29-71) | 45.2 (22.6-64.5) | 0.282 | 1 | 19.4 (6.45-35.5) | 12.9 (0-29) | 0.0753 | 1 |  |
| Fentanyl | Increase | 16.1 (9.68-35.5) | 12.9 (9.68-35.5) | 0.544 | 1 | 50 (33.1-67.7) | 48.4 (25.8-67.7) | 0.734 | 1 | 19.4 (6.45-34.7) | 16.1 (0-38.7) | 0.39 | 1 |  |
| Ketamine | Increase | 14.5 (12.9-49.2) | 16.1 (12.9-48.4) | 0.877 | 1 | 41.9 (0-61.3) | 25.8 (0-54.8) | 0.457 | 1 | 0 (0-16.1) | 0 (0-16.1) | 0.793 | 1 |  |
| Ketamine | Decrease | 29 (3.23-42.7) | 9.68 (0-29) | 0.557 | 1 | 53.2 (25.8-70.2) | 48.4 (35.5-58.1) | 0.651 | 1 | 16.1 (0-25) | 16.1 (6.45-22.6) | 1 | 1 |  |
| Midazolam | Increase | 12.9 (12.9-40.3) | 16.1 (12.9-38.7) | 0.831 | 1 | 51.6 (33.9-72.6) | 48.4 (25.8-67.7) | 0.188 | 1 | 19.4 (6.45-41.9) | 12.9 (1.61-29) | 0.0656 | 1 |  |
| Midazolam | Decrease | 16.1 (12.9-48.4) | 25.8 (12.9-51.6) | 0.238 | 1 | 41.9 (19.4-61.3) | 41.9 (25.8-61.3) | 0.611 | 1 | 9.68 (0-29) | 16.1 (0-29) | 0.403 | 1 |  |
| Norepinephrine | Decrease | 19.4 (9.68-48.4) | 16.1 (9.68-48.4) | 0.361 | 1 | 48.4 (25.8-69.4) | 48.4 (25.8-67.7) | 0.453 | 1 | 12.9 (0-29) | 12.9 (0-29) | 0.382 | 1 |  |
| Norepinephrine | Increase | 16.1 (9.68-48.4) | 16.1 (9.68-48.4) | 0.723 | 1 | 48.4 (22.6-64.5) | 45.2 (19.4-64.5) | 0.371 | 1 | 12.9 (0-29) | 9.68 (0-29) | 0.178 | 1 |  |
| Phenylephrine | Increase | 12.9 (6.45-29) | 29 (12.9-41.9) | 0.112 | 1 | 29 (0-32.3) | 16.1 (0-25.8) | 0.495 | 1 | 0 (0-3.23) | 0 (0-12.9) | 0.925 | 1 |  |
| Phenylephrine | Decrease | 25.8 (12.9-46.8) | 12.9 (0-38.7) | 0.366 | 1 | 38.7 (19.4-48.4) | 32.3 (29-51.6) | 1 | 1 | 21 (3.23-39.5) | 6.45 (0-29) | 0.506 | 1 |  |
| Propofol | Decrease | 19.4 (9.68-48.4) | 19.4 (9.68-45.2) | 0.906 | 1 | 48.4 (22.6-67.7) | 45.2 (25.8-64.5) | 0.778 | 1 | 16.1 (0-32.3) | 12.9 (0-32.3) | 0.946 | 1 |  |
| Propofol | Increase | 19.4 (9.68-41.9) | 19.4 (12.9-48.4) | 0.216 | 1 | 48.4 (25.8-64.5) | 45.2 (22.6-64.5) | 0.512 | 1 | 12.9 (0-32.3) | 12.9 (0-29) | 0.508 | 1 |  |
| Vasopressin | Decrease | 12.9 (7.26-34.7) | 12.9 (6.45-35.5) | 0.736 | 1 | 54.8 (25.8-74.2) | 54.8 (29-71) | 0.816 | 1 | 12.9 (0-35.5) | 16.1 (0-35.5) | 0.644 | 1 |  |
| Vasopressin | Increase | 16.1 (12.9-41.9) | 16.1 (9.68-46.8) | 0.932 | 1 | 41.9 (19.4-61.3) | 51.6 (29-75.8) | 0.263 | 1 | 9.68 (0-22.6) | 12.9 (0-25.8) | 0.772 | 1 |  |
| **Name** | **Mean Dose Change** | **% time COx_L_a > 0** | | | | **% time COx_L_a > 0.3** | | | |  | | | |  |
|  |  | **Pre Dose** | **Post Dose** | **P value** | **Adj P Value** | **Pre Dose** | **Post Dose** | **P value** | **Adj P Value** |  |  |  |  |  |
| Fentanyl | Decrease | 53.2 (35.5-73.4) | 54.8 (33.1-67.7) | 0.426 | 1 | 19.4 (6.45-35.5) | 16.1 (3.23-32.3) | 0.245 | 1 |  |  |  |  |  |
| Fentanyl | Increase | 54.8 (36.3-67.7) | 51.6 (31.5-71) | 0.671 | 1 | 22.6 (9.68-38.7) | 19.4 (2.42-36.3) | 0.184 | 1 |  |  |  |  |  |
| Ketamine | Increase | 35.5 (0-51.6) | 45.2 (0-64.5) | 0.4 | 1 | 3.23 (0-19.4) | 6.45 (0-25.8) | 0.904 | 1 |  |  |  |  |  |
| Ketamine | Decrease | 45.2 (26.6-71) | 61.3 (25.8-71) | 1 | 1 | 21 (0-46.8) | 29 (9.68-32.3) | 0.868 | 1 |  |  |  |  |  |
| Midazolam | Increase | 58.1 (28.2-71) | 41.9 (22.6-66.9) | 0.371 | 1 | 22.6 (6.45-42.7) | 14.5 (0-32.3) | 0.0528 | 1 |  |  |  |  |  |
| Midazolam | Decrease | 25.8 (0-61.3) | 32.3 (0-56.5) | 0.891 | 1 | 9.68 (0-33.9) | 3.23 (0-25.8) | 0.466 | 1 |  |  |  |  |  |
| Norepinephrine | Decrease | 48.4 (29-67.7) | 48.4 (29-67.7) | 0.783 | 1 | 16.1 (3.23-29) | 16.1 (0-29) | 0.577 | 1 |  |  |  |  |  |
| Norepinephrine | Increase | 51.6 (29-67.7) | 48.4 (25.8-66.9) | 0.336 | 1 | 16.1 (3.23-29) | 12.9 (0-32.3) | 0.516 | 1 |  |  |  |  |  |
| Phenylephrine | Increase | 16.1 (0-29) | 32.3 (0-64.5) | 0.291 | 1 | 3.23 (0-6.45) | 3.23 (0-35.5) | 0.433 | 1 |  |  |  |  |  |
| Phenylephrine | Decrease | 41.9 (23.4-61.3) | 29 (16.1-35.5) | 0.204 | 1 | 24.2 (13.7-34.7) | 9.68 (0-16.1) | 0.0747 | 1 |  |  |  |  |  |
| Propofol | Decrease | 48.4 (22.6-67.7) | 48.4 (25.8-67.7) | 0.963 | 1 | 16.1 (3.23-32.3) | 16.1 (0-32.3) | 0.672 | 1 |  |  |  |  |  |
| Propofol | Increase | 51.6 (29-71) | 48.4 (29-66.9) | 0.392 | 1 | 12.9 (0-32.3) | 12.9 (0-32.3) | 0.409 | 1 |  |  |  |  |  |
| Vasopressin | Decrease | 48.4 (0-71) | 35.5 (4.84-60.5) | 0.182 | 1 | 16.1 (0-25.8) | 6.45 (0-27.4) | 0.571 | 1 |  |  |  |  |  |
| Vasopressin | Increase | 40.3 (0-62.1) | 50 (0-75) | 0.612 | 1 | 6.45 (0-29) | 14.5 (0-25.8) | 0.679 | 1 |  |  |  |  |  |
| **Bolus** | | | | | | | | | | | | | | |
| **Name** | **Doses** | **Mean MAP** | | | | **Mean ICP** | | | | **Mean rSO2_R** | | | |  |
|  |  | **Pre Dose** | **Post Dose** | **P value** | **Adj P Value** | **Pre Dose** | **Post Dose** | **P value** | **Adj P Value** | **Pre Dose** | **Post Dose** | **P value** | **Adj P Value** |  |
| Fentanyl | 135 | 80 (71.7-87.9) | 79.3 (72.2-86.6) | 0.571 | 1 | 11.3 (5.81-15.1) | 10.9 (5.82-14.4) | 0.668 | 1 | 64 (56-70.6) | 65.1 (55.8-70.5) | 0.68 | 1 |  |
| Ketamine | 10 | 77.2 (73-78.7) | 78.1 (74.4-83.1) | 0.438 | 1 | 20.8 (8.46-21.8) | 15.7 (9.13-21) | 0.775 | 1 | 0 (0-70.1) | 0 (0-69.1) | 0.884 | 1 |  |
| Midazolam | 35 | 71.9 (68.6-80.2) | 74.2 (69.7-83) | 0.385 | 1 | 11 (8.36-13.5) | 9.44 (6.53-11.8) | 0.142 | 1 | 55.7 (40.6-62.1) | 54.8 (38.7-62.3) | 0.93 | 1 |  |
| Propofol | 3 | 83.7 (64.3-88) | 83.7 (78.1-84.2) | 1 | 1 | 14 (12.2-18.8) | 11 (10.7-17.3) | 0.825 | 1 | 70.6 (70.6-73.3) | 71.9 (71.3-73.1) | 1 | 1 |  |
| **Name** | **Mean rSO2_L** | | | | **Mean CPP** | | | | **Mean AMP** | | | |  |  |
|  | **Pre Dose** | **Post Dose** | **P value** | **Adj P Value** | **Pre Dose** | **Post Dose** | **P value** | **Adj P Value** | **Pre Dose** | **Post Dose** | **P value** | **Adj P Value** |  |  |
| Fentanyl | 67.4 (59.3-73.1) | 67 (58.6-73.1) | 0.808 | 1 | 68.8 (62.3-75.9) | 68.4 (61.6-76) | 0.554 | 1 | 1.86 (1.23-2.47) | 1.8 (1.19-2.68) | 0.853 | 1 |  |  |
| Ketamine | 0 (0-68.2) | 0 (0-67.8) | 0.884 | 1 | 58.1 (54.8-67.7) | 63.1 (58.6-70.3) | 0.438 | 1 | 1.06 (0.994-1.2) | 1.12 (0.969-1.32) | 0.838 | 1 |  |  |
| Midazolam | 60.7 (56.4-67.8) | 62.3 (55.8-67.5) | 0.995 | 1 | 61.1 (58.1-65.6) | 66.3 (59.5-70.5) | 0.0761 | 1 | 1.77 (1.16-3.68) | 1.59 (1.11-3.22) | 0.445 | 1 |  |  |
| Propofol | 66.1 (65.5-69.4) | 66 (65.5-69.4) | 0.825 | 1 | 60.1 (45.4-71) | 61.5 (60.8-67.9) | 0.825 | 1 | 3.85 (2.96-5.26) | 4.26 (2.99-5.47) | 1 | 1 |  |  |
| **Name** | **Mean PRx** | | | | **Mean PAx** | | | | **Mean RAC** | | | |  |  |
|  | **Pre Dose** | **Post Dose** | **P value** | **Adj P Value** | **Pre Dose** | **Post Dose** | **P value** | **Adj P Value** | **Pre Dose** | **Post Dose** | **P value** | **Adj P Value** |  |  |
| Fentanyl | 0.00644 (-0.153-0.261) | 0 (-0.122-0.235) | 0.877 | 1 | -0.0836 (-0.212-0.106) | -0.0914 (-0.255-0.0294) | 0.719 | 1 | -0.365 (-0.541--0.165) | -0.391 (-0.532--0.123) | 0.755 | 1 |  |  |
| Ketamine | 0.256 (-0.0522-0.413) | 0.23 (-0.052-0.392) | 0.967 | 1 | 0.0755 (-0.22-0.468) | 0.255 (-0.109-0.395) | 0.838 | 1 | -0.0517 (-0.357-0.352) | -0.0634 (-0.305-0.279) | 0.967 | 1 |  |  |
| Midazolam | -0.0153 (-0.121-0.453) | -0.0325 (-0.227-0.222) | 0.324 | 1 | -0.102 (-0.192-0.282) | -0.166 (-0.268-0.159) | 0.33 | 1 | -0.36 (-0.569--0.164) | -0.371 (-0.47--0.176) | 0.742 | 1 |  |  |
| Propofol | -0.0794 (-0.0996-0.263) | -0.172 (-0.246--0.126) | 0.268 | 1 | -0.0627 (-0.0841-0.183) | -0.108 (-0.137--0.0855) | 0.268 | 1 | -0.616 (-0.627--0.0976) | -0.483 (-0.56--0.312) | 1 | 1 |  |  |
| **Name** | **Mean COx_L_a** | | | | **Mean COx_R_a** | | | | **% time ICP > 20** | | | |  |  |
|  | **Pre Dose** | **Post Dose** | **P value** | **Adj P Value** | **Pre Dose** | **Post Dose** | **P value** | **Adj P Value** | **Pre Dose** | **Post Dose** | **P value** | **Adj P Value** |  |  |
| Fentanyl | 0.0983 (0-0.218) | 0.0422 (-0.0448-0.18) | 0.0483 | 1 | 0.0648 (0-0.183) | 0.0256 (-0.0359-0.178) | 0.291 | 1 | 0 (0-9.68) | 0 (0-0) | 0.0547 | 1 |  |  |
| Ketamine | 0 (0-0.0637) | 0 (0-0.0742) | 0.961 | 1 | 0 (0-0.124) | 0 (0-0) | 0.133 | 1 | 87.1 (0-87.1) | 38.7 (0-87.1) | 0.691 | 1 |  |  |
| Midazolam | 0.105 (0-0.216) | 0.0658 (-0.018-0.18) | 0.522 | 1 | 0.0708 (-0.0256-0.192) | 0.017 (-0.0603-0.12) | 0.277 | 1 | 0 (0-0) | 0 (0-0) | 0.109 | 1 |  |  |
| Propofol | 0.124 (0.124-0.237) | -0.000913 (-0.0519-0.0617) | 0.268 | 1 | 0.0172 (0.00643-0.178) | -0.038 (-0.0984--0.0212) | 0.121 | 1 | 0 (0-43.5) | 0 (0-43.5) | 1 | 1 |  |  |
| **Name** | **% time ICP > 22** | | | | **% time CPP > 60** | | | | **% time CPP > 70** | | | |  |  |
|  | **Pre Dose** | **Post Dose** | **P value** | **Adj P Value** | **Pre Dose** | **Post Dose** | **P value** | **Adj P Value** | **Pre Dose** | **Post Dose** | **P value** | **Adj P Value** |  |  |
| Fentanyl | 0 (0-3.23) | 0 (0-0) | 0.0124 | 0.321 | 9.68 (0-12.9) | 9.68 (0-12.9) | 0.779 | 1 | 51.6 (16.1-87.1) | 48.4 (6.45-87.1) | 0.843 | 1 |  |  |
| Ketamine | 87.1 (0-87.1) | 24.2 (0-87.1) | 0.691 | 1 | 12.9 (0-41.9) | 12.9 (0-12.9) | 0.765 | 1 | 0 (0-16.1) | 38.7 (0-59.7) | 0.489 | 1 |  |  |
| Midazolam | 0 (0-0) | 0 (0-0) | 0.111 | 1 | 12.9 (12.9-17.7) | 12.9 (12.9-12.9) | 0.218 | 1 | 25.8 (1.61-82.3) | 77.4 (3.23-87.1) | 0.197 | 1 |  |  |
| Propofol | 0 (0-35.5) | 0 (0-35.5) | 1 | 1 | 22.6 (17.7-41.9) | 22.6 (17.7-29) | 1 | 1 | 38.7 (29-62.9) | 51.6 (45.2-69.4) | 0.653 | 1 |  |  |
| **Name** | **% time PRx > 0** | | | | **% time PRx > 0.25** | | | | **% time PRx > 0.35** | | | |  |  |
|  | **Pre Dose** | **Post Dose** | **P value** | **Adj P Value** | **Pre Dose** | **Post Dose** | **P value** | **Adj P Value** | **Pre Dose** | **Post Dose** | **P value** | **Adj P Value** |  |  |
| Fentanyl | 45.2 (20.2-71) | 48.4 (22.6-74.2) | 0.745 | 1 | 16.1 (3.23-48.4) | 19.4 (3.23-45.2) | 0.715 | 1 | 9.68 (0-41.1) | 9.68 (0-32.3) | 0.392 | 1 |  |  |
| Ketamine | 58.1 (32.3-87.1) | 62.9 (41.9-83.1) | 0.771 | 1 | 38.7 (9.68-77.4) | 46.8 (9.68-71) | 1 | 1 | 38.7 (3.23-61.3) | 46.8 (3.23-60.5) | 0.967 | 1 |  |  |
| Midazolam | 41.9 (25.8-82.3) | 35.5 (9.68-74.2) | 0.365 | 1 | 16.1 (1.61-61.3) | 16.1 (0-51.6) | 0.753 | 1 | 6.45 (0-58.1) | 6.45 (0-33.9) | 0.995 | 1 |  |  |
| Propofol | 29 (29-51.6) | 25.8 (17.7-27.4) | 0.164 | 1 | 3.23 (1.61-33.9) | 3.23 (1.61-8.06) | 1 | 1 | 0 (0-32.3) | 0 (0-4.84) | 1 | 1 |  |  |
| **Name** | **% time PAx > 0** | | | | **% time PAx > 0.25** | | | | **% time RAC > -0.1** | | | |  |  |
|  | **Pre Dose** | **Post Dose** | **P value** | **Adj P Value** | **Pre Dose** | **Post Dose** | **P value** | **Adj P Value** | **Pre Dose** | **Post Dose** | **P value** | **Adj P Value** |  |  |
| Fentanyl | 32.3 (13.7-61.3) | 29 (12.9-50) | 0.646 | 1 | 9.68 (0-29) | 6.45 (0-25.8) | 0.467 | 1 | 12.9 (9.68-35.5) | 16.1 (9.68-35.5) | 0.741 | 1 |  |  |
| Ketamine | 51.6 (16.1-87.1) | 69.4 (31.5-83.1) | 0.902 | 1 | 35.5 (9.68-83.9) | 51.6 (10.5-72.6) | 1 | 1 | 58.1 (12.9-100) | 59.7 (29-89.5) | 0.869 | 1 |  |  |
| Midazolam | 32.3 (8.06-69.4) | 22.6 (6.45-71) | 0.536 | 1 | 12.9 (0-50) | 6.45 (0-30.6) | 0.562 | 1 | 12.9 (12.9-45.2) | 16.1 (12.9-46.8) | 0.684 | 1 |  |  |
| Propofol | 35.5 (30.6-61.3) | 35.5 (29-41.9) | 0.825 | 1 | 6.45 (3.23-37.1) | 6.45 (3.23-14.5) | 1 | 1 | 12.9 (12.9-51.6) | 12.9 (12.9-29) | 1 | 1 |  |  |
| **Name** | **% time RAC > -0.05** | | | | **% time COx_R_a > 0** | | | | **% time COx_R_a > 0.3** | | | |  |  |
|  | **Pre Dose** | **Post Dose** | **P value** | **Adj P Value** | **Pre Dose** | **Post Dose** | **P value** | **Adj P Value** | **Pre Dose** | **Post Dose** | **P value** | **Adj P Value** |  |  |
| Fentanyl | 12.9 (6.45-29) | 12.9 (9.68-32.3) | 0.895 | 1 | 51.6 (34.7-71.8) | 48.4 (29-69.4) | 0.614 | 1 | 19.4 (6.45-36.3) | 16.1 (0-38.7) | 0.399 | 1 |  |  |
| Ketamine | 58.1 (12.9-96.8) | 53.2 (24.2-86.3) | 0.902 | 1 | 0 (0-64.5) | 0 (0-45.2) | 0.593 | 1 | 0 (0-16.1) | 0 (0-9.68) | 0.481 | 1 |  |  |
| Midazolam | 12.9 (12.9-43.5) | 16.1 (12.9-45.2) | 0.804 | 1 | 48.4 (35.5-69.4) | 48.4 (29-54.8) | 0.259 | 1 | 22.6 (4.84-40.3) | 12.9 (1.61-27.4) | 0.143 | 1 |  |  |
| Propofol | 12.9 (12.9-50) | 12.9 (12.9-27.4) | 1 | 1 | 54.8 (43.5-64.5) | 32.3 (30.6-38.7) | 0.268 | 1 | 0 (0-27.4) | 9.68 (4.84-11.3) | 1 | 1 |  |  |
| **Name** | **% time COx_L_a > 0** | | | | **% time COx_L_a > 0.3** | | | |  | | | |  |  |
|  | **Pre Dose** | **Post Dose** | **P value** | **Adj P Value** | **Pre Dose** | **Post Dose** | **P value** | **Adj P Value** |  |  |  |  |  |  |
| Fentanyl | 58.1 (38.7-74.2) | 54.8 (29.8-71) | 0.311 | 1 | 22.6 (6.45-38.7) | 16.1 (3.23-35.5) | 0.103 | 1 |  |  |  |  |  |  |
| Ketamine | 0 (0-58.1) | 0 (0-58.1) | 1 | 1 | 0 (0-22.6) | 0 (0-6.45) | 0.594 | 1 |  |  |  |  |  |  |
| Midazolam | 61.3 (41.9-71) | 45.2 (25-77.4) | 0.529 | 1 | 22.6 (9.68-41.9) | 16.1 (0.806-32.3) | 0.176 | 1 |  |  |  |  |  |  |
| Propofol | 64.5 (64.5-67.7) | 48.4 (40.3-59.7) | 0.5 | 1 | 32.3 (27.4-41.9) | 16.1 (8.06-24.2) | 0.268 | 1 |  |  |  |  |  |  |

*The table demonstrates the median and interquartile range of the pre/pose dose windows as well as the Wilcox signed ranked test between these windows with p-values adjusted using the Bonferroni analysis. The “change” indicates if the continuous infusion was increase/decrease or was a bolus dose. AMP, intracranial pulse amplitude; COx_R_a, cerebral oximetry index of right side using MAP; COx_L_a, cerebral oximetry index of left side using MAP; CPP, cerebral prefusion pressure; ICP, intracranial pressure; MAP, mean arterial blood pressure; PAx, pulse amplitude index; PRx, pressure reactivity; RAC, correlation between intracranial pulse amplitude and CPP; rSO2_L, regional oxygen saturation on left side; rSO2_R, regional oxygen saturation on right side.*

# Appendix G. Females

The table contains all infusions given with the patient sex of female, separated into the continuous infusion doses then the bolus doses.

| **Continuous Infusion** | | | | | | | | | | | | | | |
| --- | --- | --- | --- | --- | --- | --- | --- | --- | --- | --- | --- | --- | --- | --- |
| **Name** | **Doses** | **Mean Dose Change** | **Mean MAP** | | | | **Mean ICP** | | | | **Mean rSO2_R** | | | |
|  |  |  | **Pre Dose** | **Post Dose** | **P value** | **Adj P Value** | **Pre Dose** | **Post Dose** | **P value** | **Adj P Value** | **Pre Dose** | **Post Dose** | **P value** | **Adj P Value** |
| Fentanyl | 28 | Decrease | 79.4 (71.1-89.1) | 79.4 (74.2-90.8) | 0.461 | 1 | 11.6 (7.53-15.5) | 11.8 (9.13-16.4) | 0.632 | 1 | 61.7 (57.4-70.9) | 60.7 (57.3-69.7) | 0.593 | 1 |
| Fentanyl | 24 | Increase | 87.9 (75.5-91.8) | 82.7 (72-90.2) | 0.366 | 1 | 17 (11.7-19.4) | 13.5 (10.4-17.2) | 0.238 | 1 | 61.5 (57.2-68.5) | 61.4 (57.1-68) | 0.964 | 1 |
| Ketamine | 3 | Increase | 89.4 (80.5-89.5) | 74.5 (74.4-81.3) | 0.7 | 1 | 8.41 (5.9-9.9) | 8.94 (6.82-9.32) | 1 | 1 | 71.4 (70.7-74.3) | 71 (70.1-72.6) | 0.7 | 1 |
| Ketamine | 3 | Decrease | 76.5 (76.3-82.6) | 74.8 (73.3-85.8) | 0.7 | 1 | 7.6 (6.33-8.89) | 6.96 (5.46-8.24) | 0.7 | 1 | 73.8 (72.7-74.5) | 74.2 (73.5-74.9) | 0.7 | 1 |
| Midazolam | 9 | Increase | 75.4 (71.9-79.2) | 79.2 (74.2-83) | 0.423 | 1 | 13.2 (11.8-14.2) | 9.43 (8.07-17.6) | 0.247 | 1 | 56.2 (53.6-57.4) | 58.4 (54.6-60) | 0.285 | 1 |
| Norepinephrine | 316 | Decrease | 79.2 (69.6-88.6) | 78.6 (70.2-86.1) | 0.253 | 1 | 11.6 (8.82-15.8) | 11.9 (8.8-16.1) | 0.578 | 1 | 58 (51.2-67.4) | 57.9 (51.2-66.6) | 0.648 | 1 |
| Norepinephrine | 309 | Increase | 78.4 (70.7-87) | 78.3 (71.2-89) | 0.456 | 1 | 12.9 (9.42-19.1) | 12.5 (9.33-18.7) | 0.665 | 1 | 58.1 (51.3-65.9) | 58.3 (51.2-66) | 0.651 | 1 |
| Phenylephrine | 6 | Increase | 0 (0-49.3) | 0 (0-44.8) | 1 | 1 | 15.5 (10.4-20.6) | 19.8 (12.4-21.3) | 1 | 1 | 27.3 (6.7-27.9) | 26 (6.46-27.2) | 0.37 | 1 |
| Propofol | 68 | Decrease | 80.5 (72.1-87.5) | 79.3 (74.4-86) | 0.967 | 1 | 10.6 (4.7-13.5) | 11.6 (6.7-14.7) | 0.456 | 1 | 64.1 (57.4-69.3) | 64.4 (57.5-70.5) | 0.821 | 1 |
| Propofol | 49 | Increase | 82.5 (71-91.7) | 80.1 (71.3-87) | 0.493 | 1 | 12.6 (10.6-17.3) | 12 (9.86-15.3) | 0.324 | 1 | 63.5 (57.2-69.1) | 64.3 (57.7-68.5) | 0.882 | 1 |
| Vasopressin | 11 | Decrease | 84.5 (75.2-88.8) | 85.8 (69.9-88.9) | 0.922 | 1 | 11.2 (8.38-17.9) | 16.8 (7.46-18) | 0.818 | 1 | 54.5 (52.5-59.3) | 53.8 (50.9-60.7) | 0.805 | 1 |
| Vasopressin | 8 | Increase | 82.7 (72-91.2) | 76.8 (66.9-85.3) | 0.636 | 1 | 18.9 (11.3-19.4) | 18.5 (13.4-19.6) | 0.965 | 1 | 53.4 (45.5-57.1) | 52 (44.9-58.5) | 1 | 1 |
| **Name** | **Mean Dose Change** | **Mean rSO2_L** | | | | **Mean CPP** | | | | **Mean AMP** | | | |  |
|  |  | **Pre Dose** | **Post Dose** | **P value** | **Adj P Value** | **Pre Dose** | **Post Dose** | **P value** | **Adj P Value** | **Pre Dose** | **Post Dose** | **P value** | **Adj P Value** |  |
| Fentanyl | Decrease | 65.5 (61.8-69.5) | 64.5 (61.2-69.7) | 0.957 | 1 | 68.3 (62.1-75.3) | 70.4 (63-75.8) | 0.427 | 1 | 1.41 (1.17-1.8) | 1.41 (1.17-1.82) | 0.886 | 1 |  |
| Fentanyl | Increase | 65.7 (62.4-69.9) | 64.2 (62.1-70.1) | 0.804 | 1 | 66.8 (61.3-73.9) | 67.2 (58.7-72.9) | 0.823 | 1 | 1.86 (1.35-3.02) | 1.83 (1.09-2.36) | 0.358 | 1 |  |
| Ketamine | Increase | 68.2 (68.2-73.7) | 68.8 (68.3-72.2) | 1 | 1 | 78 (72.8-79.5) | 69.6 (67.2-74.4) | 0.7 | 1 | 1.48 (1.34-1.5) | 1.5 (1.36-1.53) | 0.7 | 1 |  |
| Ketamine | Decrease | 68.6 (66.9-69) | 69.5 (67.6-69.5) | 1 | 1 | 68.4 (67.4-76) | 68 (66.6-78.5) | 1 | 1 | 1.59 (1.38-1.59) | 1.38 (1.26-1.51) | 1 | 1 |  |
| Midazolam | Increase | 60.2 (57-60.5) | 60.2 (56-64.3) | 0.929 | 1 | 61.2 (60.1-64.6) | 66.1 (59.3-67.7) | 0.533 | 1 | 1.91 (1.28-2.08) | 1.28 (1.13-1.6) | 0.13 | 1 |  |
| Norepinephrine | Decrease | 61.9 (53.2-68.6) | 61.7 (52.9-68) | 0.705 | 1 | 67.1 (59.5-74.7) | 66.7 (59.8-72.4) | 0.292 | 1 | 1.79 (1.28-2.77) | 1.78 (1.26-2.78) | 0.811 | 1 |  |
| Norepinephrine | Increase | 60.7 (52.5-67.6) | 61 (52.9-68.1) | 0.884 | 1 | 64.7 (57.9-69.2) | 64.9 (59.2-72) | 0.158 | 1 | 2 (1.37-3.61) | 2.05 (1.34-3.53) | 0.899 | 1 |  |
| Phenylephrine | Increase | 40.6 (40.4-43.5) | 39.6 (39-39.7) | 0.0931 | 1 | 0 (0-45.3) | 0 (0-46) | 0.775 | 1 | 2.24 (1.87-2.34) | 2.19 (1.97-2.36) | 1 | 1 |  |
| Propofol | Decrease | 66.9 (58.7-72.5) | 67 (60.2-71.7) | 0.736 | 1 | 68.2 (63-76.9) | 69.6 (63-76.2) | 0.836 | 1 | 1.4 (0.953-1.97) | 1.5 (1.05-2.1) | 0.373 | 1 |  |
| Propofol | Increase | 65.9 (59.5-69.4) | 64.1 (58.4-68.8) | 0.369 | 1 | 67.9 (60.7-74) | 66.3 (62.7-75) | 0.801 | 1 | 1.86 (1.32-2.68) | 1.72 (1.28-2.61) | 0.487 | 1 |  |
| Vasopressin | Decrease | 64.9 (56.8-67.6) | 66.8 (46.1-70) | 0.721 | 1 | 68.9 (67.1-75.4) | 68.8 (66.4-71.4) | 0.49 | 1 | 1.41 (1.21-1.81) | 1.64 (0.999-1.94) | 0.922 | 1 |  |
| Vasopressin | Increase | 52.6 (43.1-63.3) | 54.5 (45.3-60.2) | 0.937 | 1 | 69 (63.6-74.1) | 63.1 (57.9-65.4) | 0.371 | 1 | 1.85 (1.65-2) | 1.97 (1.83-2.36) | 0.825 | 1 |  |
| **Name** | **Mean Dose Change** | **Mean PRx** | | | | **Mean PAx** | | | | **Mean RAC** | | | |  |
|  |  | **Pre Dose** | **Post Dose** | **P value** | **Adj P Value** | **Pre Dose** | **Post Dose** | **P value** | **Adj P Value** | **Pre Dose** | **Post Dose** | **P value** | **Adj P Value** |  |
| Fentanyl | Decrease | 0.0236 (-0.0821-0.237) | -0.0109 (-0.141-0.211) | 0.517 | 1 | 0.00182 (-0.118-0.129) | -0.0175 (-0.166-0.066) | 0.507 | 1 | -0.391 (-0.548--0.0927) | -0.377 (-0.59--0.218) | 0.749 | 1 |  |
| Fentanyl | Increase | -0.0619 (-0.16-0.0158) | -0.0608 (-0.147-0.0568) | 0.71 | 1 | -0.116 (-0.184-0.000392) | -0.0938 (-0.211-0.00082) | 0.975 | 1 | -0.426 (-0.555--0.296) | -0.467 (-0.585--0.136) | 0.89 | 1 |  |
| Ketamine | Increase | -0.0522 (-0.202--0.0426) | -0.0572 (-0.139--0.0468) | 1 | 1 | -0.22 (-0.275--0.102) | -0.159 (-0.21--0.0592) | 0.7 | 1 | -0.357 (-0.446--0.302) | -0.403 (-0.568--0.259) | 1 | 1 |  |
| Ketamine | Decrease | 0.313 (0.088-0.418) | 0.18 (0.169-0.217) | 0.7 | 1 | -0.0867 (-0.152--0.0686) | -0.0773 (-0.143--0.0229) | 0.7 | 1 | -0.176 (-0.45--0.139) | -0.397 (-0.527--0.295) | 0.7 | 1 |  |
| Midazolam | Increase | -0.00479 (-0.0913-0.197) | -0.136 (-0.167--0.00479) | 0.247 | 1 | -0.103 (-0.128-0.165) | -0.141 (-0.194--0.128) | 0.13 | 1 | -0.55 (-0.578--0.428) | -0.42 (-0.428--0.371) | 0.0615 | 1 |  |
| Norepinephrine | Decrease | 0.0739 (-0.108-0.302) | 0.0393 (-0.12-0.328) | 0.908 | 1 | 0.00182 (-0.188-0.185) | -0.0121 (-0.196-0.21) | 0.884 | 1 | -0.264 (-0.538--0.00664) | -0.337 (-0.531-0) | 0.82 | 1 |  |
| Norepinephrine | Increase | 0.0521 (-0.114-0.386) | 0.0454 (-0.119-0.364) | 0.77 | 1 | 0 (-0.208-0.269) | 0.00525 (-0.18-0.276) | 0.569 | 1 | -0.32 (-0.568-0.0619) | -0.301 (-0.542-0.0282) | 0.522 | 1 |  |
| Phenylephrine | Increase | 0 (0-0.176) | 0 (0-0) | 0.391 | 1 | 0 (0-0) | 0 (-0.00346-0) | 0.391 | 1 | 0 (-0.177-0) | 0 (-0.171-0) | 1 | 1 |  |
| Propofol | Decrease | -0.00967 (-0.141-0.132) | -0.00713 (-0.143-0.11) | 0.843 | 1 | -0.0944 (-0.237-0.0616) | -0.0631 (-0.202-0.0632) | 0.491 | 1 | -0.382 (-0.556--0.161) | -0.393 (-0.575--0.111) | 0.931 | 1 |  |
| Propofol | Increase | -0.018 (-0.152-0.157) | -0.0479 (-0.16-0.232) | 0.889 | 1 | -0.0718 (-0.235-0.0395) | -0.0862 (-0.202-0.00328) | 0.95 | 1 | -0.474 (-0.585--0.26) | -0.454 (-0.587--0.21) | 0.624 | 1 |  |
| Vasopressin | Decrease | 0.0828 (-0.0903-0.274) | -0.0071 (-0.116-0.134) | 0.45 | 1 | 0.0928 (-0.0335-0.159) | 0.0424 (-0.137-0.071) | 0.412 | 1 | -0.342 (-0.454--0.14) | -0.305 (-0.585--0.227) | 0.974 | 1 |  |
| Vasopressin | Increase | 0.000282 (-0.114-0.107) | 0.0931 (-0.11-0.359) | 0.713 | 1 | 0.0111 (-0.0503-0.114) | -0.109 (-0.218-0.0732) | 0.43 | 1 | -0.296 (-0.35--0.137) | -0.376 (-0.481--0.0732) | 0.563 | 1 |  |
| **Name** | **Mean Dose Change** | **Mean COx_L_a** | | | | **Mean COx_R_a** | | | | **% time ICP > 20** | | | |  |
|  |  | **Pre Dose** | **Post Dose** | **P value** | **Adj P Value** | **Pre Dose** | **Post Dose** | **P value** | **Adj P Value** | **Pre Dose** | **Post Dose** | **P value** | **Adj P Value** |  |
| Fentanyl | Decrease | 0.0665 (0.00993-0.197) | 0.0269 (-0.0606-0.151) | 0.207 | 1 | 0.166 (0.0143-0.252) | 0.0802 (-0.0805-0.254) | 0.57 | 1 | 0 (0-0) | 0 (0-9.68) | 0.126 | 1 |  |
| Fentanyl | Increase | 0.0615 (0.00314-0.165) | 0.0657 (0-0.163) | 0.973 | 1 | 0.0992 (0.00658-0.291) | 0.0817 (-0.023-0.215) | 0.593 | 1 | 11.3 (0-35.5) | 0 (0-22.6) | 0.14 | 1 |  |
| Ketamine | Increase | 0.131 (0.0976-0.171) | 0.102 (0.0879-0.122) | 1 | 1 | 0.13 (0.127-0.172) | 0.0717 (0.0231-0.0759) | 0.1 | 1 | 0 (0-0) | 0 (0-0) | 1 | 1 |  |
| Ketamine | Decrease | 0.338 (0.0694-0.391) | 0.205 (0.15-0.232) | 0.7 | 1 | 0.327 (0.136-0.342) | 0.109 (0.0827-0.173) | 0.7 | 1 | 0 (0-0) | 0 (0-0) | 1 | 1 |  |
| Midazolam | Increase | 0.23 (-0.101-0.237) | 0.133 (0.11-0.134) | 0.13 | 1 | 0.2 (0.184-0.206) | 0.019 (-0.0158-0.119) | 0.373 | 1 | 0 (0-19.4) | 0 (0-19.4) | 1 | 1 |  |
| Norepinephrine | Decrease | 0.0584 (0-0.172) | 0.0674 (0-0.174) | 0.897 | 1 | 0.0853 (-0.0122-0.208) | 0.0758 (-0.00797-0.196) | 0.591 | 1 | 0 (0-3.23) | 0 (0-3.23) | 0.674 | 1 |  |
| Norepinephrine | Increase | 0.0452 (-0.00864-0.155) | 0.0371 (0-0.16) | 0.745 | 1 | 0.0646 (-0.0269-0.177) | 0.0548 (-0.0471-0.182) | 0.651 | 1 | 0 (0-27.4) | 0 (0-25.8) | 0.464 | 1 |  |
| Phenylephrine | Increase | 0 (0-0) | 0 (0-0) | 1 | 1 | 0 (0-0.0818) | 0 (0-0) | 0.462 | 1 | 21 (6.45-71.8) | 66.1 (12.9-85.5) | 1 | 1 |  |
| Propofol | Decrease | 0.0855 (-0.0042-0.201) | 0.0313 (-0.0499-0.159) | 0.219 | 1 | 0.119 (-0.0235-0.243) | 0.0587 (-0.0506-0.218) | 0.231 | 1 | 0 (0-0) | 0 (0-0) | 0.481 | 1 |  |
| Propofol | Increase | 0.0916 (0.000173-0.185) | 0.108 (0-0.186) | 0.754 | 1 | 0.0646 (-0.016-0.22) | 0.0698 (-0.00907-0.195) | 1 | 1 | 0 (0-12.9) | 0 (0-4.03) | 0.668 | 1 |  |
| Vasopressin | Decrease | 0.035 (-0.0134-0.134) | -0.00731 (-0.0742-0.0699) | 0.503 | 1 | 0.104 (-0.0652-0.302) | 0.115 (-0.0906-0.249) | 0.645 | 1 | 0 (0-0) | 0 (0-19.4) | 0.503 | 1 |  |
| Vasopressin | Increase | 0.015 (0-0.0574) | 0.0561 (0.00676-0.138) | 0.341 | 1 | 0 (0-0.241) | 0 (0-0.102) | 1 | 1 | 22.6 (0-45.2) | 25.8 (0-45.2) | 1 | 1 |  |
| **Name** | **Mean Dose Change** | **% time ICP > 22** | | | | **% time CPP > 60** | | | | **% time CPP > 70** | | | |  |
|  |  | **Pre Dose** | **Post Dose** | **P value** | **Adj P Value** | **Pre Dose** | **Post Dose** | **P value** | **Adj P Value** | **Pre Dose** | **Post Dose** | **P value** | **Adj P Value** |  |
| Fentanyl | Decrease | 0 (0-0) | 0 (0-0) | 0.113 | 1 | 0 (0-12.9) | 8.06 (0-12.9) | 0.527 | 1 | 40.3 (0-87.1) | 69.4 (8.87-87.1) | 0.509 | 1 |  |
| Fentanyl | Increase | 0 (0-21) | 0 (0-9.68) | 0.282 | 1 | 12.9 (9.68-14.5) | 11.3 (0-12.9) | 0.129 | 1 | 35.5 (14.5-71) | 38.7 (0-71) | 0.501 | 1 |  |
| Ketamine | Increase | 0 (0-0) | 0 (0-0) | 1 | 1 | 0 (0-0) | 0 (0-0) | 1 | 1 | 96.8 (56.5-98.4) | 38.7 (19.4-56.5) | 0.4 | 1 |  |
| Ketamine | Decrease | 0 (0-0) | 0 (0-0) | 1 | 1 | 0 (0-1.61) | 0 (0-0) | 0.505 | 1 | 41.9 (25.8-71) | 22.6 (11.3-58.1) | 0.7 | 1 |  |
| Midazolam | Increase | 0 (0-3.23) | 0 (0-9.68) | 0.874 | 1 | 12.9 (12.9-19.4) | 12.9 (12.9-12.9) | 0.396 | 1 | 29 (0-48.4) | 71 (0-83.9) | 0.497 | 1 |  |
| Norepinephrine | Decrease | 0 (0-0) | 0 (0-0) | 0.827 | 1 | 9.68 (0-12.9) | 11.3 (0-12.9) | 0.335 | 1 | 51.6 (6.45-87.1) | 43.5 (3.23-83.9) | 0.134 | 1 |  |
| Norepinephrine | Increase | 0 (0-11.3) | 0 (0-6.45) | 0.741 | 1 | 12.9 (0-19.4) | 12.9 (0-16.1) | 0.0919 | 1 | 19.4 (0-64.5) | 32.3 (0-77.4) | 0.0394 | 1 |  |
| Phenylephrine | Increase | 1.61 (0-66.1) | 51.6 (12.9-78.2) | 0.801 | 1 | 12.9 (10.5-12.9) | 12.9 (12.9-12.9) | 0.595 | 1 | 0 (0-16.9) | 0 (0-29) | 0.775 | 1 |  |
| Propofol | Decrease | 0 (0-0) | 0 (0-0) | 0.448 | 1 | 3.23 (0-12.9) | 0 (0-12.9) | 0.638 | 1 | 48.4 (6.45-93.5) | 71 (16.1-100) | 0.265 | 1 |  |
| Propofol | Increase | 0 (0-0.806) | 0 (0-0) | 0.871 | 1 | 9.68 (0-12.9) | 9.68 (0-12.9) | 0.965 | 1 | 41.9 (3.23-73.4) | 48.4 (3.23-74.2) | 0.902 | 1 |  |
| Vasopressin | Decrease | 0 (0-0) | 0 (0-0) | 0.363 | 1 | 0 (0-6.45) | 12.9 (0-14.5) | 0.0968 | 1 | 48.4 (35.5-88.7) | 58.1 (27.4-72.6) | 0.576 | 1 |  |
| Vasopressin | Increase | 0 (0-9.68) | 0 (0-19.4) | 0.958 | 1 | 12.9 (7.26-15.3) | 12.9 (9.68-24.2) | 0.625 | 1 | 67.7 (9.68-87.9) | 4.84 (0-61.3) | 0.177 | 1 |  |
| **Name** | **Mean Dose Change** | **% time PRx > 0** | | | | **% time PRx > 0.25** | | | | **% time PRx > 0.35** | | | |  |
|  |  | **Pre Dose** | **Post Dose** | **P value** | **Adj P Value** | **Pre Dose** | **Post Dose** | **P value** | **Adj P Value** | **Pre Dose** | **Post Dose** | **P value** | **Adj P Value** |  |
| Fentanyl | Decrease | 50 (27.4-74.2) | 41.9 (25.8-75) | 0.577 | 1 | 21 (3.23-46.8) | 11.3 (3.23-46.8) | 0.699 | 1 | 9.68 (0-35.5) | 8.06 (0-33.1) | 0.82 | 1 |  |
| Fentanyl | Increase | 32.3 (19.4-51.6) | 32.3 (12.1-50) | 0.89 | 1 | 6.45 (0-29) | 1.61 (0-29) | 0.434 | 1 | 3.23 (0-17.7) | 0 (0-16.9) | 0.336 | 1 |  |
| Ketamine | Increase | 32.3 (16.1-37.1) | 41.9 (22.6-41.9) | 0.643 | 1 | 3.23 (1.61-6.45) | 9.68 (4.84-9.68) | 0.814 | 1 | 3.23 (1.61-4.84) | 0 (0-1.61) | 0.48 | 1 |  |
| Ketamine | Decrease | 93.5 (61.3-96.8) | 74.2 (64.5-77.4) | 0.7 | 1 | 51.6 (27.4-74.2) | 35.5 (32.3-43.5) | 0.825 | 1 | 35.5 (17.7-61.3) | 29 (22.6-33.9) | 1 | 1 |  |
| Midazolam | Increase | 35.5 (35.5-61.3) | 12.9 (9.68-35.5) | 0.239 | 1 | 22.6 (0-45.2) | 6.45 (0-22.6) | 0.642 | 1 | 16.1 (0-41.9) | 6.45 (0-16.1) | 0.642 | 1 |  |
| Norepinephrine | Decrease | 54.8 (25.8-80.6) | 51.6 (25.8-83.9) | 0.682 | 1 | 25.8 (6.45-58.1) | 22.6 (3.23-61.3) | 0.878 | 1 | 16.1 (0-48.4) | 16.1 (0-45.2) | 0.8 | 1 |  |
| Norepinephrine | Increase | 54.8 (22.6-83.1) | 51.6 (25.8-80.6) | 0.915 | 1 | 22.6 (3.23-61.3) | 19.4 (3.23-61.3) | 0.516 | 1 | 16.1 (0-54.8) | 12.9 (0-51.6) | 0.34 | 1 |  |
| Phenylephrine | Increase | 0 (0-62.9) | 0 (0-31.5) | 0.775 | 1 | 0 (0-31.5) | 0 (0-2.42) | 0.849 | 1 | 0 (0-24.2) | 0 (0-0) | 0.753 | 1 |  |
| Propofol | Decrease | 41.9 (19.4-67.7) | 41.9 (25-64.5) | 0.727 | 1 | 12.9 (0-35.5) | 12.9 (3.23-33.1) | 0.787 | 1 | 6.45 (0-25.8) | 6.45 (0-21) | 0.949 | 1 |  |
| Propofol | Increase | 41.9 (19.4-69.4) | 35.5 (16.1-64.5) | 0.488 | 1 | 12.9 (3.23-38.7) | 9.68 (0-35.5) | 0.462 | 1 | 4.84 (0-34.7) | 3.23 (0-32.3) | 0.629 | 1 |  |
| Vasopressin | Decrease | 64.5 (37.1-80.6) | 41.9 (32.3-69.4) | 0.43 | 1 | 29 (3.23-51.6) | 12.9 (3.23-29) | 0.488 | 1 | 6.45 (0-41.9) | 9.68 (0-14.5) | 0.629 | 1 |  |
| Vasopressin | Increase | 40.3 (14.5-56.5) | 58.1 (16.9-75.8) | 0.526 | 1 | 4.84 (0-35.5) | 21 (0-56.5) | 0.705 | 1 | 0 (0-27.4) | 16.1 (0-42.7) | 0.562 | 1 |  |
| **Name** | **Mean Dose Change** | **% time PAx > 0** | | | | **% time PAx > 0.25** | | | | **% time RAC > -0.1** | | | |  |
|  |  | **Pre Dose** | **Post Dose** | **P value** | **Adj P Value** | **Pre Dose** | **Post Dose** | **P value** | **Adj P Value** | **Pre Dose** | **Post Dose** | **P value** | **Adj P Value** |  |
| Fentanyl | Decrease | 43.5 (18.5-66.1) | 37.1 (16.1-66.1) | 0.812 | 1 | 12.9 (0-33.1) | 6.45 (2.42-29.8) | 0.636 | 1 | 12.9 (9.68-50) | 12.9 (8.87-24.2) | 0.967 | 1 |  |
| Fentanyl | Increase | 35.5 (21-50) | 22.6 (12.1-36.3) | 0.321 | 1 | 6.45 (0-12.9) | 0 (0-6.45) | 0.141 | 1 | 12.9 (9.68-16.1) | 12.9 (5.65-13.7) | 0.779 | 1 |  |
| Ketamine | Increase | 16.1 (11.3-33.9) | 22.6 (12.9-45.2) | 1 | 1 | 9.68 (6.45-14.5) | 0 (0-16.1) | 0.658 | 1 | 12.9 (8.06-24.2) | 0 (0-25.8) | 0.658 | 1 |  |
| Ketamine | Decrease | 41.9 (27.4-43.5) | 32.3 (21-41.9) | 1 | 1 | 3.23 (1.61-17.7) | 0 (0-14.5) | 0.643 | 1 | 38.7 (19.4-48.4) | 12.9 (6.45-22.6) | 0.507 | 1 |  |
| Midazolam | Increase | 35.5 (22.6-61.3) | 9.68 (6.45-22.6) | 0.026 | 0.676 | 12.9 (0-45.2) | 6.45 (0-12.9) | 0.642 | 1 | 12.9 (12.9-22.6) | 12.9 (12.9-22.6) | 1 | 1 |  |
| Norepinephrine | Decrease | 45.2 (16.1-71) | 41.9 (16.1-74.2) | 0.858 | 1 | 16.1 (0-41.9) | 12.9 (0-41.9) | 0.519 | 1 | 25.8 (12.9-62.1) | 19.4 (9.68-64.5) | 0.327 | 1 |  |
| Norepinephrine | Increase | 45.2 (13.7-74.2) | 45.2 (19.4-74.2) | 0.652 | 1 | 16.1 (0-50.8) | 16.1 (0-51.6) | 0.828 | 1 | 19.4 (6.45-66.9) | 19.4 (9.68-64.5) | 0.715 | 1 |  |
| Phenylephrine | Increase | 0 (0-33.9) | 0 (0-16.9) | 0.924 | 1 | 0 (0-16.9) | 0 (0-0) | 0.462 | 1 | 12.9 (12.9-12.9) | 12.9 (12.9-12.9) | 0.753 | 1 |  |
| Propofol | Decrease | 25.8 (12.9-54.8) | 38.7 (16.1-61.3) | 0.374 | 1 | 9.68 (0-29) | 8.06 (0-29) | 0.974 | 1 | 12.9 (9.68-41.9) | 12.9 (6.45-48.4) | 0.971 | 1 |  |
| Propofol | Increase | 35.5 (7.26-54.8) | 25.8 (12.9-45.2) | 0.498 | 1 | 8.06 (0-21.8) | 3.23 (0-22.6) | 0.853 | 1 | 12.9 (7.26-24.2) | 12.9 (6.45-35.5) | 0.742 | 1 |  |
| Vasopressin | Decrease | 64.5 (51.6-74.2) | 51.6 (40.3-67.7) | 0.429 | 1 | 29 (6.45-41.9) | 12.9 (1.61-30.6) | 0.55 | 1 | 16.1 (1.61-45.2) | 16.1 (1.61-48.4) | 1 | 1 |  |
| Vasopressin | Increase | 59.7 (19.4-77.4) | 24.2 (21-46.8) | 0.598 | 1 | 6.45 (0-41.9) | 0 (0-39.5) | 0.575 | 1 | 12.9 (5.65-37.1) | 12.9 (4.84-29.8) | 1 | 1 |  |
| **Name** | **Mean Dose Change** | **% time RAC > -0.05** | | | | **% time COx_R_a > 0** | | | | **% time COx_R_a > 0.3** | | | |  |
|  |  | **Pre Dose** | **Post Dose** | **P value** | **Adj P Value** | **Pre Dose** | **Post Dose** | **P value** | **Adj P Value** | **Pre Dose** | **Post Dose** | **P value** | **Adj P Value** |  |
| Fentanyl | Decrease | 12.9 (8.87-37.9) | 12.9 (5.65-24.2) | 1 | 1 | 67.7 (46.8-77.4) | 59.7 (29.8-79.8) | 0.633 | 1 | 25.8 (6.45-40.3) | 19.4 (4.03-44.4) | 0.873 | 1 |  |
| Fentanyl | Increase | 9.68 (8.06-16.1) | 12.9 (3.23-12.9) | 0.829 | 1 | 64.5 (43.5-75.8) | 58.1 (31.5-68.5) | 0.612 | 1 | 29 (14.5-45.2) | 19.4 (0-46) | 0.404 | 1 |  |
| Ketamine | Increase | 12.9 (8.06-21) | 0 (0-24.2) | 0.658 | 1 | 71 (67.7-71) | 61.3 (53.2-62.9) | 0.116 | 1 | 29 (22.6-30.6) | 16.1 (12.9-16.1) | 0.164 | 1 |  |
| Ketamine | Decrease | 35.5 (17.7-41.9) | 0 (0-14.5) | 0.354 | 1 | 77.4 (62.9-88.7) | 48.4 (41.9-69.4) | 0.507 | 1 | 58.1 (29-58.1) | 19.4 (16.1-27.4) | 0.658 | 1 |  |
| Midazolam | Increase | 12.9 (12.9-22.6) | 12.9 (12.9-22.6) | 1 | 1 | 67.7 (58.1-71) | 51.6 (38.7-54.8) | 0.211 | 1 | 41.9 (38.7-51.6) | 16.1 (12.9-29) | 0.153 | 1 |  |
| Norepinephrine | Decrease | 22.6 (12.9-52.4) | 16.1 (6.45-54.8) | 0.228 | 1 | 58.1 (35.5-77.4) | 56.5 (32.3-71) | 0.419 | 1 | 19.4 (6.45-38.7) | 19.4 (3.23-35.5) | 0.199 | 1 |  |
| Norepinephrine | Increase | 16.1 (6.45-58.1) | 16.1 (9.68-61.3) | 0.667 | 1 | 51.6 (29-67.7) | 48.4 (28.2-71) | 0.992 | 1 | 16.1 (3.23-32.3) | 16.1 (3.23-35.5) | 0.976 | 1 |  |
| Phenylephrine | Increase | 12.9 (12.9-12.9) | 12.9 (12.9-12.9) | 0.753 | 1 | 0 (0-21.8) | 0 (0-0) | 0.599 | 1 | 0 (0-2.42) | 0 (0-0) | 0.599 | 1 |  |
| Propofol | Decrease | 12.9 (6.45-35.5) | 12.9 (3.23-38.7) | 0.912 | 1 | 64.5 (45.2-80.6) | 54.8 (32.3-71) | 0.0443 | 1 | 25.8 (12.9-38.7) | 14.5 (3.23-32.3) | 0.0241 | 0.627 |  |
| Propofol | Increase | 9.68 (3.23-19.4) | 12.9 (3.23-29) | 0.676 | 1 | 58.1 (33.9-69.4) | 59.7 (32.3-73.4) | 0.699 | 1 | 16.1 (3.23-40.3) | 12.9 (6.45-37.1) | 0.767 | 1 |  |
| Vasopressin | Decrease | 12.9 (0-40.3) | 12.9 (1.61-38.7) | 0.947 | 1 | 69.4 (42.7-79) | 48.4 (32.3-73.4) | 0.528 | 1 | 24.2 (9.68-51.6) | 24.2 (6.45-45.2) | 0.673 | 1 |  |
| Vasopressin | Increase | 12.9 (2.42-29.8) | 11.3 (2.42-21.8) | 0.873 | 1 | 19.4 (0-74.2) | 38.7 (0-54.8) | 1 | 1 | 3.23 (0-51.6) | 0 (0-9.68) | 0.824 | 1 |  |
| **Name** | **Mean Dose Change** | **% time COx_L_a > 0** | | | | **% time COx_L_a > 0.3** | | | |  | | | |  |
|  |  | **Pre Dose** | **Post Dose** | **P value** | **Adj P Value** | **Pre Dose** | **Post Dose** | **P value** | **Adj P Value** |  |  |  |  |  |
| Fentanyl | Decrease | 64.5 (47.6-78.2) | 54.8 (32.3-68.5) | 0.117 | 1 | 17.7 (6.45-33.1) | 16.1 (6.45-29) | 0.967 | 1 |  |  |  |  |  |
| Fentanyl | Increase | 58.1 (51.6-66.9) | 58.1 (48.4-71) | 0.829 | 1 | 17.7 (7.26-31.5) | 6.45 (1.61-37.1) | 0.472 | 1 |  |  |  |  |  |
| Ketamine | Increase | 74.2 (66.1-75.8) | 74.2 (66.1-80.6) | 1 | 1 | 25.8 (24.2-29) | 16.1 (11.3-17.7) | 0.1 | 1 |  |  |  |  |  |
| Ketamine | Decrease | 87.1 (58.1-93.5) | 67.7 (61.3-80.6) | 1 | 1 | 61.3 (30.6-64.5) | 29 (29-32.3) | 0.658 | 1 |  |  |  |  |  |
| Midazolam | Increase | 71 (19.4-74.2) | 58.1 (41.9-64.5) | 0.18 | 1 | 45.2 (12.9-54.8) | 22.6 (16.1-32.3) | 0.243 | 1 |  |  |  |  |  |
| Norepinephrine | Decrease | 54.8 (31.5-71.8) | 54.8 (29-71) | 0.866 | 1 | 16.1 (3.23-32.3) | 16.1 (3.23-32.3) | 0.708 | 1 |  |  |  |  |  |
| Norepinephrine | Increase | 51.6 (22.6-67.7) | 51.6 (25.8-71) | 0.429 | 1 | 14.5 (0-29) | 16.1 (0-29) | 0.899 | 1 |  |  |  |  |  |
| Phenylephrine | Increase | 0 (0-0) | 0 (0-0) | 1 | 1 | 0 (0-0) | 0 (0-0) | 1 | 1 |  |  |  |  |  |
| Propofol | Decrease | 64.5 (41.9-77.4) | 54.8 (27.4-71) | 0.0835 | 1 | 22.6 (8.87-33.1) | 16.1 (3.23-35.5) | 0.16 | 1 |  |  |  |  |  |
| Propofol | Increase | 59.7 (46-67.7) | 61.3 (45.2-74.2) | 0.507 | 1 | 19.4 (6.45-32.3) | 19.4 (3.23-35.5) | 0.997 | 1 |  |  |  |  |  |
| Vasopressin | Decrease | 58.1 (40.3-71) | 45.2 (36.3-62.1) | 0.48 | 1 | 9.68 (3.23-25.8) | 11.3 (6.45-26.6) | 0.972 | 1 |  |  |  |  |  |
| Vasopressin | Increase | 50 (14.5-58.1) | 51.6 (4.84-65.3) | 0.751 | 1 | 6.45 (0-20.2) | 16.1 (0-33.9) | 0.627 | 1 |  |  |  |  |  |
| **Bolus** | | | | | | | | | | | | | | |
| **Name** | **Doses** | **Mean MAP** | | | | **Mean ICP** | | | | **Mean rSO2_R** | | | |  |
|  |  | **Pre Dose** | **Post Dose** | **P value** | **Adj P Value** | **Pre Dose** | **Post Dose** | **P value** | **Adj P Value** | **Pre Dose** | **Post Dose** | **P value** | **Adj P Value** |  |
| Fentanyl | 23 | 87.9 (76.6-92) | 83.5 (73-90.4) | 0.346 | 1 | 17.2 (12-19.5) | 13.6 (11-17.3) | 0.209 | 1 | 61.5 (57.2-69.2) | 61 (56.9-67.9) | 0.903 | 1 |  |
| Ketamine | 3 | 89.4 (80.5-89.5) | 74.5 (74.4-81.3) | 0.7 | 1 | 8.41 (5.9-9.9) | 8.94 (6.82-9.32) | 1 | 1 | 71.4 (70.7-74.3) | 71 (70.1-72.6) | 0.7 | 1 |  |
| Midazolam | 5 | 75.4 (71.9-79.2) | 79.2 (74.2-83) | 0.753 | 1 | 13.2 (11.8-14.2) | 9.43 (8.07-17.6) | 0.6 | 1 | 56.2 (53.6-57.4) | 58.4 (54.6-60) | 0.346 | 1 |  |
| **Name** | **Mean rSO2_L** | | | | **Mean CPP** | | | | **Mean AMP** | | | |  |  |
|  | **Pre Dose** | **Post Dose** | **P value** | **Adj P Value** | **Pre Dose** | **Post Dose** | **P value** | **Adj P Value** | **Pre Dose** | **Post Dose** | **P value** | **Adj P Value** |  |  |
| Fentanyl | 65.9 (63.3-70.2) | 65.6 (62.5-70.3) | 0.811 | 1 | 66.9 (62.2-74) | 69.4 (58.4-74.3) | 0.812 | 1 | 1.86 (1.36-3.22) | 1.83 (1.14-2.37) | 0.36 | 1 |  |  |
| Ketamine | 68.2 (68.2-73.7) | 68.8 (68.3-72.2) | 1 | 1 | 78 (72.8-79.5) | 69.6 (67.2-74.4) | 0.7 | 1 | 1.48 (1.34-1.5) | 1.5 (1.36-1.53) | 0.7 | 1 |  |  |
| Midazolam | 60.2 (57-60.5) | 60.2 (56-64.3) | 1 | 1 | 61.2 (60.1-64.6) | 66.1 (59.3-67.7) | 0.917 | 1 | 1.91 (1.28-2.08) | 1.28 (1.13-1.6) | 0.463 | 1 |  |  |
| **Name** | **Mean PRx** | | | | **Mean PAx** | | | | **Mean RAC** | | | |  |  |
|  | **Pre Dose** | **Post Dose** | **P value** | **Adj P Value** | **Pre Dose** | **Post Dose** | **P value** | **Adj P Value** | **Pre Dose** | **Post Dose** | **P value** | **Adj P Value** |  |  |
| Fentanyl | -0.0686 (-0.161-0.000423) | -0.0619 (-0.16-0.0284) | 0.708 | 1 | -0.121 (-0.185--0.000263) | -0.0988 (-0.221-0) | 0.991 | 1 | -0.443 (-0.562--0.323) | -0.479 (-0.592--0.134) | 0.847 | 1 |  |  |
| Ketamine | -0.0522 (-0.202--0.0426) | -0.0572 (-0.139--0.0468) | 1 | 1 | -0.22 (-0.275--0.102) | -0.159 (-0.21--0.0592) | 0.7 | 1 | -0.357 (-0.446--0.302) | -0.403 (-0.568--0.259) | 1 | 1 |  |  |
| Midazolam | -0.00479 (-0.0913-0.197) | -0.136 (-0.167--0.00479) | 0.6 | 1 | -0.103 (-0.128-0.165) | -0.141 (-0.194--0.128) | 0.463 | 1 | -0.55 (-0.578--0.428) | -0.42 (-0.428--0.371) | 0.346 | 1 |  |  |
| **Name** | **Mean COx_L_a** | | | | **Mean COx_R_a** | | | | **% time ICP > 20** | | | |  |  |
|  | **Pre Dose** | **Post Dose** | **P value** | **Adj P Value** | **Pre Dose** | **Post Dose** | **P value** | **Adj P Value** | **Pre Dose** | **Post Dose** | **P value** | **Adj P Value** |  |  |
| Fentanyl | 0.0624 (0.00246-0.182) | 0.0599 (0-0.168) | 0.855 | 1 | 0.0919 (0.00329-0.317) | 0.075 (-0.046-0.224) | 0.605 | 1 | 16.1 (0-35.5) | 0 (0-23.4) | 0.134 | 1 |  |  |
| Ketamine | 0.131 (0.0976-0.171) | 0.102 (0.0879-0.122) | 1 | 1 | 0.13 (0.127-0.172) | 0.0717 (0.0231-0.0759) | 0.1 | 1 | 0 (0-0) | 0 (0-0) | 1 | 1 |  |  |
| Midazolam | 0.23 (-0.101-0.237) | 0.133 (0.11-0.134) | 0.463 | 1 | 0.2 (0.184-0.206) | 0.019 (-0.0158-0.119) | 0.463 | 1 | 0 (0-19.4) | 0 (0-19.4) | 1 | 1 |  |  |
| **Name** | **% time ICP > 22** | | | | **% time CPP > 60** | | | | **% time CPP > 70** | | | |  |  |
|  | **Pre Dose** | **Post Dose** | **P value** | **Adj P Value** | **Pre Dose** | **Post Dose** | **P value** | **Adj P Value** | **Pre Dose** | **Post Dose** | **P value** | **Adj P Value** |  |  |
| Fentanyl | 0 (0-22.6) | 0 (0-11.3) | 0.279 | 1 | 12.9 (8.06-15.3) | 12.9 (0-12.9) | 0.159 | 1 | 35.5 (13.7-72.6) | 38.7 (0-71) | 0.576 | 1 |  |  |
| Ketamine | 0 (0-0) | 0 (0-0) | 1 | 1 | 0 (0-0) | 0 (0-0) | 1 | 1 | 96.8 (56.5-98.4) | 38.7 (19.4-56.5) | 0.4 | 1 |  |  |
| Midazolam | 0 (0-3.23) | 0 (0-9.68) | 1 | 1 | 12.9 (12.9-19.4) | 12.9 (12.9-12.9) | 0.797 | 1 | 29 (0-48.4) | 71 (0-83.9) | 0.746 | 1 |  |  |
| **Name** | **% time PRx > 0** | | | | **% time PRx > 0.25** | | | | **% time PRx > 0.35** | | | |  |  |
|  | **Pre Dose** | **Post Dose** | **P value** | **Adj P Value** | **Pre Dose** | **Post Dose** | **P value** | **Adj P Value** | **Pre Dose** | **Post Dose** | **P value** | **Adj P Value** |  |  |
| Fentanyl | 30.6 (19.4-50.8) | 32.3 (11.3-45.2) | 0.847 | 1 | 6.45 (0-21.8) | 0 (0-29) | 0.388 | 1 | 3.23 (0-12.1) | 0 (0-14.5) | 0.294 | 1 |  |  |
| Ketamine | 32.3 (16.1-37.1) | 41.9 (22.6-41.9) | 0.643 | 1 | 3.23 (1.61-6.45) | 9.68 (4.84-9.68) | 0.814 | 1 | 3.23 (1.61-4.84) | 0 (0-1.61) | 0.48 | 1 |  |  |
| Midazolam | 35.5 (35.5-61.3) | 12.9 (9.68-35.5) | 0.596 | 1 | 22.6 (0-45.2) | 6.45 (0-22.6) | 0.914 | 1 | 16.1 (0-41.9) | 6.45 (0-16.1) | 0.914 | 1 |  |  |
| **Name** | **% time PAx > 0** | | | | **% time PAx > 0.25** | | | | **% time RAC > -0.1** | | | |  |  |
|  | **Pre Dose** | **Post Dose** | **P value** | **Adj P Value** | **Pre Dose** | **Post Dose** | **P value** | **Adj P Value** | **Pre Dose** | **Post Dose** | **P value** | **Adj P Value** |  |  |
| Fentanyl | 35.5 (20.2-44.4) | 22.6 (11.3-35.5) | 0.311 | 1 | 4.84 (0-12.9) | 0 (0-4.84) | 0.16 | 1 | 12.9 (9.68-16.1) | 12.9 (4.84-12.9) | 0.746 | 1 |  |  |
| Ketamine | 16.1 (11.3-33.9) | 22.6 (12.9-45.2) | 1 | 1 | 9.68 (6.45-14.5) | 0 (0-16.1) | 0.658 | 1 | 12.9 (8.06-24.2) | 0 (0-25.8) | 0.658 | 1 |  |  |
| Midazolam | 35.5 (22.6-61.3) | 9.68 (6.45-22.6) | 0.249 | 1 | 12.9 (0-45.2) | 6.45 (0-12.9) | 0.914 | 1 | 12.9 (12.9-22.6) | 12.9 (12.9-22.6) | 1 | 1 |  |  |
| **Name** | **% time RAC > -0.05** | | | | **% time COx_R_a > 0** | | | | **% time COx_R_a > 0.3** | | | |  |  |
|  | **Pre Dose** | **Post Dose** | **P value** | **Adj P Value** | **Pre Dose** | **Post Dose** | **P value** | **Adj P Value** | **Pre Dose** | **Post Dose** | **P value** | **Adj P Value** |  |  |
| Fentanyl | 9.68 (7.26-16.1) | 12.9 (3.23-12.9) | 0.817 | 1 | 62.9 (41.1-76.6) | 58.1 (30.6-69.4) | 0.715 | 1 | 27.4 (13.7-48.4) | 22.6 (0-46.8) | 0.491 | 1 |  |  |
| Ketamine | 12.9 (8.06-21) | 0 (0-24.2) | 0.658 | 1 | 71 (67.7-71) | 61.3 (53.2-62.9) | 0.116 | 1 | 29 (22.6-30.6) | 16.1 (12.9-16.1) | 0.164 | 1 |  |  |
| Midazolam | 12.9 (12.9-22.6) | 12.9 (12.9-22.6) | 1 | 1 | 67.7 (58.1-71) | 51.6 (38.7-54.8) | 0.293 | 1 | 41.9 (38.7-51.6) | 16.1 (12.9-29) | 0.293 | 1 |  |  |
| **Name** | **% time COx_L_a > 0** | | | | **% time COx_L_a > 0.3** | | | |  | | | |  |  |
|  | **Pre Dose** | **Post Dose** | **P value** | **Adj P Value** | **Pre Dose** | **Post Dose** | **P value** | **Adj P Value** |  |  |  |  |  |  |
| Fentanyl | 58.1 (51.6-67.7) | 56.5 (48.4-67.7) | 0.942 | 1 | 19.4 (9.68-32.3) | 9.68 (0.806-37.9) | 0.494 | 1 |  |  |  |  |  |  |
| Ketamine | 74.2 (66.1-75.8) | 74.2 (66.1-80.6) | 1 | 1 | 25.8 (24.2-29) | 16.1 (11.3-17.7) | 0.1 | 1 |  |  |  |  |  |  |
| Midazolam | 71 (19.4-74.2) | 58.1 (41.9-64.5) | 0.599 | 1 | 45.2 (12.9-54.8) | 22.6 (16.1-32.3) | 0.673 | 1 |  |  |  |  |  |  |

*The table demonstrates the median and interquartile range of the pre/pose dose windows as well as the Wilcox signed ranked test between these windows with p-values adjusted using the Bonferroni analysis. The “change” indicates if the continuous infusion was increase/decrease or was a bolus dose. AMP, intracranial pulse amplitude; COx_R_a, cerebral oximetry index of right side using MAP; COx_L_a, cerebral oximetry index of left side using MAP; CPP, cerebral prefusion pressure; ICP, intracranial pressure; MAP, mean arterial blood pressure; PAx, pulse amplitude index; PRx, pressure reactivity; RAC, correlation between intracranial pulse amplitude and CPP; rSO2_L, regional oxygen saturation on left side; rSO2_R, regional oxygen saturation on right side.*

# Appendix H. Males

The table contains all infusions given with the patient sex of male, separated into the continuous infusion doses then the bolus doses.

| **Continuous Infusion** | | | | | | | | | | | | | | |
| --- | --- | --- | --- | --- | --- | --- | --- | --- | --- | --- | --- | --- | --- | --- |
| **Name** | **Doses** | **Mean Dose Change** | **Mean MAP** | | | | **Mean ICP** | | | | **Mean rSO2_R** | | | |
|  |  |  | **Pre Dose** | **Post Dose** | **P value** | **Adj P Value** | **Pre Dose** | **Post Dose** | **P value** | **Adj P Value** | **Pre Dose** | **Post Dose** | **P value** | **Adj P Value** |
| Fentanyl | 140 | Decrease | 75.6 (69.9-84.8) | 79.2 (72.1-88.8) | 0.0987 | 1 | 8.67 (5.24-13.3) | 9.17 (5.08-13.7) | 0.793 | 1 | 67.7 (55.4-72) | 66.2 (55.6-72.8) | 0.888 | 1 |
| Fentanyl | 139 | Increase | 77.2 (70-86.6) | 77.2 (69.4-85) | 0.554 | 1 | 11.2 (4.91-14.4) | 10.4 (5.03-14.3) | 0.794 | 1 | 64.8 (52.1-72.3) | 66.7 (54.1-70.6) | 0.601 | 1 |
| Ketamine | 34 | Increase | 77.2 (72.6-88.9) | 77.6 (74.1-89.2) | 0.415 | 1 | 13.4 (7.38-19.2) | 13.8 (7.12-20.3) | 0.975 | 1 | 55.8 (12.9-69.3) | 57 (12.8-69.1) | 0.846 | 1 |
| Ketamine | 10 | Decrease | 76.2 (70.2-81.5) | 78.6 (68.9-82.7) | 0.961 | 1 | 13 (4.07-14.8) | 12.5 (7.42-18.3) | 0.573 | 1 | 67.3 (61.3-69.5) | 68.4 (62.4-70.9) | 0.846 | 1 |
| Midazolam | 74 | Increase | 76 (69.2-86.9) | 77.6 (72.1-87.4) | 0.564 | 1 | 11.3 (8.5-14.6) | 10.7 (6.5-14.7) | 0.223 | 1 | 61.7 (39-68.7) | 61.9 (36.5-69.2) | 0.757 | 1 |
| Midazolam | 73 | Decrease | 79.1 (70.3-97) | 80.8 (70.5-96.3) | 0.768 | 1 | 13.4 (10.3-17.7) | 11.6 (9.51-17.4) | 0.609 | 1 | 55.9 (16.7-67.7) | 55.1 (0-67.2) | 0.803 | 1 |
| Norepinephrine | 1237 | Decrease | 79.1 (72.2-86.8) | 77.8 (71.6-85.6) | 0.0245 | 0.636 | 9.79 (5.79-14) | 10.1 (5.85-14.2) | 0.543 | 1 | 61.9 (53.6-68.9) | 61.5 (53.5-68.9) | 0.786 | 1 |
| Norepinephrine | 994 | Increase | 75.4 (69.4-82.4) | 76 (70-83.1) | 0.241 | 1 | 9.98 (5.75-14.8) | 9.81 (5.79-14.4) | 0.781 | 1 | 61.9 (54.1-69.2) | 62.1 (54-69.4) | 0.918 | 1 |
| Phenylephrine | 11 | Decrease | 71.8 (64-81.5) | 67.5 (62.2-73.3) | 0.347 | 1 | 7.65 (0.879-11.1) | 7.97 (4.66-10.9) | 0.74 | 1 | 71.3 (52.8-79.2) | 71.4 (54.1-80.2) | 0.799 | 1 |
| Phenylephrine | 9 | Increase | 65.1 (59.7-67.5) | 61 (59.9-66.4) | 1 | 1 | 0.618 (-0.698-5.21) | 1.79 (1.17-6.02) | 0.546 | 1 | 79.3 (64.3-83) | 78.1 (60.5-83.4) | 1 | 1 |
| Propofol | 341 | Decrease | 77.7 (69.9-85.4) | 78.3 (70.6-86.9) | 0.241 | 1 | 7.89 (4.31-12.6) | 8.02 (4.23-13.1) | 0.693 | 1 | 64.5 (54.9-71.1) | 64 (55.2-70.8) | 0.902 | 1 |
| Propofol | 252 | Increase | 78.6 (69.4-89.2) | 77.1 (69-87.3) | 0.164 | 1 | 7.66 (3.55-12.5) | 7.58 (4.36-12) | 0.893 | 1 | 66.9 (56.9-71.9) | 66.2 (56-71.9) | 0.59 | 1 |
| Vasopressin | 47 | Decrease | 77.6 (68.8-86.5) | 75.6 (69.6-88) | 1 | 1 | 5.79 (3.99-11.4) | 7.51 (5.21-14.8) | 0.259 | 1 | 54.7 (31.2-65.8) | 57 (38.3-65.7) | 0.778 | 1 |
| Vasopressin | 36 | Increase | 73.1 (68.9-81.9) | 70.6 (68-83.9) | 0.685 | 1 | 11 (6.07-13.7) | 10.8 (5.52-13.1) | 0.444 | 1 | 61.6 (0-69.9) | 58.8 (0-68.2) | 0.773 | 1 |
| **Name** | **Mean Dose Change** | **Mean rSO2_L** | | | | **Mean CPP** | | | | **Mean AMP** | | | |  |
|  |  | **Pre Dose** | **Post Dose** | **P value** | **Adj P Value** | **Pre Dose** | **Post Dose** | **P value** | **Adj P Value** | **Pre Dose** | **Post Dose** | **P value** | **Adj P Value** |  |
| Fentanyl | Decrease | 65.7 (54.7-73.6) | 65.2 (53.9-73.7) | 0.909 | 1 | 67.9 (60.3-75.9) | 69.8 (62.4-77.6) | 0.231 | 1 | 1.64 (1.04-2.29) | 1.76 (1.13-2.32) | 0.496 | 1 |  |
| Fentanyl | Increase | 67.8 (59.5-74) | 67.2 (57.4-73.5) | 0.917 | 1 | 66.9 (62.3-74.5) | 67.2 (60.3-72.8) | 0.487 | 1 | 1.74 (1.14-3.11) | 1.7 (1.03-3.35) | 0.976 | 1 |  |
| Ketamine | Increase | 64.1 (0-65.9) | 64.5 (0-67) | 0.669 | 1 | 64.5 (53.2-80.7) | 65.9 (58.6-76.6) | 0.581 | 1 | 2.58 (1.09-3.99) | 1.36 (1.1-4.91) | 0.915 | 1 |  |
| Ketamine | Decrease | 69.7 (68.8-77.3) | 70.2 (69.2-77.9) | 0.579 | 1 | 67.7 (63.1-74.9) | 66.1 (60.3-71.4) | 0.503 | 1 | 2.09 (1.68-3.08) | 3.23 (1.54-4.76) | 0.621 | 1 |  |
| Midazolam | Increase | 64.1 (59.9-72.3) | 64.6 (60.2-71.9) | 0.974 | 1 | 63.9 (58.5-75.4) | 67.4 (60.2-74.7) | 0.184 | 1 | 2.82 (1.22-4.92) | 2.77 (1.21-4.36) | 0.526 | 1 |  |
| Midazolam | Decrease | 63.3 (58.1-66.2) | 63 (52.8-65.8) | 0.517 | 1 | 69.9 (59.8-79) | 70.6 (59.8-80.2) | 0.84 | 1 | 2.05 (1.03-4.02) | 1.42 (0.987-4.62) | 0.933 | 1 |  |
| Norepinephrine | Decrease | 64.6 (57.1-71.9) | 64.6 (57.1-71.5) | 0.735 | 1 | 69.4 (62-76.4) | 68 (61.2-74.6) | 0.00572 | 0.149 | 1.91 (1.06-3.41) | 1.87 (1.07-3.33) | 0.933 | 1 |  |
| Norepinephrine | Increase | 64 (56.7-71.6) | 64.4 (57-71.8) | 0.827 | 1 | 65.2 (59.7-71.2) | 65.8 (60.1-71.7) | 0.189 | 1 | 2.02 (1.04-3.77) | 2.02 (1.07-3.8) | 0.563 | 1 |  |
| Phenylephrine | Decrease | 69.6 (56.4-81.3) | 61.2 (45.9-84.5) | 0.976 | 1 | 66.4 (63.9-87.7) | 63 (59.3-75.4) | 0.288 | 1 | 0.713 (0.426-0.846) | 0.779 (0.425-1.01) | 0.833 | 1 |  |
| Phenylephrine | Increase | 81.1 (61.2-89.6) | 79.5 (61.3-91) | 0.863 | 1 | 62.2 (59.1-64.5) | 64.9 (54.2-65.9) | 0.796 | 1 | 0.647 (0.312-0.74) | 0.634 (0.348-1) | 0.436 | 1 |  |
| Propofol | Decrease | 66.6 (59.5-74.1) | 67 (58.7-74.4) | 0.858 | 1 | 68.7 (61.9-76.3) | 69.5 (61.3-79.2) | 0.507 | 1 | 1.55 (0.729-2.48) | 1.58 (0.795-2.55) | 0.516 | 1 |  |
| Propofol | Increase | 66 (58.9-73.3) | 66.2 (58-73) | 0.727 | 1 | 71.1 (62.6-79.7) | 69.2 (61-77.2) | 0.0861 | 1 | 1.71 (0.903-2.64) | 1.74 (0.83-2.67) | 0.888 | 1 |  |
| Vasopressin | Decrease | 62.6 (56.6-66.9) | 63 (56.7-65.7) | 0.984 | 1 | 68.9 (61.9-76.7) | 65.3 (61.6-71.8) | 0.559 | 1 | 1.13 (0.588-2.23) | 1.21 (0.7-2.43) | 0.529 | 1 |  |
| Vasopressin | Increase | 65.7 (60.4-72.1) | 65.6 (60.5-71.2) | 0.978 | 1 | 62.7 (57.9-66) | 62.4 (56.9-70.1) | 0.761 | 1 | 1.49 (0.573-3.2) | 1.31 (0.506-3.3) | 0.669 | 1 |  |
| **Name** | **Mean Dose Change** | **Mean PRx** | | | | **Mean PAx** | | | | **Mean RAC** | | | |  |
|  |  | **Pre Dose** | **Post Dose** | **P value** | **Adj P Value** | **Pre Dose** | **Post Dose** | **P value** | **Adj P Value** | **Pre Dose** | **Post Dose** | **P value** | **Adj P Value** |  |
| Fentanyl | Decrease | 0.0915 (-0.046-0.296) | 0.114 (-0.0535-0.328) | 0.876 | 1 | -0.0384 (-0.223-0.162) | -0.0353 (-0.238-0.122) | 0.556 | 1 | -0.28 (-0.486--0.00518) | -0.271 (-0.474--0.0221) | 0.759 | 1 |  |
| Fentanyl | Increase | 0.102 (-0.0875-0.341) | 0.106 (-0.0787-0.263) | 0.534 | 1 | -0.0246 (-0.2-0.145) | -0.0273 (-0.199-0.126) | 0.428 | 1 | -0.241 (-0.43-0.0136) | -0.27 (-0.462--0.0531) | 0.409 | 1 |  |
| Ketamine | Increase | 0.181 (-0.061-0.408) | 0.193 (-0.146-0.296) | 0.506 | 1 | 0.108 (-0.147-0.275) | 0.173 (-0.228-0.283) | 0.91 | 1 | -0.172 (-0.399-0.0448) | -0.19 (-0.473-0.019) | 0.616 | 1 |  |
| Ketamine | Decrease | -0.0583 (-0.0926-0.0598) | -0.0088 (-0.213-0.107) | 1 | 1 | -0.00136 (-0.0681-0.0479) | -0.0894 (-0.184--0.0147) | 0.274 | 1 | -0.296 (-0.384--0.119) | -0.47 (-0.607--0.101) | 0.217 | 1 |  |
| Midazolam | Increase | -0.0153 (-0.114-0.324) | -0.0249 (-0.225-0.238) | 0.184 | 1 | -0.0759 (-0.205-0.144) | -0.166 (-0.271-0.12) | 0.178 | 1 | -0.33 (-0.464--0.144) | -0.352 (-0.482--0.151) | 0.585 | 1 |  |
| Midazolam | Decrease | 0 (-0.127-0.239) | 0 (-0.164-0.236) | 0.849 | 1 | -0.0637 (-0.252-0.0992) | -0.00232 (-0.186-0.115) | 0.329 | 1 | -0.289 (-0.443--0.102) | -0.243 (-0.444--0.0266) | 0.663 | 1 |  |
| Norepinephrine | Decrease | 0.0708 (-0.101-0.279) | 0.0695 (-0.0995-0.268) | 0.765 | 1 | 0 (-0.186-0.195) | -0.000765 (-0.2-0.185) | 0.47 | 1 | -0.246 (-0.467--0.0208) | -0.251 (-0.475--0.0168) | 0.531 | 1 |  |
| Norepinephrine | Increase | 0.0607 (-0.13-0.277) | 0.0283 (-0.133-0.256) | 0.291 | 1 | -0.0313 (-0.209-0.185) | -0.0277 (-0.201-0.191) | 0.644 | 1 | -0.27 (-0.487--0.00534) | -0.266 (-0.484--0.0151) | 0.795 | 1 |  |
| Phenylephrine | Decrease | 0.264 (0.145-0.428) | 0.11 (0.00189-0.301) | 0.0512 | 1 | -0.137 (-0.273--0.0504) | -0.104 (-0.347-0.187) | 0.928 | 1 | -0.163 (-0.319--0.0128) | -0.174 (-0.346-0.174) | 0.928 | 1 |  |
| Phenylephrine | Increase | 0.144 (0.106-0.317) | 0.267 (0.132-0.288) | 0.73 | 1 | -0.226 (-0.42--0.0777) | -0.149 (-0.176--0.072) | 0.436 | 1 | -0.249 (-0.474--0.0972) | -0.155 (-0.227--0.14) | 0.436 | 1 |  |
| Propofol | Decrease | 0.161 (0-0.336) | 0.16 (0-0.332) | 0.857 | 1 | 0 (-0.149-0.187) | 0.00674 (-0.156-0.207) | 0.953 | 1 | -0.2 (-0.409-0) | -0.204 (-0.396-0) | 0.878 | 1 |  |
| Propofol | Increase | 0.156 (0-0.336) | 0.194 (0-0.345) | 0.34 | 1 | 0 (-0.162-0.206) | 0.013 (-0.144-0.228) | 0.322 | 1 | -0.213 (-0.41-0) | -0.169 (-0.392-0) | 0.251 | 1 |  |
| Vasopressin | Decrease | 0.0546 (-0.109-0.272) | 0.125 (-0.0294-0.299) | 0.468 | 1 | -0.0488 (-0.239-0.133) | -0.0217 (-0.232-0.0984) | 0.463 | 1 | -0.32 (-0.467--0.0952) | -0.248 (-0.475--0.0971) | 0.896 | 1 |  |
| Vasopressin | Increase | 0.0868 (-0.0286-0.447) | 0.184 (0-0.349) | 0.66 | 1 | -0.0178 (-0.129-0.163) | -0.00879 (-0.146-0.14) | 0.677 | 1 | -0.22 (-0.439--0.0243) | -0.165 (-0.346--0.0251) | 0.492 | 1 |  |
| **Name** | **Mean Dose Change** | **Mean COx_L_a** | | | | **Mean COx_R_a** | | | | **% time ICP > 20** | | | |  |
|  |  | **Pre Dose** | **Post Dose** | **P value** | **Adj P Value** | **Pre Dose** | **Post Dose** | **P value** | **Adj P Value** | **Pre Dose** | **Post Dose** | **P value** | **Adj P Value** |  |
| Fentanyl | Decrease | 0.0648 (-0.04-0.177) | 0.0688 (-0.00787-0.174) | 0.823 | 1 | 0.0814 (0-0.17) | 0.0339 (-0.0339-0.189) | 0.312 | 1 | 0 (0-0) | 0 (0-0) | 0.702 | 1 |  |
| Fentanyl | Increase | 0.0709 (0-0.204) | 0.0443 (-0.0344-0.172) | 0.204 | 1 | 0.0931 (0-0.181) | 0.0292 (-0.00972-0.206) | 0.193 | 1 | 0 (0-6.45) | 0 (0-3.23) | 0.548 | 1 |  |
| Ketamine | Increase | 0 (-0.0203-0.115) | 0.00896 (0-0.195) | 0.296 | 1 | 0.00286 (0-0.0999) | 0 (0-0.18) | 0.928 | 1 | 0 (0-29) | 4.84 (0-65.3) | 0.349 | 1 |  |
| Ketamine | Decrease | 0.174 (0.0161-0.27) | 0.09 (0-0.168) | 0.627 | 1 | 0.0275 (-0.00336-0.283) | 0.087 (0-0.236) | 0.6 | 1 | 0 (0-6.45) | 17.7 (2.42-38.7) | 0.0938 | 1 |  |
| Midazolam | Increase | 0.0629 (-0.001-0.136) | 0.0136 (-0.0409-0.205) | 0.496 | 1 | 0.0708 (-0.0236-0.172) | 0.017 (-0.0609-0.142) | 0.162 | 1 | 0 (0-2.42) | 0 (0-0) | 0.358 | 1 |  |
| Midazolam | Decrease | 0 (0-0.17) | 0.0101 (0-0.118) | 0.973 | 1 | 0.031 (-0.0579-0.104) | 0.0215 (-0.0616-0.15) | 0.508 | 1 | 0 (0-19.4) | 0 (0-6.45) | 0.759 | 1 |  |
| Norepinephrine | Decrease | 0.0437 (-0.0179-0.16) | 0.0367 (-0.0131-0.158) | 0.907 | 1 | 0.0432 (-0.0197-0.163) | 0.0366 (-0.0249-0.155) | 0.673 | 1 | 0 (0-0) | 0 (0-0) | 0.805 | 1 |  |
| Norepinephrine | Increase | 0.0413 (-0.0199-0.165) | 0.0329 (-0.0373-0.166) | 0.225 | 1 | 0.0443 (-0.00491-0.169) | 0.0298 (-0.0246-0.156) | 0.133 | 1 | 0 (0-3.23) | 0 (0-0) | 0.174 | 1 |  |
| Phenylephrine | Decrease | 0.115 (-0.0628-0.254) | 0.0991 (-0.0344-0.174) | 0.689 | 1 | 0.154 (-0.0274-0.337) | 0.0468 (-0.0347-0.221) | 0.833 | 1 | 0 (0-0) | 0 (0-0) | 1 | 1 |  |
| Phenylephrine | Increase | 0.0577 (0.0339-0.0971) | 0.174 (-0.00941-0.239) | 0.222 | 1 | 0.0119 (-0.0694-0.0468) | 0.0287 (0-0.101) | 0.666 | 1 | 0 (0-0) | 0 (0-0) | 1 | 1 |  |
| Propofol | Decrease | 0.00827 (-0.0546-0.162) | 0.0384 (-0.0462-0.18) | 0.25 | 1 | 0.02 (-0.0524-0.133) | 0.0383 (-0.0354-0.173) | 0.197 | 1 | 0 (0-0) | 0 (0-0) | 0.264 | 1 |  |
| Propofol | Increase | 0.0385 (-0.00747-0.175) | 0.0204 (-0.0311-0.163) | 0.243 | 1 | 0.0332 (-0.0384-0.156) | 0 (-0.0681-0.15) | 0.338 | 1 | 0 (0-0) | 0 (0-0) | 0.222 | 1 |  |
| Vasopressin | Decrease | 0.0195 (0-0.17) | 0 (-0.00447-0.0843) | 0.268 | 1 | 0.045 (-0.058-0.16) | 0.0871 (-0.0897-0.195) | 0.667 | 1 | 0 (0-0) | 0 (0-0) | 0.23 | 1 |  |
| Vasopressin | Increase | 4.35e-05 (0-0.0882) | 0.035 (0-0.134) | 0.634 | 1 | 0.0439 (-0.0325-0.124) | 0.047 (-0.0161-0.141) | 0.762 | 1 | 0 (0-1.61) | 0 (0-0) | 0.0659 | 1 |  |
| **Name** | **Mean Dose Change** | **% time ICP > 22** | | | | **% time CPP > 60** | | | | **% time CPP > 70** | | | |  |
|  |  | **Pre Dose** | **Post Dose** | **P value** | **Adj P Value** | **Pre Dose** | **Post Dose** | **P value** | **Adj P Value** | **Pre Dose** | **Post Dose** | **P value** | **Adj P Value** |  |
| Fentanyl | Decrease | 0 (0-0) | 0 (0-0) | 0.515 | 1 | 6.45 (0-12.9) | 4.84 (0-12.9) | 0.993 | 1 | 41.9 (3.23-87.1) | 54.8 (5.65-90.3) | 0.24 | 1 |  |
| Fentanyl | Increase | 0 (0-0) | 0 (0-0) | 0.767 | 1 | 9.68 (0-12.9) | 9.68 (0-12.9) | 0.969 | 1 | 48.4 (6.45-87.1) | 41.9 (3.23-87.1) | 0.835 | 1 |  |
| Ketamine | Increase | 0 (0-6.45) | 0 (0-3.23) | 0.883 | 1 | 12.9 (6.45-54.8) | 12.9 (0-12.9) | 0.332 | 1 | 83.9 (0-90.3) | 62.9 (0-87.1) | 0.793 | 1 |  |
| Ketamine | Decrease | 0 (0-0) | 4.84 (0-15.3) | 0.0339 | 0.883 | 12.9 (0-12.9) | 12.9 (3.23-21.8) | 0.369 | 1 | 87.1 (19.4-91.9) | 54.8 (9.68-57.3) | 0.394 | 1 |  |
| Midazolam | Increase | 0 (0-0) | 0 (0-0) | 0.211 | 1 | 12.9 (0-16.1) | 12.9 (0-12.9) | 0.194 | 1 | 33.9 (3.23-87.1) | 75.8 (6.45-90.3) | 0.243 | 1 |  |
| Midazolam | Decrease | 0 (0-3.23) | 0 (0-0) | 0.285 | 1 | 12.9 (0-12.9) | 12.9 (0-12.9) | 0.884 | 1 | 80.6 (0-96.8) | 80.6 (9.68-93.5) | 0.918 | 1 |  |
| Norepinephrine | Decrease | 0 (0-0) | 0 (0-0) | 0.445 | 1 | 9.68 (0-12.9) | 9.68 (0-12.9) | 0.154 | 1 | 67.7 (12.9-90.3) | 56.5 (6.45-87.1) | 0.000908 | 0.0236 |  |
| Norepinephrine | Increase | 0 (0-0) | 0 (0-0) | 0.245 | 1 | 12.9 (0-16.1) | 12.9 (0-12.9) | 0.00496 | 0.129 | 32.3 (0-77.4) | 38.7 (0-83.9) | 0.0985 | 1 |  |
| Phenylephrine | Decrease | 0 (0-0) | 0 (0-0) | 1 | 1 | 9.68 (0-21.8) | 12.9 (8.06-30.6) | 0.397 | 1 | 54.8 (4.84-87.1) | 0 (0-87.1) | 0.35 | 1 |  |
| Phenylephrine | Increase | 0 (0-0) | 0 (0-0) | 1 | 1 | 29 (12.9-96.8) | 19.4 (12.9-87.1) | 0.654 | 1 | 0 (0-87.1) | 6.45 (0-41.9) | 0.697 | 1 |  |
| Propofol | Decrease | 0 (0-0) | 0 (0-0) | 0.494 | 1 | 9.68 (0-12.9) | 9.68 (0-12.9) | 0.838 | 1 | 54.8 (3.23-87.1) | 61.3 (3.23-87.1) | 0.309 | 1 |  |
| Propofol | Increase | 0 (0-0) | 0 (0-0) | 0.53 | 1 | 9.68 (0-12.9) | 12.9 (0-12.9) | 0.313 | 1 | 77.4 (9.68-87.1) | 59.7 (6.45-87.1) | 0.196 | 1 |  |
| Vasopressin | Decrease | 0 (0-0) | 0 (0-0) | 0.204 | 1 | 12.9 (0-12.9) | 12.9 (0-12.9) | 0.326 | 1 | 75.8 (8.87-87.1) | 64.5 (4.84-87.1) | 0.468 | 1 |  |
| Vasopressin | Increase | 0 (0-0) | 0 (0-0) | 0.0573 | 1 | 12.9 (10.5-16.9) | 12.9 (0-12.9) | 0.205 | 1 | 14.5 (0-75.8) | 19.4 (0-87.1) | 0.728 | 1 |  |
| **Name** | **Mean Dose Change** | **% time PRx > 0** | | | | **% time PRx > 0.25** | | | | **% time PRx > 0.35** | | | |  |
|  |  | **Pre Dose** | **Post Dose** | **P value** | **Adj P Value** | **Pre Dose** | **Post Dose** | **P value** | **Adj P Value** | **Pre Dose** | **Post Dose** | **P value** | **Adj P Value** |  |
| Fentanyl | Decrease | 58.1 (33.1-80.6) | 58.1 (35.5-80.6) | 0.786 | 1 | 29 (9.68-54.8) | 29 (9.68-58.1) | 0.543 | 1 | 22.6 (3.23-41.9) | 22.6 (3.23-45.2) | 0.689 | 1 |  |
| Fentanyl | Increase | 58.1 (29-80.6) | 51.6 (29-82.3) | 0.917 | 1 | 32.3 (6.45-61.3) | 25.8 (9.68-48.4) | 0.886 | 1 | 22.6 (3.23-48.4) | 16.1 (3.23-43.5) | 0.703 | 1 |  |
| Ketamine | Increase | 71 (29-87.1) | 64.5 (23.4-83.9) | 0.614 | 1 | 38.7 (12.9-74.2) | 38.7 (0.806-61.3) | 0.496 | 1 | 32.3 (6.45-61.3) | 22.6 (0-57.3) | 0.648 | 1 |  |
| Ketamine | Decrease | 29 (16.1-56.5) | 29 (17.7-59.7) | 0.972 | 1 | 3.23 (3.23-19.4) | 19.4 (8.06-28.2) | 0.271 | 1 | 3.23 (1.61-14.5) | 14.5 (4.84-24.2) | 0.282 | 1 |  |
| Midazolam | Increase | 48.4 (26.6-76.6) | 41.9 (12.9-77.4) | 0.325 | 1 | 16.1 (3.23-58.1) | 12.9 (0-50.8) | 0.32 | 1 | 6.45 (0-50) | 8.06 (0-34.7) | 0.788 | 1 |  |
| Midazolam | Decrease | 41.9 (12.9-74.2) | 45.2 (12.9-67.7) | 0.829 | 1 | 12.9 (0-51.6) | 16.1 (0-45.2) | 0.909 | 1 | 6.45 (0-38.7) | 9.68 (0-32.3) | 0.596 | 1 |  |
| Norepinephrine | Decrease | 54.8 (29-80.6) | 54.8 (29-80.6) | 0.732 | 1 | 25.8 (6.45-54.8) | 25.8 (6.45-54.8) | 0.853 | 1 | 16.1 (0-41.9) | 16.1 (0-41.9) | 0.997 | 1 |  |
| Norepinephrine | Increase | 51.6 (25.8-77.4) | 48.4 (22.6-77.4) | 0.46 | 1 | 25.8 (3.23-54.8) | 22.6 (3.23-50.8) | 0.105 | 1 | 16.1 (0-41.9) | 12.9 (0-41.1) | 0.059 | 1 |  |
| Phenylephrine | Decrease | 71 (61.3-90.3) | 64.5 (45.2-77.4) | 0.165 | 1 | 51.6 (31.5-65.3) | 22.6 (14.5-62.9) | 0.09 | 1 | 37.1 (21.8-47.6) | 9.68 (4.84-56.5) | 0.206 | 1 |  |
| Phenylephrine | Increase | 71 (64.5-90.3) | 77.4 (64.5-90.3) | 1 | 1 | 38.7 (22.6-58.1) | 58.1 (35.5-71) | 0.352 | 1 | 12.9 (6.45-35.5) | 41.9 (25.8-48.4) | 0.157 | 1 |  |
| Propofol | Decrease | 64.5 (41.9-87.1) | 67.7 (38.7-87.1) | 0.944 | 1 | 35.5 (12.9-61.3) | 38.7 (9.68-64.5) | 0.884 | 1 | 25.8 (3.23-51.6) | 25.8 (3.23-51.6) | 0.744 | 1 |  |
| Propofol | Increase | 64.5 (38.7-85.5) | 71 (45.2-87.1) | 0.19 | 1 | 38.7 (12.9-64.5) | 38.7 (19.4-64.5) | 0.26 | 1 | 25.8 (3.23-51.6) | 29 (9.68-58.1) | 0.183 | 1 |  |
| Vasopressin | Decrease | 61.3 (22.6-83.9) | 58.1 (29-87.1) | 0.668 | 1 | 25.8 (3.23-61.3) | 32.3 (3.23-59.7) | 0.651 | 1 | 17.7 (0-45.2) | 19.4 (0-43.5) | 0.889 | 1 |  |
| Vasopressin | Increase | 59.7 (35.5-82.3) | 72.6 (46.8-87.1) | 0.281 | 1 | 32.3 (12.9-66.9) | 41.9 (15.3-62.1) | 0.705 | 1 | 16.1 (5.65-66.1) | 27.4 (6.45-50) | 0.739 | 1 |  |
| **Name** | **Mean Dose Change** | **% time PAx > 0** | | | | **% time PAx > 0.25** | | | | **% time RAC > -0.1** | | | |  |
|  |  | **Pre Dose** | **Post Dose** | **P value** | **Adj P Value** | **Pre Dose** | **Post Dose** | **P value** | **Adj P Value** | **Pre Dose** | **Post Dose** | **P value** | **Adj P Value** |  |
| Fentanyl | Decrease | 38.7 (13.7-67.7) | 35.5 (16.1-64.5) | 0.591 | 1 | 14.5 (0-35.5) | 12.9 (0-32.3) | 0.425 | 1 | 25.8 (12.9-57.3) | 25.8 (12.9-58.1) | 0.729 | 1 |  |
| Fentanyl | Increase | 38.7 (19.4-74.2) | 38.7 (19.4-64.5) | 0.703 | 1 | 16.1 (0-32.3) | 12.9 (0-35.5) | 0.613 | 1 | 29 (12.9-67.7) | 25.8 (12.9-50) | 0.852 | 1 |  |
| Ketamine | Increase | 67.7 (9.68-80.6) | 59.7 (12.9-83.1) | 0.99 | 1 | 35.5 (3.23-58.1) | 25.8 (3.23-58.1) | 0.859 | 1 | 38.7 (12.9-58.1) | 30.6 (12.9-58.1) | 0.631 | 1 |  |
| Ketamine | Decrease | 35.5 (24.2-58.1) | 33.9 (21-38.7) | 0.437 | 1 | 12.9 (3.23-19.4) | 12.9 (9.68-20.2) | 0.83 | 1 | 25.8 (9.68-32.3) | 11.3 (3.23-16.1) | 0.396 | 1 |  |
| Midazolam | Increase | 32.3 (12.9-64.5) | 29 (8.06-63.7) | 0.408 | 1 | 12.9 (0-37.9) | 8.06 (0-29) | 0.264 | 1 | 16.1 (12.9-48.4) | 16.1 (12.9-45.2) | 0.748 | 1 |  |
| Midazolam | Decrease | 29 (6.45-64.5) | 45.2 (12.9-64.5) | 0.236 | 1 | 9.68 (0-32.3) | 16.1 (0-29) | 0.648 | 1 | 16.1 (12.9-48.4) | 25.8 (12.9-54.8) | 0.26 | 1 |  |
| Norepinephrine | Decrease | 45.2 (19.4-71) | 45.2 (16.1-71) | 0.591 | 1 | 16.1 (0-41.9) | 16.1 (0-41.9) | 0.642 | 1 | 25.8 (12.9-58.1) | 25.8 (12.9-58.1) | 0.462 | 1 |  |
| Norepinephrine | Increase | 38.7 (16.1-71) | 38.7 (16.1-67.7) | 0.946 | 1 | 12.9 (0-41.9) | 14.5 (0-41.9) | 0.835 | 1 | 25.8 (12.9-54.8) | 25.8 (12.9-54.8) | 0.998 | 1 |  |
| Phenylephrine | Decrease | 32.3 (11.3-49.2) | 12.9 (0-59.7) | 0.577 | 1 | 11.3 (0-19.4) | 0 (0-32.3) | 0.445 | 1 | 35.5 (17.7-71) | 25.8 (6.45-61.3) | 0.478 | 1 |  |
| Phenylephrine | Increase | 19.4 (0-29) | 35.5 (25.8-45.2) | 0.144 | 1 | 0 (0-0) | 6.45 (0-9.68) | 0.089 | 1 | 29 (3.23-45.2) | 48.4 (32.3-54.8) | 0.171 | 1 |  |
| Propofol | Decrease | 48.4 (22.6-71) | 48.4 (19.4-74.2) | 0.82 | 1 | 16.1 (3.23-38.7) | 16.1 (3.23-41.9) | 0.94 | 1 | 29 (12.9-61.3) | 29 (12.9-61.3) | 0.754 | 1 |  |
| Propofol | Increase | 48.4 (17.7-71) | 48.4 (22.6-74.2) | 0.28 | 1 | 16.1 (0-45.2) | 19.4 (3.23-48.4) | 0.519 | 1 | 29 (12.9-58.1) | 32.3 (12.9-64.5) | 0.154 | 1 |  |
| Vasopressin | Decrease | 33.9 (8.87-60.5) | 32.3 (16.1-58.1) | 0.518 | 1 | 4.84 (0-29) | 6.45 (0-29) | 0.469 | 1 | 16.1 (11.3-42.7) | 25.8 (12.9-45.2) | 0.377 | 1 |  |
| Vasopressin | Increase | 40.3 (14.5-68.5) | 37.1 (15.3-67.7) | 0.693 | 1 | 12.9 (2.42-39.5) | 9.68 (0-29.8) | 0.34 | 1 | 24.2 (12.9-50) | 33.9 (12.9-57.3) | 0.752 | 1 |  |
| **Name** | **Mean Dose Change** | **% time RAC > -0.05** | | | | **% time COx_R_a > 0** | | | | **% time COx_R_a > 0.3** | | | |  |
|  |  | **Pre Dose** | **Post Dose** | **P value** | **Adj P Value** | **Pre Dose** | **Post Dose** | **P value** | **Adj P Value** | **Pre Dose** | **Post Dose** | **P value** | **Adj P Value** |  |
| Fentanyl | Decrease | 22.6 (12.9-54) | 19.4 (9.68-48.4) | 0.401 | 1 | 51.6 (29-69.4) | 48.4 (22.6-71) | 0.718 | 1 | 19.4 (6.45-35.5) | 16.1 (0-29.8) | 0.276 | 1 |  |
| Fentanyl | Increase | 25.8 (12.9-61.3) | 22.6 (12.9-43.5) | 0.657 | 1 | 54.8 (32.3-74.2) | 48.4 (29-74.2) | 0.683 | 1 | 22.6 (6.45-38.7) | 19.4 (3.23-38.7) | 0.417 | 1 |  |
| Ketamine | Increase | 29 (12.9-58.1) | 25.8 (12.9-53.2) | 0.573 | 1 | 45.2 (0-61.3) | 38.7 (0-63.7) | 0.804 | 1 | 14.5 (0-25) | 6.45 (0-33.1) | 0.969 | 1 |  |
| Ketamine | Decrease | 22.6 (6.45-29) | 11.3 (3.23-16.1) | 0.571 | 1 | 54.8 (32.3-74.2) | 61.3 (45.2-69.4) | 0.902 | 1 | 21 (7.26-38.7) | 25.8 (11.3-46.8) | 0.62 | 1 |  |
| Midazolam | Increase | 16.1 (9.68-44.4) | 16.1 (12.9-41.1) | 0.969 | 1 | 48.4 (32.3-66.9) | 46.8 (22.6-67.7) | 0.266 | 1 | 17.7 (3.23-34.7) | 9.68 (0.806-29) | 0.124 | 1 |  |
| Midazolam | Decrease | 12.9 (12.9-38.7) | 16.1 (12.9-45.2) | 0.201 | 1 | 45.2 (19.4-61.3) | 41.9 (25.8-61.3) | 0.691 | 1 | 9.68 (0-29) | 12.9 (0-32.3) | 0.234 | 1 |  |
| Norepinephrine | Decrease | 22.6 (12.9-51.6) | 22.6 (12.9-51.6) | 0.522 | 1 | 48.4 (25.8-67.7) | 48.4 (25.8-67.7) | 0.691 | 1 | 12.9 (0-32.3) | 12.9 (0-29.8) | 0.969 | 1 |  |
| Norepinephrine | Increase | 22.6 (12.9-51.6) | 22.6 (12.9-51.6) | 0.872 | 1 | 51.6 (25.8-67.7) | 48.4 (22.6-65.3) | 0.203 | 1 | 16.1 (0-32.3) | 12.9 (0-32.3) | 0.0717 | 1 |  |
| Phenylephrine | Decrease | 30.6 (12.9-63.7) | 22.6 (6.45-59.7) | 0.496 | 1 | 38.7 (19.4-66.1) | 48.4 (29-66.1) | 0.951 | 1 | 30.6 (9.68-53.2) | 19.4 (1.61-43.5) | 0.597 | 1 |  |
| Phenylephrine | Increase | 16.1 (0-45.2) | 38.7 (29-41.9) | 0.198 | 1 | 32.3 (29-48.4) | 16.1 (16.1-48.4) | 0.328 | 1 | 0 (0-19.4) | 0 (0-19.4) | 0.884 | 1 |  |
| Propofol | Decrease | 22.6 (12.9-54.8) | 22.6 (12.9-54.8) | 0.928 | 1 | 45.2 (22.6-64.5) | 48.4 (26.6-64.5) | 0.312 | 1 | 12.9 (0-32.3) | 16.1 (0.806-35.5) | 0.26 | 1 |  |
| Propofol | Increase | 25.8 (12.9-54.8) | 29 (12.9-55.6) | 0.19 | 1 | 48.4 (29-64.5) | 41.9 (22.6-64.5) | 0.462 | 1 | 16.1 (3.23-32.3) | 12.9 (0-32.3) | 0.567 | 1 |  |
| Vasopressin | Decrease | 12.9 (8.87-32.3) | 19.4 (12.9-35.5) | 0.283 | 1 | 51.6 (25.8-71.8) | 54.8 (29-68.5) | 0.936 | 1 | 9.68 (0-30.6) | 16.1 (0-29.8) | 0.53 | 1 |  |
| Vasopressin | Increase | 17.7 (12.9-47.6) | 22.6 (12.9-50) | 0.906 | 1 | 48.4 (32.3-61.3) | 51.6 (32.3-74.2) | 0.515 | 1 | 12.9 (3.23-25.8) | 12.9 (0-25.8) | 0.963 | 1 |  |
| **Name** | **Mean Dose Change** | **% time COx_L_a > 0** | | | | **% time COx_L_a > 0.3** | | | |  | | | |  |
|  |  | **Pre Dose** | **Post Dose** | **P value** | **Adj P Value** | **Pre Dose** | **Post Dose** | **P value** | **Adj P Value** |  |  |  |  |  |
| Fentanyl | Decrease | 48.4 (32.3-71) | 54.8 (35.5-67.7) | 0.712 | 1 | 19.4 (6.45-35.5) | 19.4 (3.23-32.3) | 0.633 | 1 |  |  |  |  |  |
| Fentanyl | Increase | 51.6 (32.3-69.4) | 48.4 (25.8-71) | 0.577 | 1 | 22.6 (6.45-37.1) | 19.4 (1.61-37.1) | 0.379 | 1 |  |  |  |  |  |
| Ketamine | Increase | 35.5 (0-54.8) | 33.9 (3.23-58.1) | 0.917 | 1 | 9.68 (0-32.3) | 6.45 (0-34.7) | 0.699 | 1 |  |  |  |  |  |
| Ketamine | Decrease | 62.9 (35.5-71) | 48.4 (41.9-71) | 0.715 | 1 | 35.5 (15.3-47.6) | 22.6 (16.1-32.3) | 0.421 | 1 |  |  |  |  |  |
| Midazolam | Increase | 45.2 (25.8-64.5) | 38.7 (19.4-77.4) | 0.763 | 1 | 22.6 (6.45-32.3) | 12.9 (0-32.3) | 0.231 | 1 |  |  |  |  |  |
| Midazolam | Decrease | 25.8 (0-61.3) | 41.9 (0-54.8) | 0.939 | 1 | 9.68 (0-32.3) | 12.9 (0-27.4) | 0.687 | 1 |  |  |  |  |  |
| Norepinephrine | Decrease | 45.2 (25.8-67.7) | 45.2 (23.4-64.5) | 0.653 | 1 | 12.9 (0-29) | 12.9 (0-29) | 0.682 | 1 |  |  |  |  |  |
| Norepinephrine | Increase | 48.4 (25.8-67.7) | 45.2 (22.6-66.9) | 0.227 | 1 | 16.1 (3.23-32.3) | 12.9 (0-32.3) | 0.346 | 1 |  |  |  |  |  |
| Phenylephrine | Decrease | 46.8 (31.5-64.5) | 29 (19.4-48.4) | 0.294 | 1 | 32.3 (15.3-37.9) | 16.1 (1.61-25.8) | 0.214 | 1 |  |  |  |  |  |
| Phenylephrine | Increase | 25.8 (16.1-45.2) | 58.1 (32.3-74.2) | 0.0769 | 1 | 6.45 (3.23-16.1) | 32.3 (3.23-35.5) | 0.197 | 1 |  |  |  |  |  |
| Propofol | Decrease | 45.2 (22.6-64.5) | 48.4 (25.8-71) | 0.228 | 1 | 12.9 (0-32.3) | 16.1 (0-33.1) | 0.495 | 1 |  |  |  |  |  |
| Propofol | Increase | 51.6 (25.8-71) | 48.4 (25.8-64.5) | 0.234 | 1 | 12.9 (3.23-32.3) | 12.9 (0-32.3) | 0.323 | 1 |  |  |  |  |  |
| Vasopressin | Decrease | 45.2 (0-71) | 32.3 (0-61.3) | 0.457 | 1 | 16.1 (0-22.6) | 6.45 (0-29) | 0.774 | 1 |  |  |  |  |  |
| Vasopressin | Increase | 40.3 (0-64.5) | 48.4 (0-72.6) | 0.779 | 1 | 11.3 (0-26.6) | 12.9 (0-25.8) | 0.729 | 1 |  |  |  |  |  |
| **Bolus** | | | | | | | | | | | | | | |
| **Name** | **Doses** | **Mean MAP** | | | | **Mean ICP** | | | | **Mean rSO2_R** | | | |  |
|  |  | **Pre Dose** | **Post Dose** | **P value** | **Adj P Value** | **Pre Dose** | **Post Dose** | **P value** | **Adj P Value** | **Pre Dose** | **Post Dose** | **P value** | **Adj P Value** |  |
| Fentanyl | 144 | 80.4 (72.3-88.8) | 78.6 (72.3-88.5) | 0.534 | 1 | 11.2 (5.5-15.8) | 10.9 (5.77-14.7) | 0.94 | 1 | 66.7 (56.7-72.9) | 67.4 (57.1-73.2) | 0.717 | 1 |  |
| Ketamine | 10 | 77.8 (73-89.8) | 81.8 (77.8-95.8) | 0.438 | 1 | 20.8 (16.3-21.8) | 20.2 (17.2-21) | 0.838 | 1 | 0 (0-67.7) | 0 (0-66.4) | 1 | 1 |  |
| Midazolam | 38 | 73.5 (69.2-89.2) | 78.7 (72.1-88.8) | 0.54 | 1 | 11.5 (8.5-16.7) | 10.9 (6.97-14.9) | 0.319 | 1 | 61.4 (48.5-67.6) | 59.3 (40.7-67.3) | 0.795 | 1 |  |
| Propofol | 5 | 83.7 (66.6-89.6) | 83.7 (72.4-84.7) | 1 | 1 | 11 (10.5-14) | 10.4 (9.2-11) | 0.6 | 1 | 70.6 (62.3-70.6) | 70.6 (62.8-71.9) | 1 | 1 |  |
| **Name** | **Mean rSO2_L** | | | | **Mean CPP** | | | | **Mean AMP** | | | |  |  |
|  | **Pre Dose** | **Post Dose** | **P value** | **Adj P Value** | **Pre Dose** | **Post Dose** | **P value** | **Adj P Value** | **Pre Dose** | **Post Dose** | **P value** | **Adj P Value** |  |  |
| Fentanyl | 67.9 (60.7-73.8) | 67.6 (60.2-73.7) | 0.903 | 1 | 69.3 (63-76.7) | 68.4 (62.1-75.8) | 0.357 | 1 | 1.93 (1.28-3.3) | 2.02 (1.3-3.66) | 0.781 | 1 |  |  |
| Ketamine | 0 (0-66) | 0 (0-65.7) | 0.809 | 1 | 58.1 (54.8-82.4) | 65.9 (58.6-78.7) | 0.595 | 1 | 1.06 (0.994-2.96) | 1.12 (0.969-4.46) | 0.713 | 1 |  |  |
| Midazolam | 62.8 (58.6-68) | 63.6 (58.3-67.4) | 0.963 | 1 | 63.9 (58.7-75.9) | 67.9 (62.1-76) | 0.24 | 1 | 2.82 (1.2-5) | 2.49 (1.17-4.36) | 0.663 | 1 |  |  |
| Propofol | 66.1 (64.9-72.8) | 66 (64.9-72.7) | 1 | 1 | 63.3 (60.1-78.6) | 61.5 (60.1-74.3) | 0.917 | 1 | 2.07 (0.491-3.85) | 1.72 (0.452-4.26) | 1 | 1 |  |  |
| **Name** | **Mean PRx** | | | | **Mean PAx** | | | | **Mean RAC** | | | |  |  |
|  | **Pre Dose** | **Post Dose** | **P value** | **Adj P Value** | **Pre Dose** | **Post Dose** | **P value** | **Adj P Value** | **Pre Dose** | **Post Dose** | **P value** | **Adj P Value** |  |  |
| Fentanyl | 0.0776 (-0.094-0.322) | 0.085 (-0.0879-0.293) | 0.693 | 1 | -0.0479 (-0.207-0.184) | -0.0393 (-0.241-0.142) | 0.756 | 1 | -0.275 (-0.47--0.0382) | -0.298 (-0.486--0.0771) | 0.849 | 1 |  |  |
| Ketamine | 0.299 (-0.0173-0.413) | 0.253 (0.205-0.392) | 0.653 | 1 | 0.239 (0.0755-0.468) | 0.276 (0.211-0.396) | 0.775 | 1 | 0.0832 (-0.0517-0.352) | -0.00154 (-0.0875-0.279) | 0.54 | 1 |  |  |
| Midazolam | -0.0486 (-0.176-0.367) | -0.0595 (-0.23-0.204) | 0.43 | 1 | -0.101 (-0.215-0.194) | -0.166 (-0.286-0.12) | 0.418 | 1 | -0.332 (-0.476--0.144) | -0.38 (-0.506--0.159) | 0.74 | 1 |  |  |
| Propofol | 0.235 (-0.0794-0.463) | -0.0794 (-0.172-0.0638) | 0.173 | 1 | -0.0265 (-0.0627-0.0792) | -0.108 (-0.126--0.0627) | 0.116 | 1 | -0.0581 (-0.616--0.00202) | -0.141 (-0.483--0.118) | 0.6 | 1 |  |  |
| **Name** | **Mean COx_L_a** | | | | **Mean COx_R_a** | | | | **% time ICP > 20** | | | |  |  |
|  | **Pre Dose** | **Post Dose** | **P value** | **Adj P Value** | **Pre Dose** | **Post Dose** | **P value** | **Adj P Value** | **Pre Dose** | **Post Dose** | **P value** | **Adj P Value** |  |  |
| Fentanyl | 0.0823 (0-0.218) | 0.0488 (-0.0472-0.177) | 0.0625 | 1 | 0.0693 (0-0.19) | 0.0332 (-0.0168-0.204) | 0.248 | 1 | 0 (0-9.68) | 0 (0-3.23) | 0.37 | 1 |  |  |
| Ketamine | 0 (0-0) | 0 (0-0.0123) | 0.663 | 1 | 0 (0-0.00415) | 0 (0-0.133) | 0.884 | 1 | 87.1 (0-87.1) | 74.2 (12.9-87.1) | 0.865 | 1 |  |  |
| Midazolam | 0.0756 (0-0.138) | 0.0233 (-0.0237-0.196) | 0.573 | 1 | 0.00658 (-0.0475-0.126) | 0.0142 (-0.0554-0.0908) | 0.557 | 1 | 0 (0-5.65) | 0 (0-0) | 0.382 | 1 |  |  |
| Propofol | 0.124 (0.124-0.349) | -0.000913 (-0.0315-0) | 0.0586 | 1 | 0.0172 (-0.00438-0.339) | -0.038 (-0.101--0.00438) | 0.249 | 1 | 0 (0-0) | 0 (0-0) | 1 | 1 |  |  |
| **Name** | **% time ICP > 22** | | | | **% time CPP > 60** | | | | **% time CPP > 70** | | | |  |  |
|  | **Pre Dose** | **Post Dose** | **P value** | **Adj P Value** | **Pre Dose** | **Post Dose** | **P value** | **Adj P Value** | **Pre Dose** | **Post Dose** | **P value** | **Adj P Value** |  |  |
| Fentanyl | 0 (0-0.806) | 0 (0-0) | 0.498 | 1 | 6.45 (0-12.9) | 9.68 (0-12.9) | 0.382 | 1 | 53.2 (12.9-87.1) | 48.4 (6.45-87.1) | 0.816 | 1 |  |  |
| Ketamine | 87.1 (0-87.1) | 24.2 (0-87.1) | 0.513 | 1 | 12.9 (6.45-41.9) | 12.9 (0-12.9) | 0.642 | 1 | 0 (0-90.3) | 58.1 (9.68-94.4) | 0.392 | 1 |  |  |
| Midazolam | 0 (0-4.84) | 0 (0-0) | 0.389 | 1 | 12.9 (0.806-12.9) | 12.9 (0-12.9) | 0.534 | 1 | 56.5 (12.9-89.5) | 83.9 (39.5-92.7) | 0.286 | 1 |  |  |
| Propofol | 0 (0-0) | 0 (0-0) | 1 | 1 | 12.9 (12.9-22.6) | 12.9 (12.9-22.6) | 1 | 1 | 80.6 (38.7-87.1) | 51.6 (38.7-87.1) | 0.914 | 1 |  |  |
| **Name** | **% time PRx > 0** | | | | **% time PRx > 0.25** | | | | **% time PRx > 0.35** | | | |  |  |
|  | **Pre Dose** | **Post Dose** | **P value** | **Adj P Value** | **Pre Dose** | **Post Dose** | **P value** | **Adj P Value** | **Pre Dose** | **Post Dose** | **P value** | **Adj P Value** |  |  |
| Fentanyl | 54.8 (27.4-83.9) | 51.6 (29-80.6) | 0.873 | 1 | 29 (4.84-61.3) | 25.8 (6.45-54.8) | 0.921 | 1 | 16.1 (3.23-48.4) | 17.7 (3.23-45.2) | 0.727 | 1 |  |  |
| Ketamine | 71 (58.1-87.1) | 75.8 (62.1-86.3) | 0.836 | 1 | 45.2 (29-83.9) | 58.1 (38.7-71) | 0.712 | 1 | 38.7 (12.9-74.2) | 51.6 (26.6-60.5) | 0.935 | 1 |  |  |
| Midazolam | 38.7 (20.2-76.6) | 38.7 (9.68-71) | 0.435 | 1 | 6.45 (3.23-61.3) | 16.1 (0-46) | 0.883 | 1 | 3.23 (0-56.5) | 8.06 (0-32.3) | 0.737 | 1 |  |  |
| Propofol | 61.3 (29-74.2) | 29 (25.8-51.6) | 0.139 | 1 | 51.6 (3.23-64.5) | 12.9 (3.23-35.5) | 0.401 | 1 | 48.4 (0-61.3) | 9.68 (0-19.4) | 0.389 | 1 |  |  |
| **Name** | **% time PAx > 0** | | | | **% time PAx > 0.25** | | | | **% time RAC > -0.1** | | | |  |  |
|  | **Pre Dose** | **Post Dose** | **P value** | **Adj P Value** | **Pre Dose** | **Post Dose** | **P value** | **Adj P Value** | **Pre Dose** | **Post Dose** | **P value** | **Adj P Value** |  |  |
| Fentanyl | 35.5 (16.1-74.2) | 38.7 (19.4-64.5) | 0.939 | 1 | 12.9 (0-35.5) | 12.9 (0-38.7) | 0.95 | 1 | 19.4 (9.68-51.6) | 25.8 (12.9-49.2) | 0.432 | 1 |  |  |
| Ketamine | 80.6 (71-87.1) | 80.6 (61.3-86.3) | 0.967 | 1 | 45.2 (35.5-83.9) | 58.1 (33.1-72.6) | 0.967 | 1 | 77.4 (58.1-100) | 64.5 (50-89.5) | 0.564 | 1 |  |  |
| Midazolam | 32.3 (6.45-72.6) | 27.4 (8.06-61.3) | 0.7 | 1 | 8.06 (0-34.7) | 8.06 (0-32.3) | 0.807 | 1 | 12.9 (12.9-53.2) | 16.1 (12.9-47.6) | 0.605 | 1 |  |  |
| Propofol | 38.7 (35.5-58.1) | 29 (22.6-35.5) | 0.173 | 1 | 9.68 (6.45-9.68) | 3.23 (0-6.45) | 0.338 | 1 | 54.8 (12.9-61.3) | 45.2 (12.9-58.1) | 0.829 | 1 |  |  |
| **Name** | **% time RAC > -0.05** | | | | **% time COx_R_a > 0** | | | | **% time COx_R_a > 0.3** | | | |  |  |
|  | **Pre Dose** | **Post Dose** | **P value** | **Adj P Value** | **Pre Dose** | **Post Dose** | **P value** | **Adj P Value** | **Pre Dose** | **Post Dose** | **P value** | **Adj P Value** |  |  |
| Fentanyl | 19.4 (9.68-41.9) | 22.6 (12.9-45.2) | 0.581 | 1 | 54.8 (32.3-74.2) | 51.6 (30.6-74.2) | 0.555 | 1 | 19.4 (6.45-37.1) | 19.4 (3.23-38.7) | 0.604 | 1 |  |  |
| Ketamine | 74.2 (58.1-96.8) | 58.1 (41.1-86.3) | 0.537 | 1 | 0 (0-48.4) | 0 (0-74.2) | 0.734 | 1 | 0 (0-19.4) | 0 (0-6.45) | 0.763 | 1 |  |  |
| Midazolam | 12.9 (10.5-44.4) | 12.9 (12.9-46.8) | 0.845 | 1 | 37.1 (32.3-64.5) | 48.4 (24.2-57.3) | 0.567 | 1 | 19.4 (3.23-31.5) | 9.68 (3.23-25) | 0.317 | 1 |  |  |
| Propofol | 51.6 (12.9-58.1) | 41.9 (12.9-45.2) | 0.449 | 1 | 54.8 (41.9-74.2) | 32.3 (29-45.2) | 0.249 | 1 | 3.23 (0-54.8) | 9.68 (6.45-12.9) | 1 | 1 |  |  |
| **Name** | **% time COx_L_a > 0** | | | | **% time COx_L_a > 0.3** | | | |  | | | |  |  |
|  | **Pre Dose** | **Post Dose** | **P value** | **Adj P Value** | **Pre Dose** | **Post Dose** | **P value** | **Adj P Value** |  |  |  |  |  |  |
| Fentanyl | 54.8 (35.5-74.2) | 51.6 (28.2-71) | 0.366 | 1 | 19.4 (6.45-38.7) | 19.4 (3.23-36.3) | 0.316 | 1 |  |  |  |  |  |  |
| Ketamine | 0 (0-48.4) | 0 (0-35.5) | 0.961 | 1 | 0 (0-9.68) | 0 (0-6.45) | 0.688 | 1 |  |  |  |  |  |  |
| Midazolam | 53.2 (28.2-65.3) | 41.9 (19.4-80.6) | 0.829 | 1 | 21 (6.45-32.3) | 12.9 (3.23-32.3) | 0.414 | 1 |  |  |  |  |  |  |
| Propofol | 64.5 (64.5-71) | 41.9 (32.3-48.4) | 0.292 | 1 | 32.3 (22.6-51.6) | 6.45 (0-16.1) | 0.168 | 1 |  |  |  |  |  |  |

*The table demonstrates the median and interquartile range of the pre/pose dose windows as well as the Wilcox signed ranked test between these windows with p-values adjusted using the Bonferroni analysis. The “change” indicates if the continuous infusion was increase/decrease or was a bolus dose. AMP, intracranial pulse amplitude; COx_R_a, cerebral oximetry index of right side using MAP; COx_L_a, cerebral oximetry index of left side using MAP; CPP, cerebral prefusion pressure; ICP, intracranial pressure; MAP, mean arterial blood pressure; PAx, pulse amplitude index; PRx, pressure reactivity; RAC, correlation between intracranial pulse amplitude and CPP; rSO2_L, regional oxygen saturation on left side; rSO2_R, regional oxygen saturation on right side.*

# Appendix I. Latent Profile Analysis - Norepinephrine

All LPA techniques are outline in the tiyLPA,^1^ as a summary the number of clusters was selected based on the optimal Bayesian information criteria. Then the selected number of clusters is used to demonstrate the data. As summary, LPA uses a specified number of clusters to categorize each data point into the optimal cluster, these optimal cluster are selected by minimizing the overall variance within each cluster and over the whole data. To select the optimal number of clusters, we leverage Bayesian information criteria (BIC) which balances model fit and overfitting by selecting the lowest BIC model. Then we demonstrated that LPA which corresponded to the indicated number of clusters.

*AMP, intracranial pulse amplitude; COx_R_a, cerebral oximetry index of right side using MAP; COx_L_a, cerebral oximetry index of left side using MAP; CPP, cerebral prefusion pressure; ICP, intracranial pressure; MAP, mean arterial blood pressure; PAx, pulse amplitude index; PRx, pressure reactivity; RAC, correlation between intracranial pulse amplitude and CPP; rSO2_L, regional oxygen saturation on left side; rSO2_R, regional oxygen saturation on right side.*

1. Rosenberg JM, Beymer PN, Anderson DJ, Lissa C j van, Schmidt JA. tidyLPA: An R Package to Easily Carry Out Latent Profile Analysis (LPA) Using Open-Source or Commercial Software. *Journal of Open Source Software*. 2019;3(30):978. doi:10.21105/joss.00978

## Appendix I1. Decrease in Agent - MAP/ICP/CPP/AMP/rSO2_l/rSO2_r


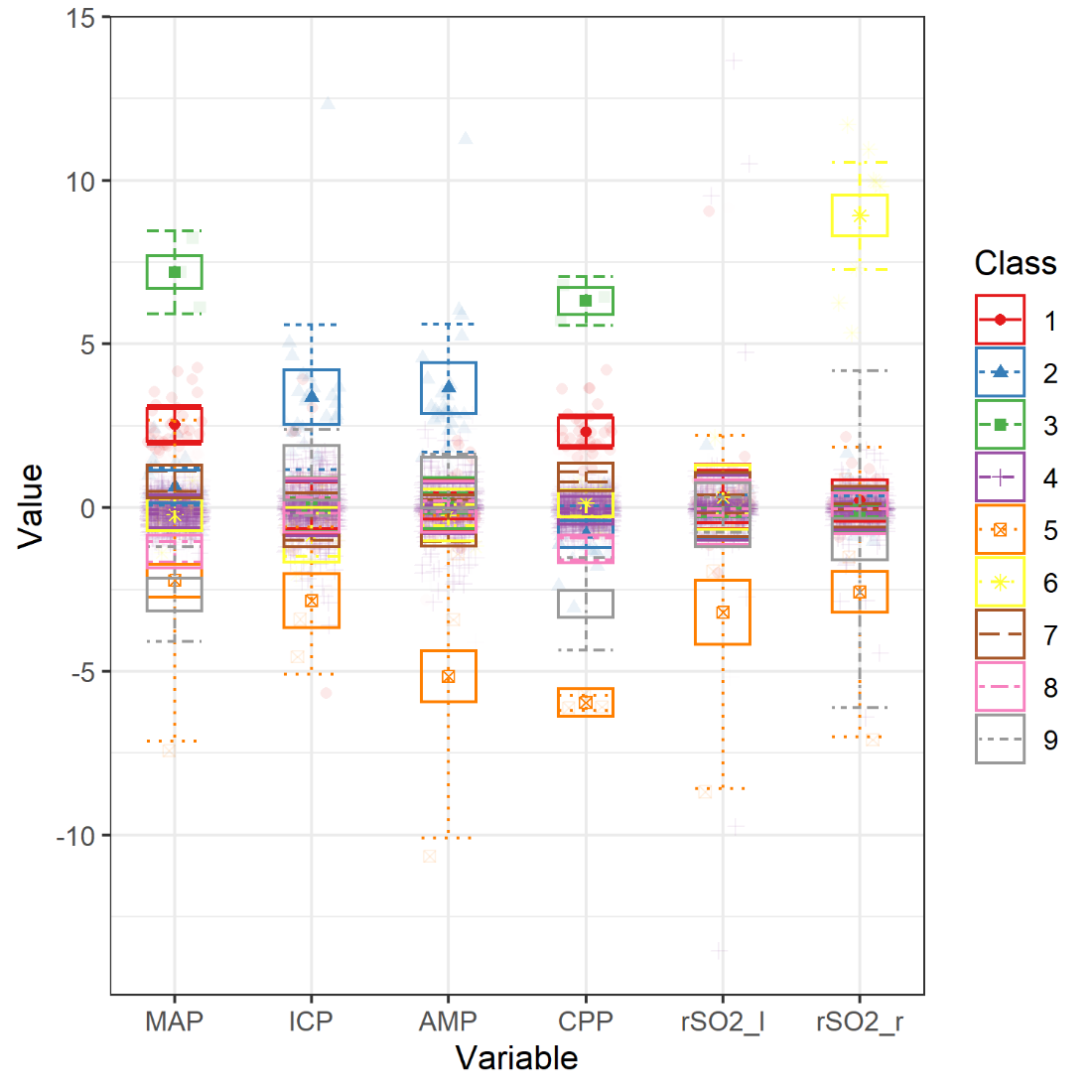


## Appendix I2. Decrease in Agent - PRx/PAx/RAC/COx_R_a /COx_L_a


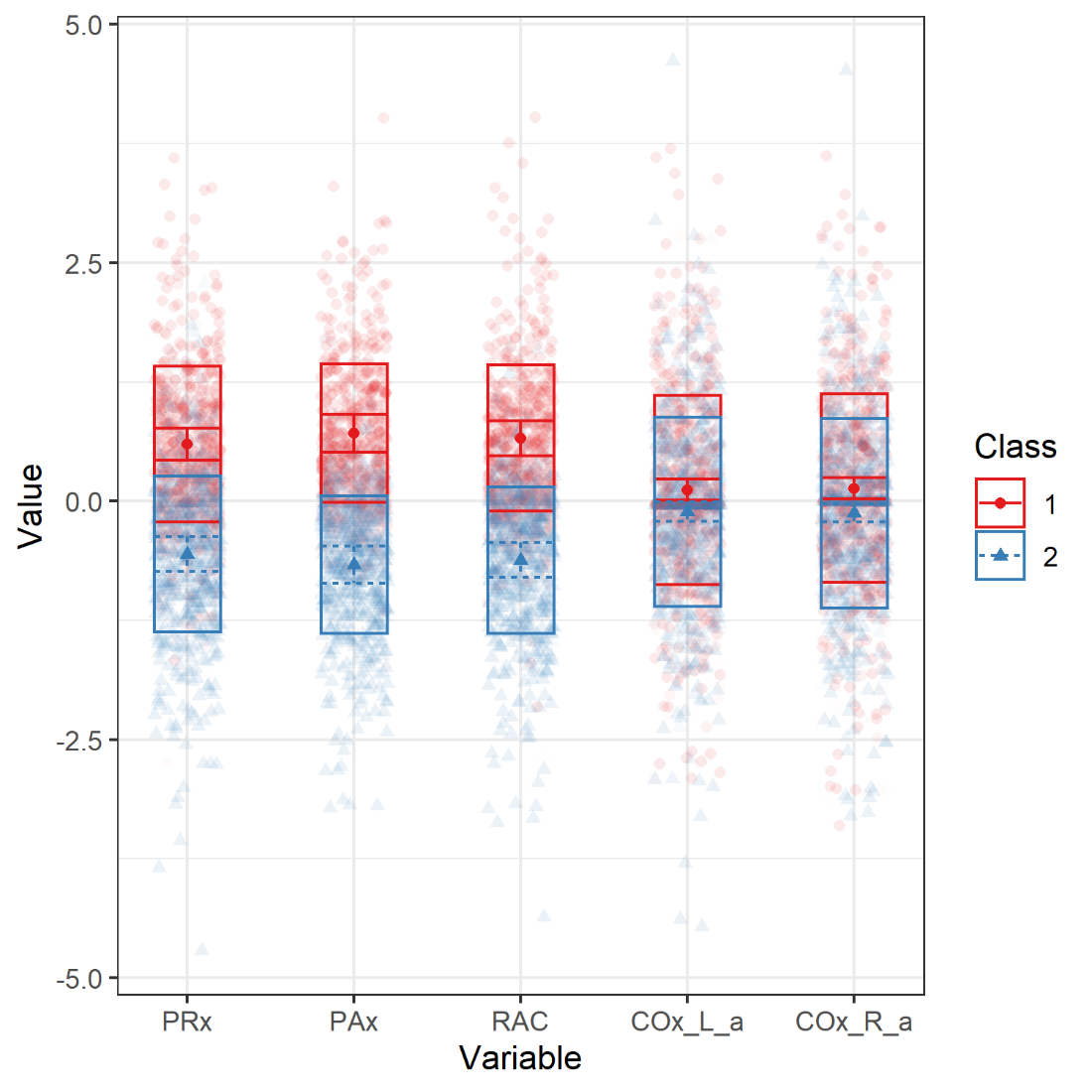


## Appendix I3. Increase in Agent - MAP/ICP/CPP/AMP/rSO2_l/rSO2_r


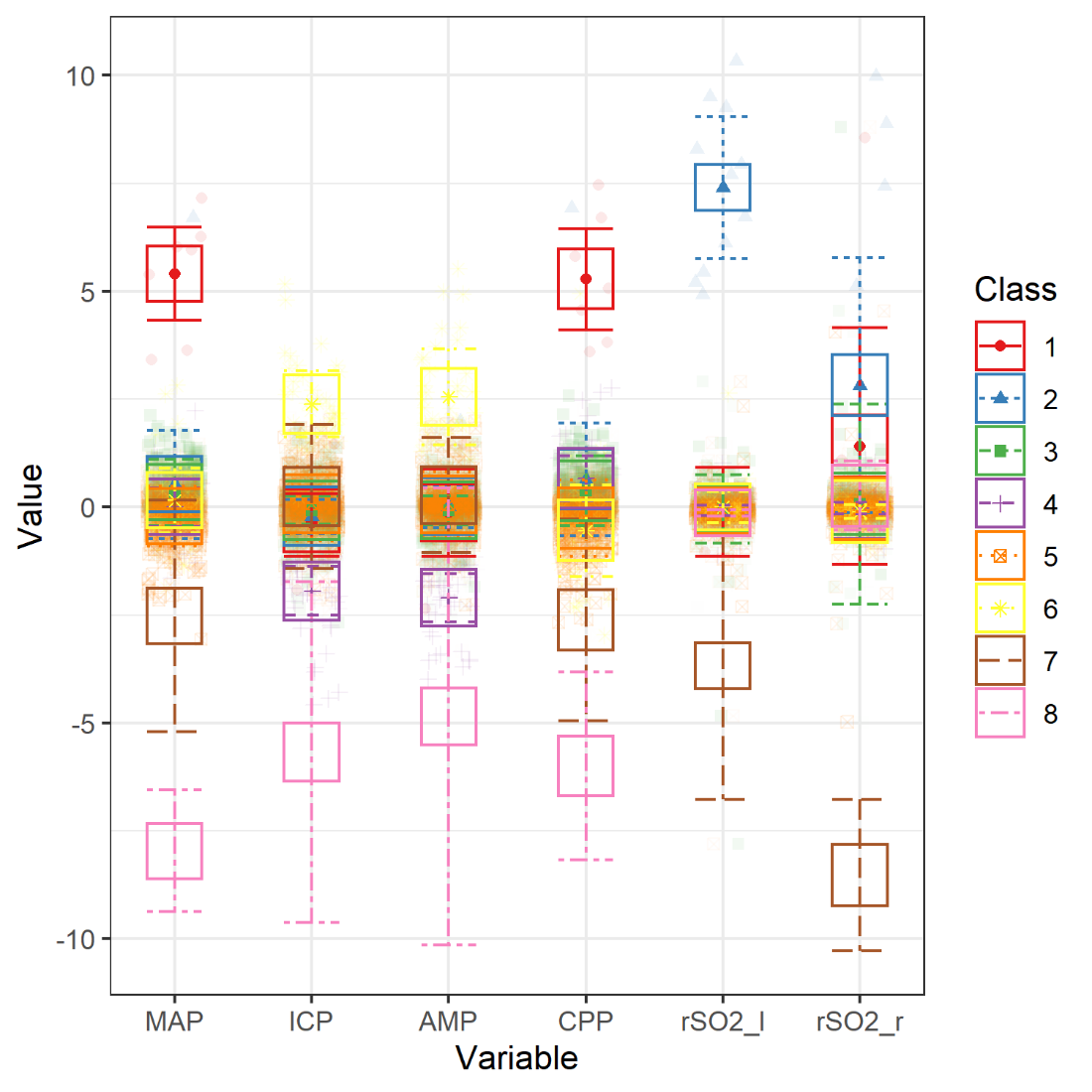


## Appendix I4. Increase in Agent - PRx/PAx/RAC/COx_R_a /COx_L_a


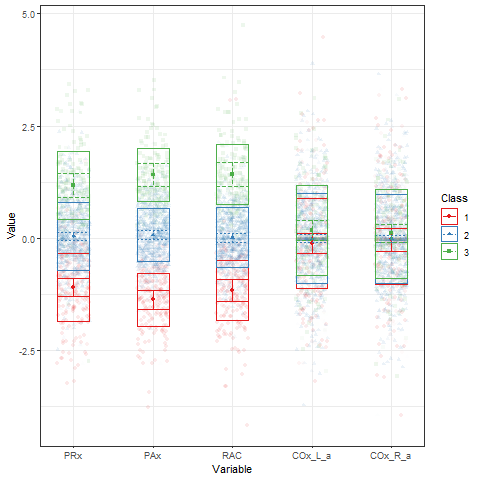


# Appendix J. Latent Profile Analysis - Propofol

All LPA techniques are outline in the tiyLPA,^1^ as a summary the number of clusters was selected based on the optimal Bayesian information criteria. Then the selected number of clusters is used to demonstrate the data. As summary, LPA uses a specified number of clusters to categorize each data point into the optimal cluster, these optimal cluster are selected by minimizing the overall variance within each cluster and over the whole data. To select the optimal number of clusters, we leverage Bayesian information criteria (BIC) which balances model fit and overfitting by selecting the lowest BIC model. Then we demonstrated that LPA which corresponded to the indicated number of clusters.

*AMP, intracranial pulse amplitude; COx_R_a, cerebral oximetry index of right side using MAP; COx_L_a, cerebral oximetry index of left side using MAP; CPP, cerebral prefusion pressure; ICP, intracranial pressure; MAP, mean arterial blood pressure; PAx, pulse amplitude index; PRx, pressure reactivity; RAC, correlation between intracranial pulse amplitude and CPP; rSO2_L, regional oxygen saturation on left side; rSO2_R, regional oxygen saturation on right side.*

1. Rosenberg JM, Beymer PN, Anderson DJ, Lissa C j van, Schmidt JA. tidyLPA: An R Package to Easily Carry Out Latent Profile Analysis (LPA) Using Open-Source or Commercial Software. *Journal of Open Source Software*. 2019;3(30):978. doi:10.21105/joss.00978

## Appendix J1. Decrease in Agent - MAP/ICP/CPP/AMP/rSO2_l/rSO2_r


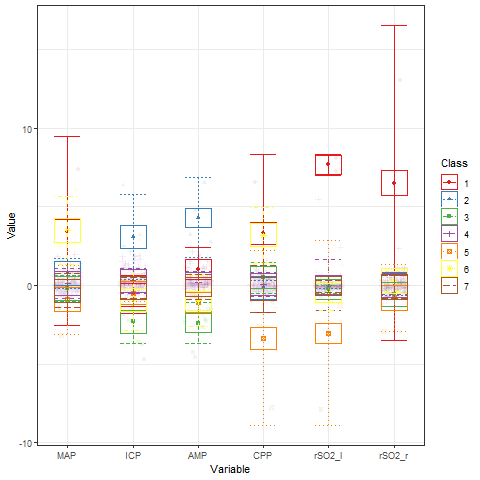


## Appendix J2. Decrease in Agent - PRx/PAx/RAC/COx_R_a /COx_L_a


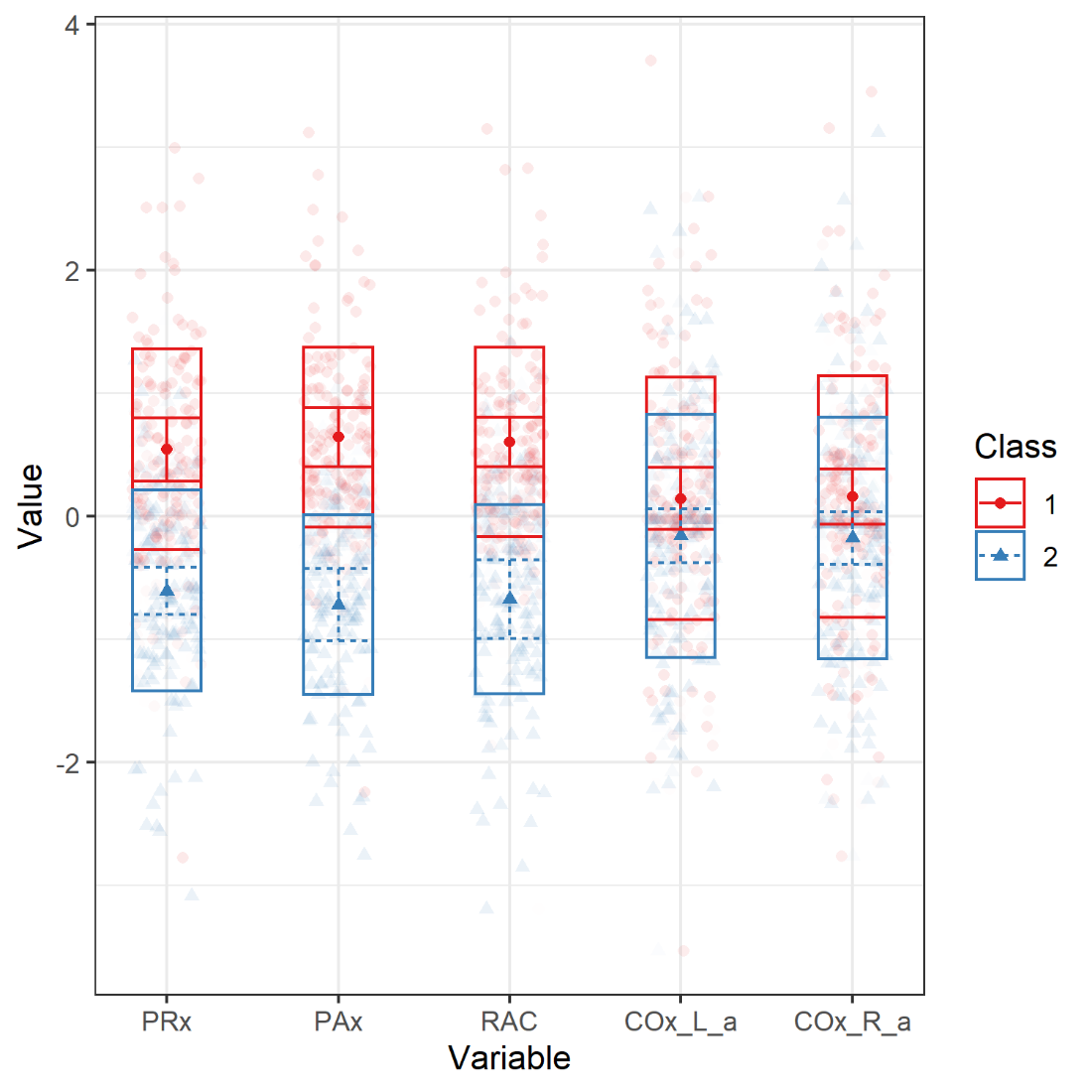


## Appendix J3. Increase in Agent - MAP/ICP/CPP/AMP/rSO2_l/rSO2_r


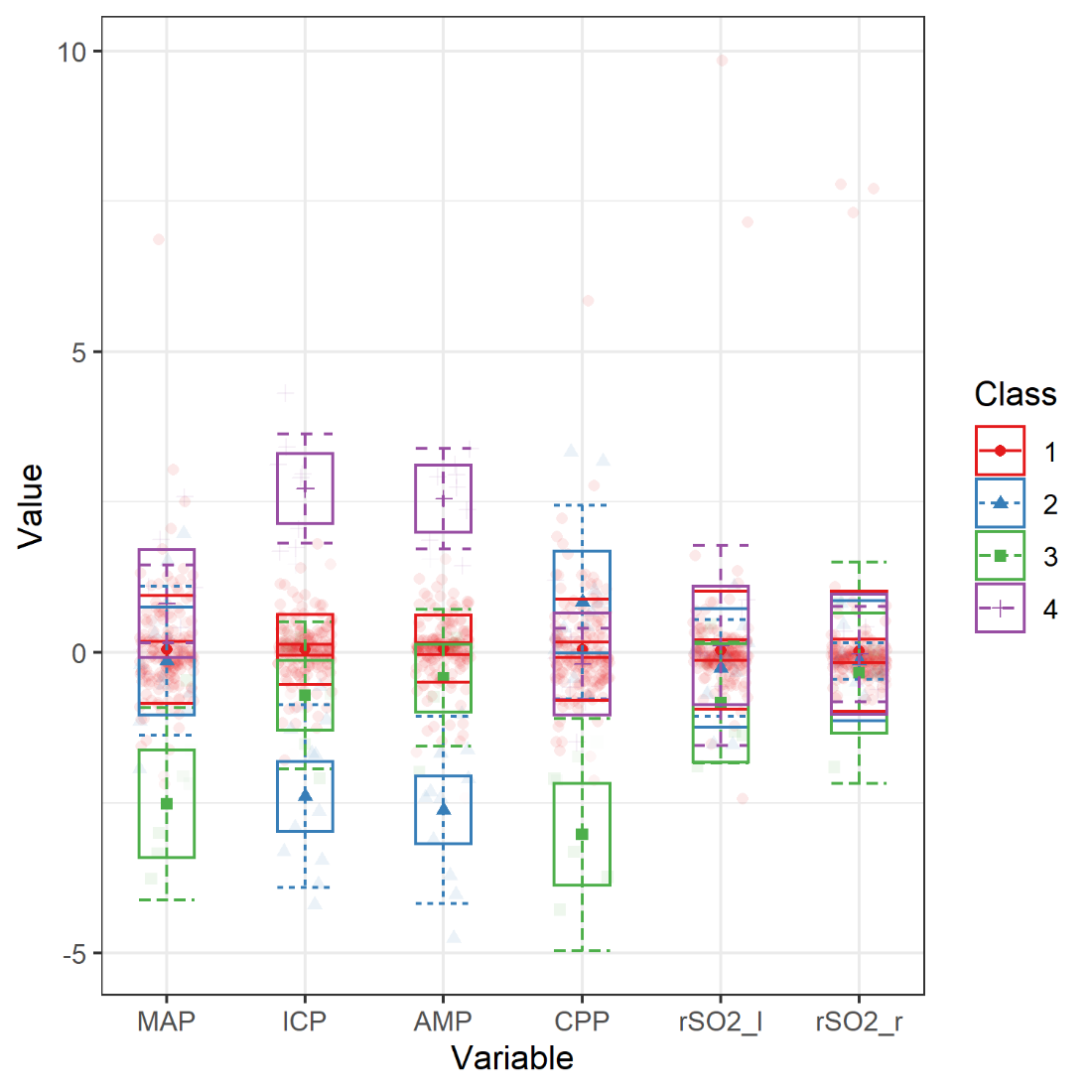


## Appendix J4. Increase in Agent - PRx/PAx/RAC/COx_R_a /COx_L_a


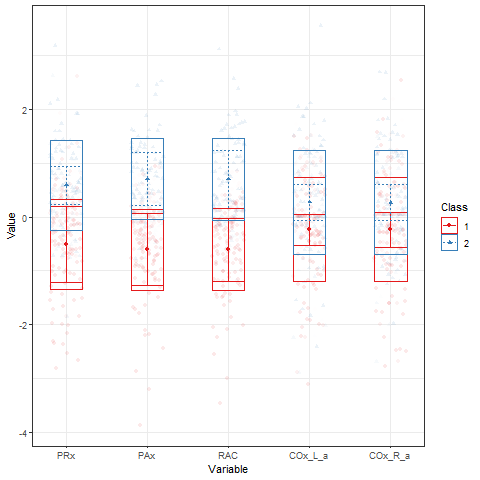


# Appendix K. Norepinephrine Impulse Response Functions

To perform an impulse response function, first a vector autoregressive model was created between MAP and ICP/rSO_2_/PRx/PAx for both the pre/post windows (30 minutes of data). Then a pulse was given between MAP and the other variables and vice versa to give a resulting 10-minute pulse response. For all dose changes, these were found then a final plot was created for the median (black line) and interquartile values (blue lines) for each value at each minute.

*AMP, intracranial pulse amplitude; COx_R_a, cerebral oximetry index of right side using MAP; COx_L_a, cerebral oximetry index of left side using MAP; CPP, cerebral prefusion pressure; ICP, intracranial pressure; MAP, mean arterial blood pressure; PAx, pulse amplitude index; PRx, pressure reactivity; RAC, correlation between intracranial pulse amplitude and CPP; rSO2_L, regional oxygen saturation on left side; rSO2_R, regional oxygen saturation on right side.*

## Appendix K1. Increase in Agent


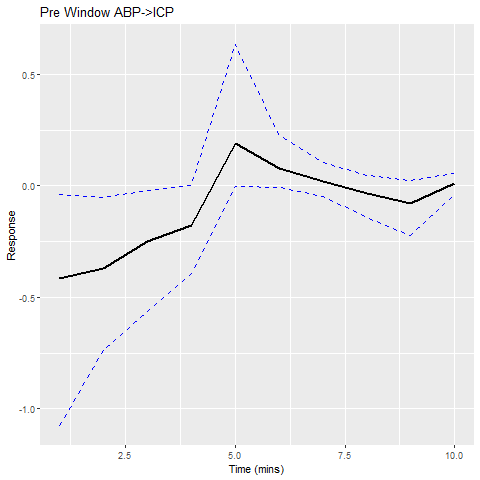

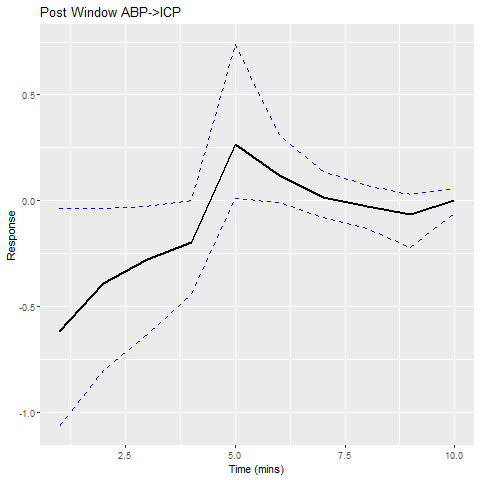


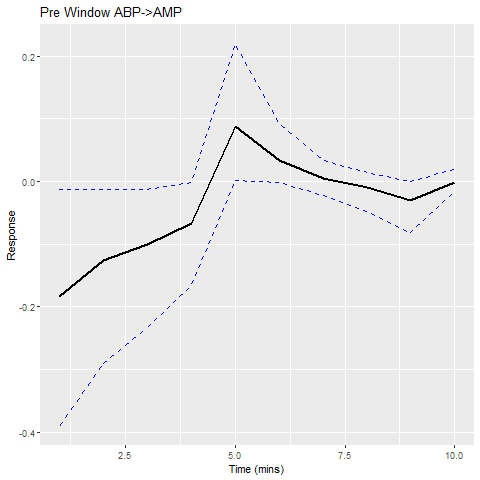

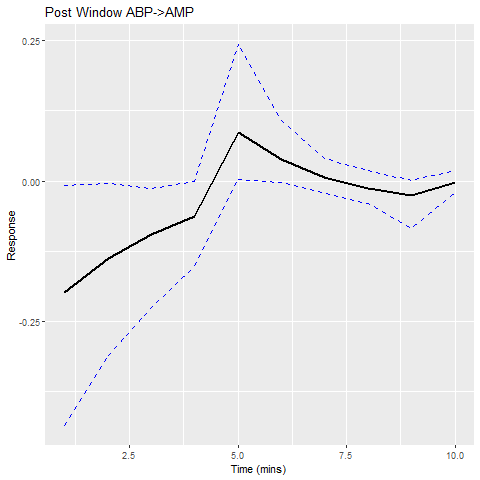

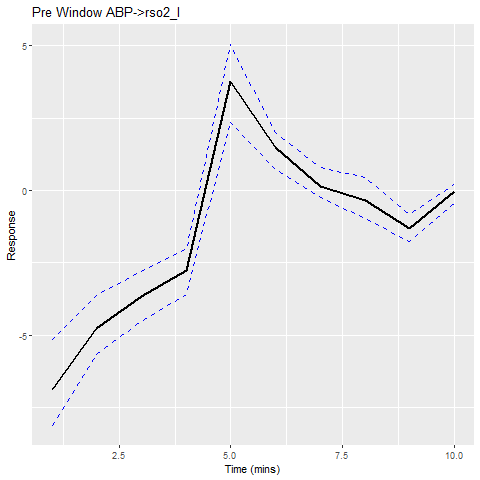

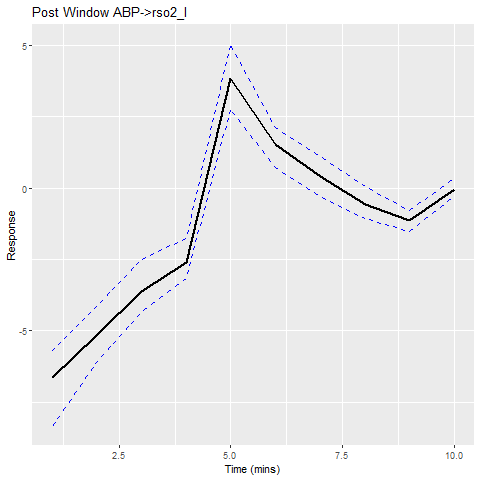

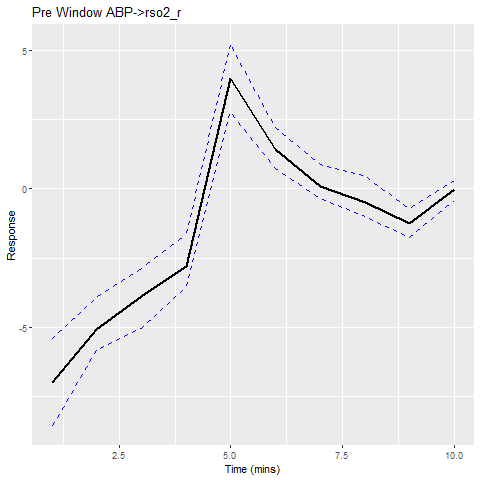

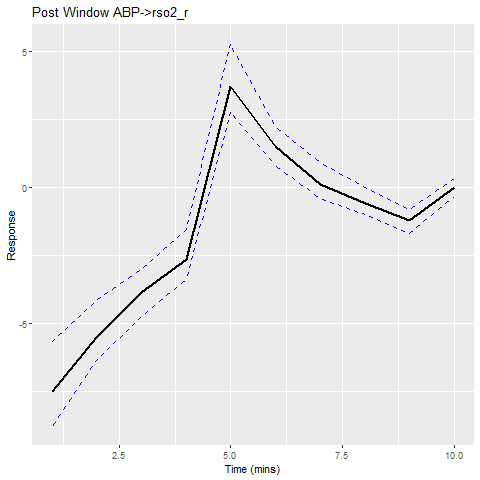

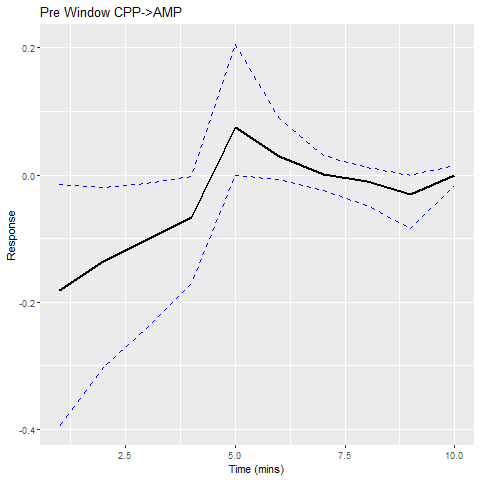

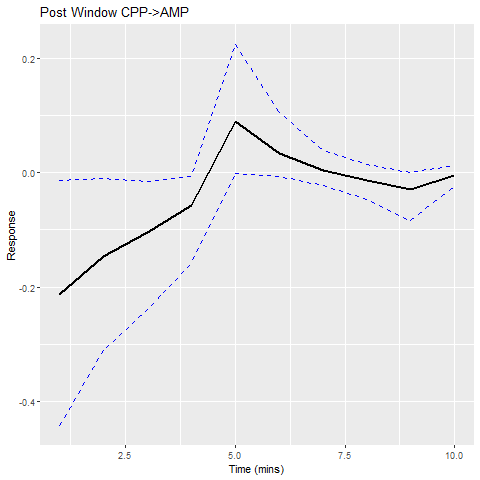

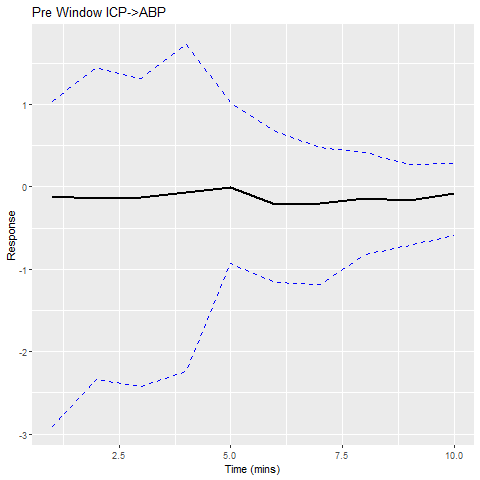

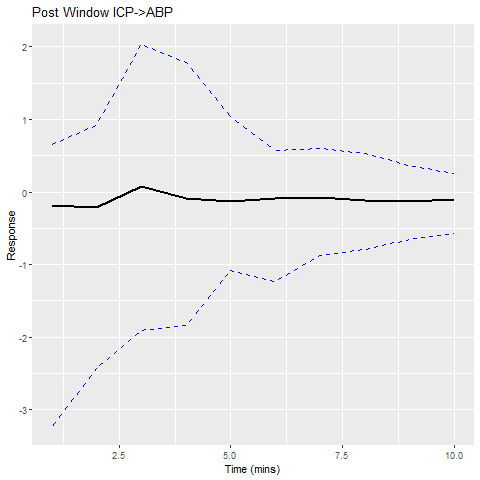

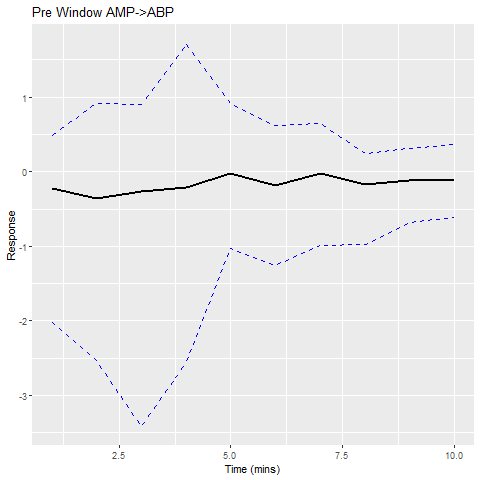

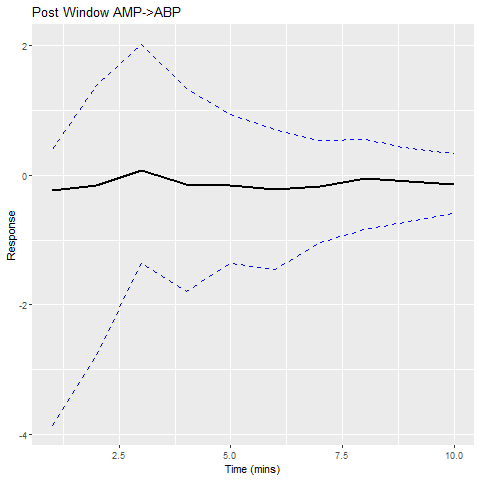

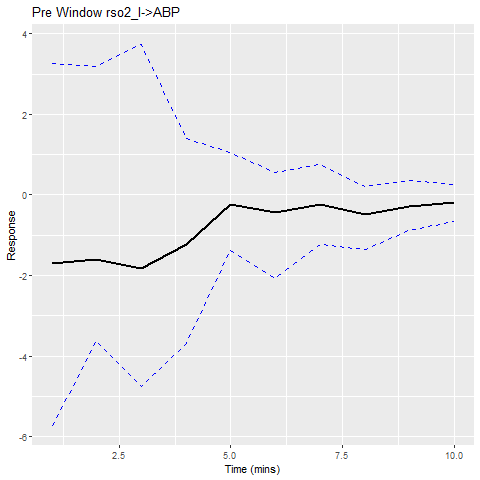

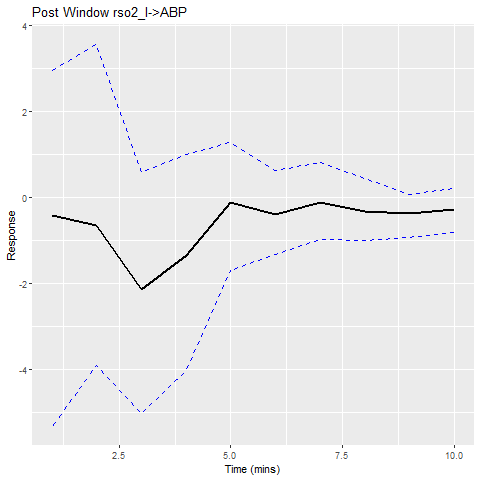

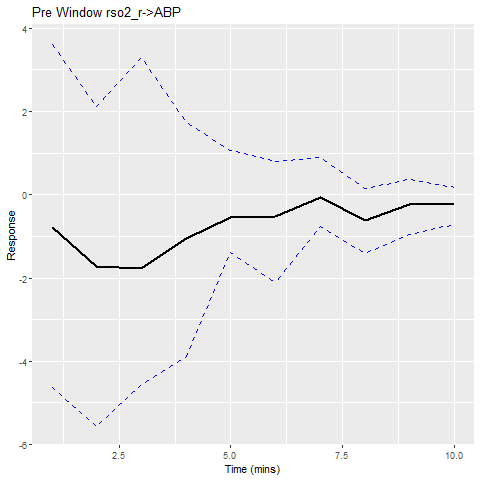

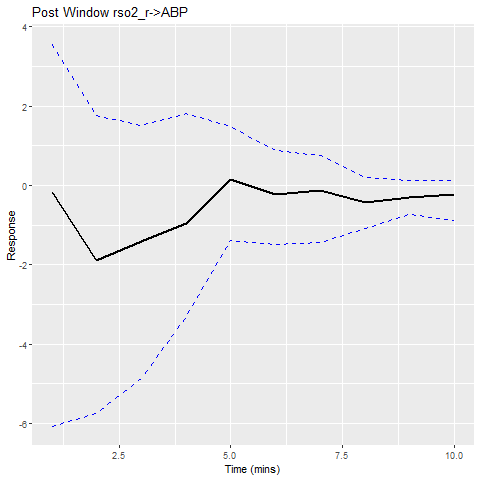

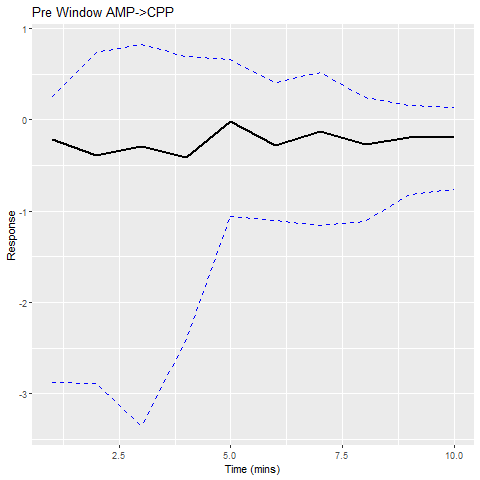

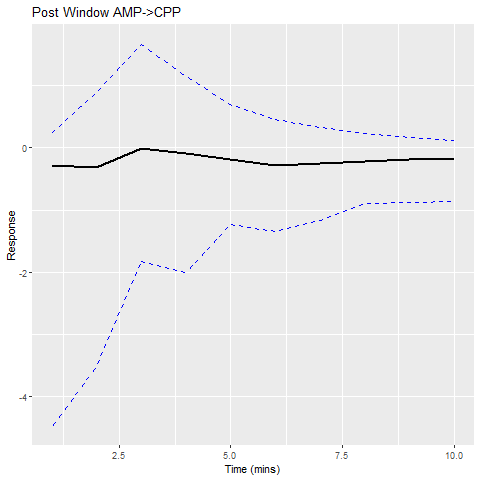


## Appendix K2. Decrease in Agent


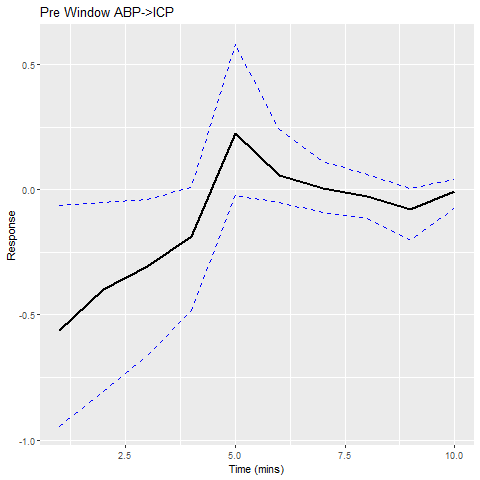

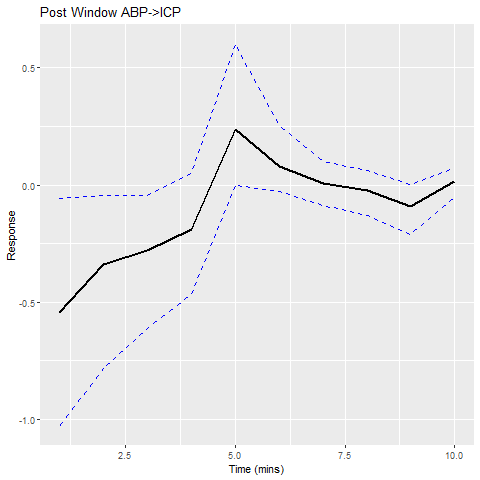


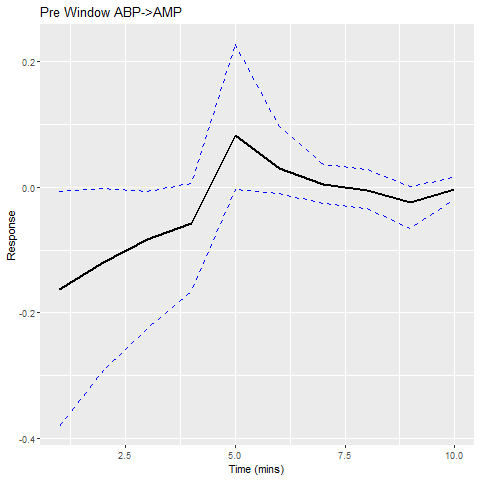

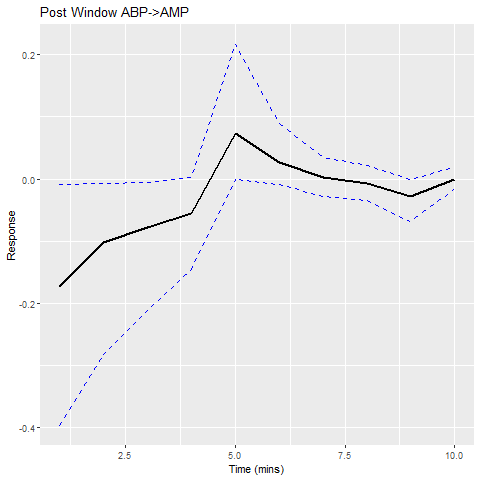


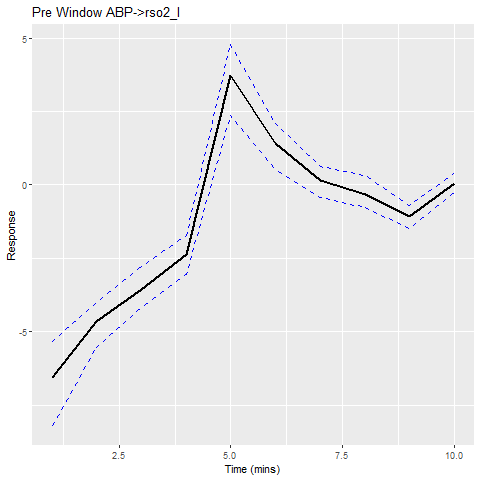

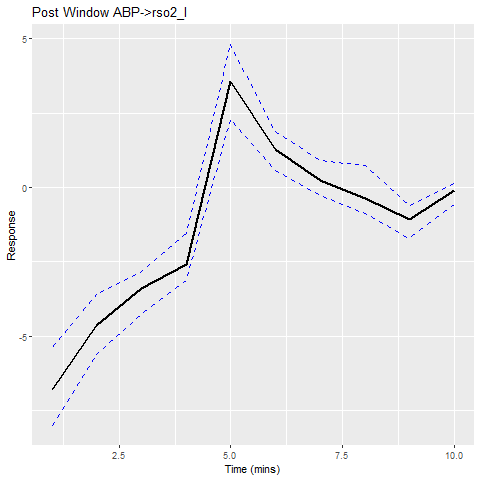

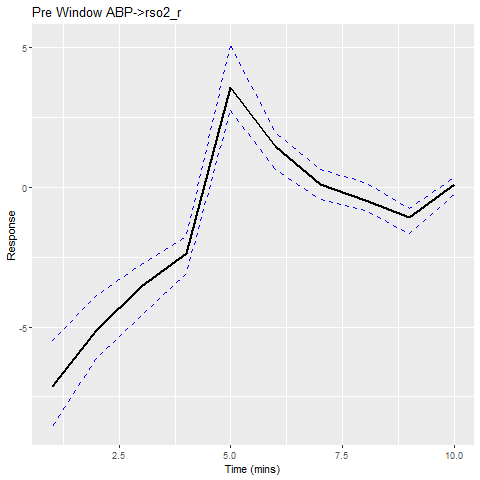

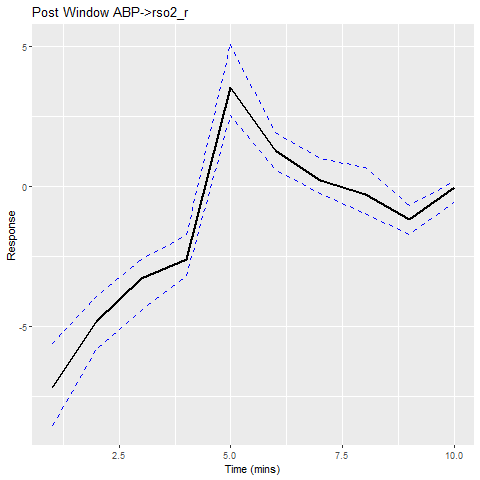

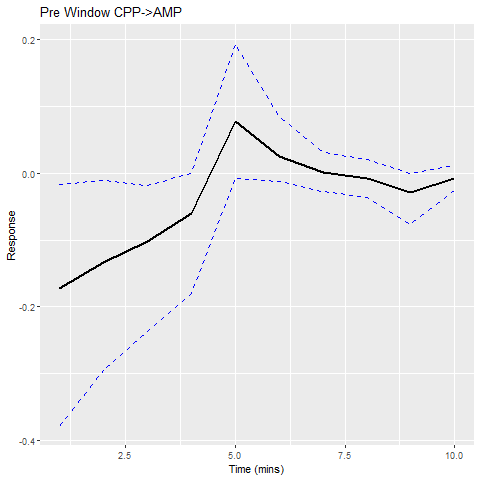

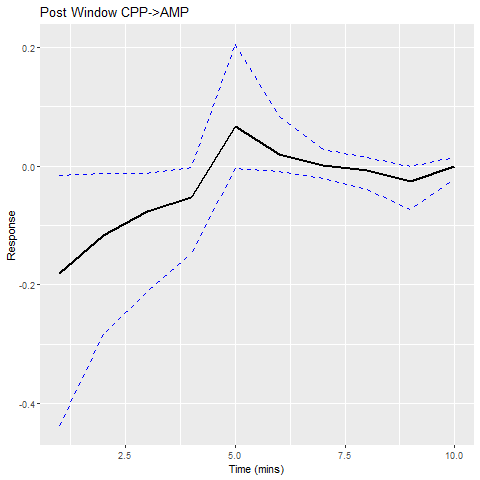

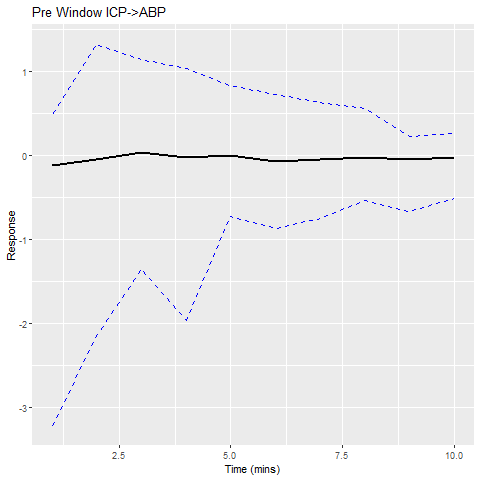

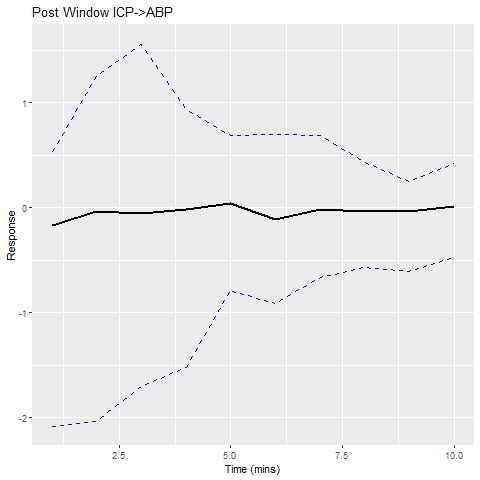

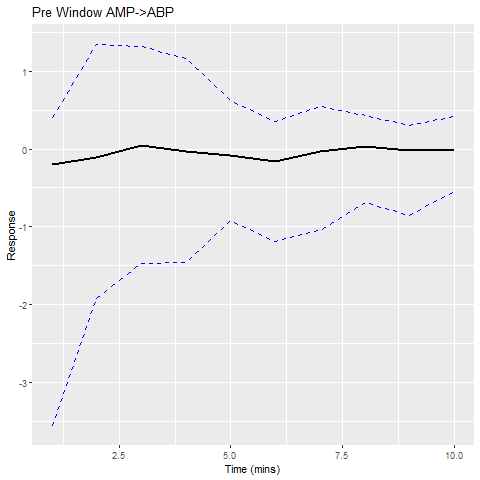

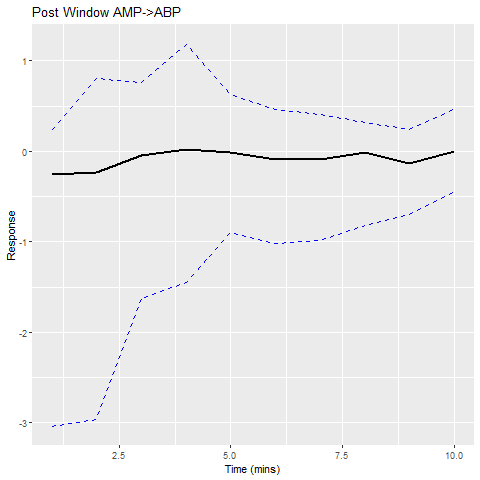

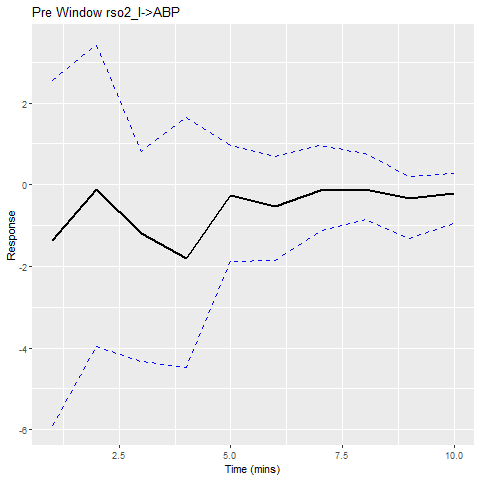

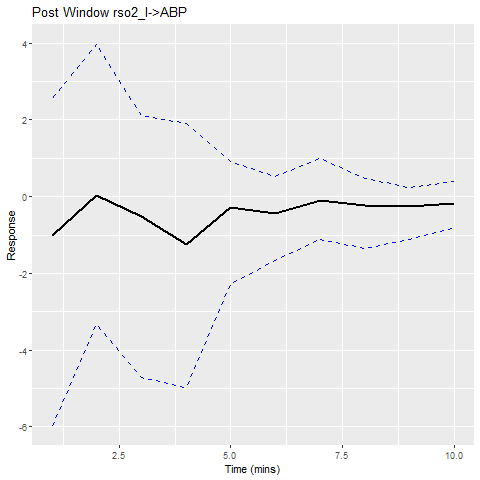

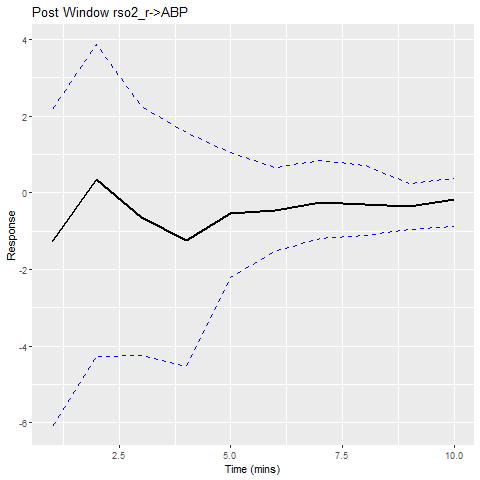

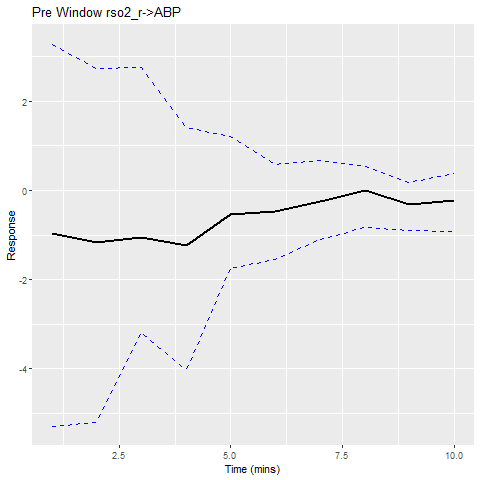

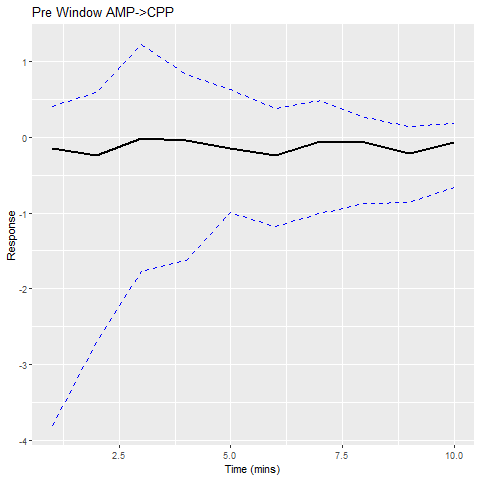

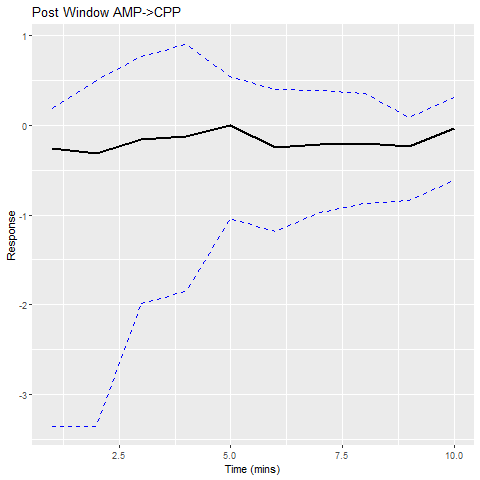


# Appendix L. Propofol Impulse Response Functions

To perform an impulse response function, first a vector autoregressive model was created between MAP and ICP/rSO_2_/PRx/PAx for both the pre/post windows (30 minutes of data). Then a pulse was given between MAP and the other variables and vice versa to give a resulting 10-minute pulse response. For all dose changes, these were found then a final plot was created for the median (black line) and interquartile values (blue lines) for each value at each minute.

*AMP, intracranial pulse amplitude; COx_R_a, cerebral oximetry index of right side using MAP; COx_L_a, cerebral oximetry index of left side using MAP; CPP, cerebral prefusion pressure; ICP, intracranial pressure; MAP, mean arterial blood pressure; PAx, pulse amplitude index; PRx, pressure reactivity; RAC, correlation between intracranial pulse amplitude and CPP; rSO2_L, regional oxygen saturation on left side; rSO2_R, regional oxygen saturation on right side.*

## Appendix L1. Increase in Agent


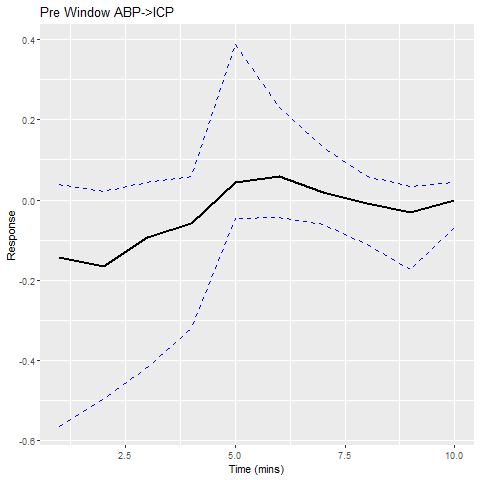

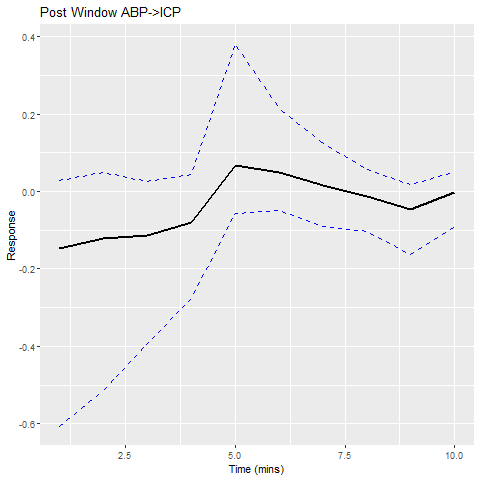


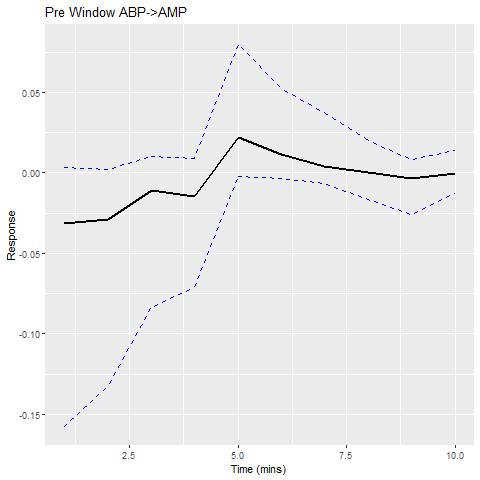

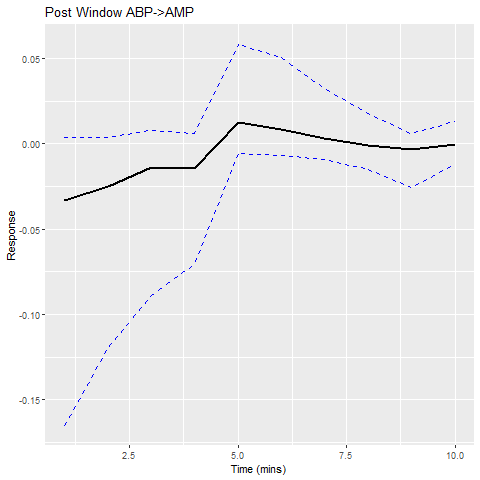


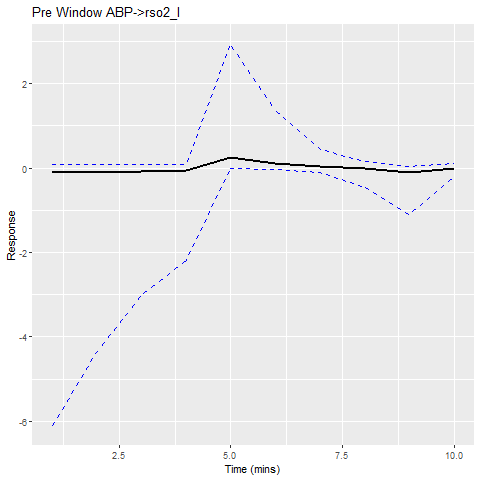

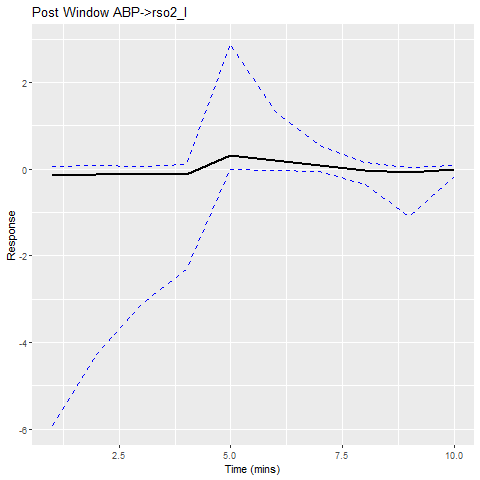

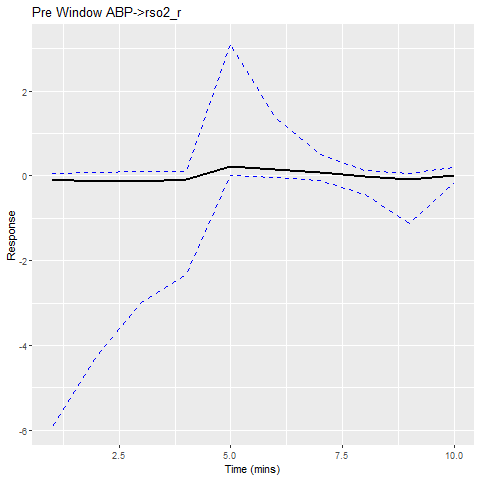

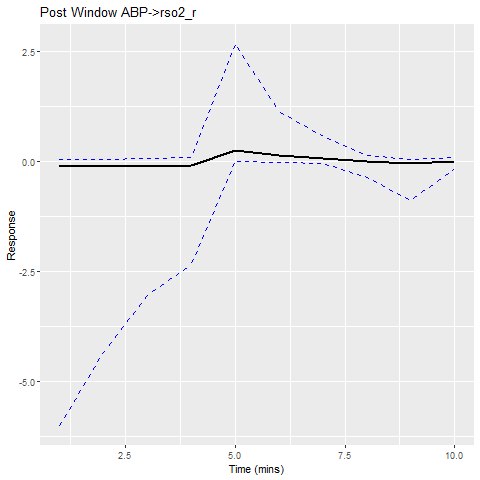

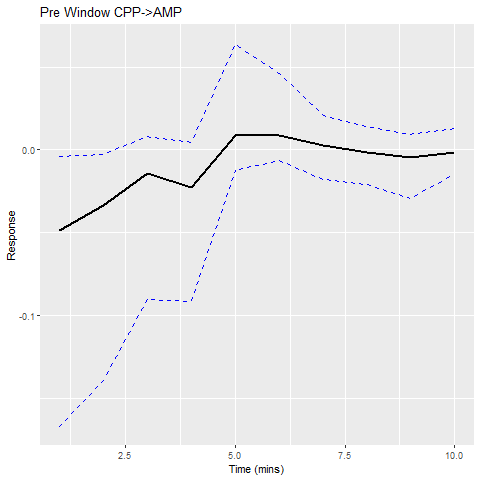

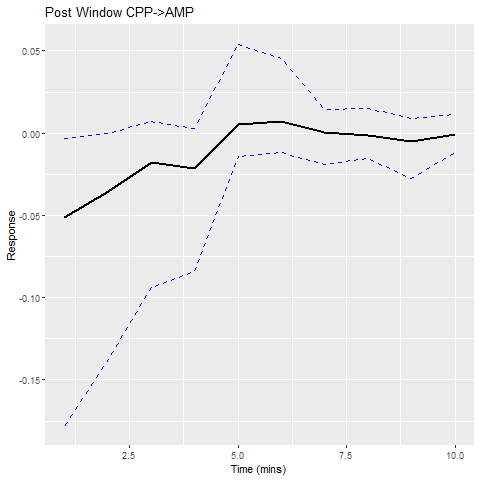

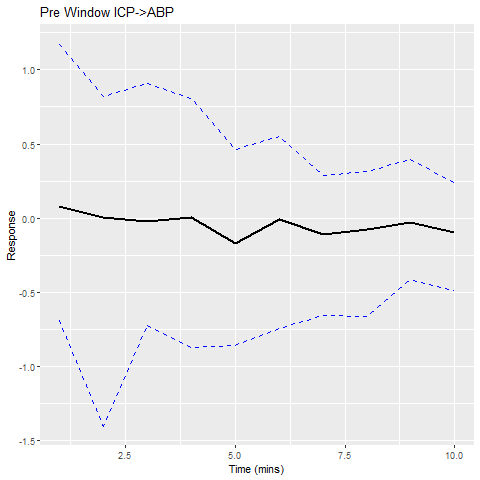

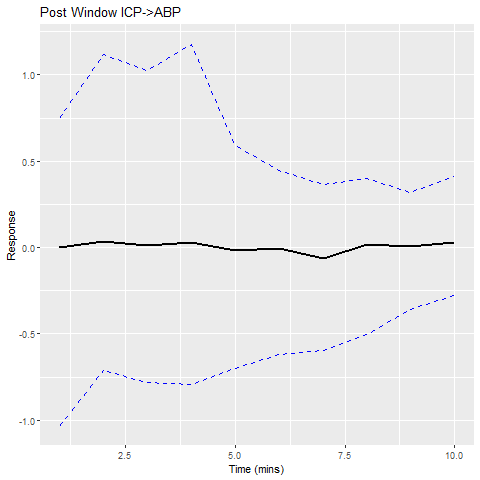

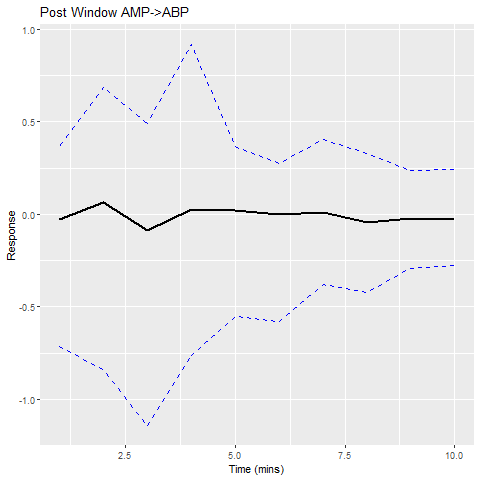

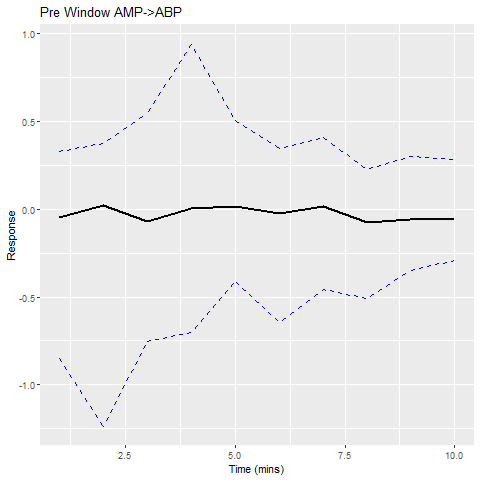

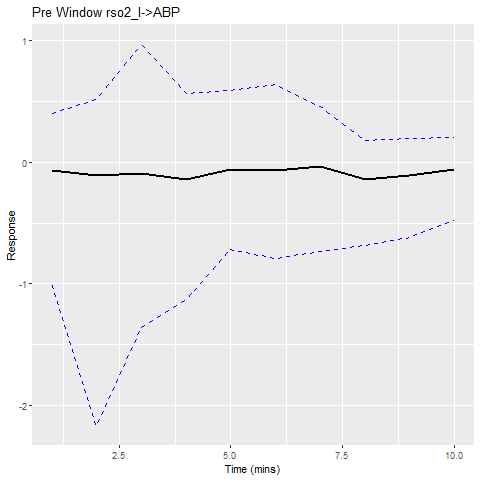

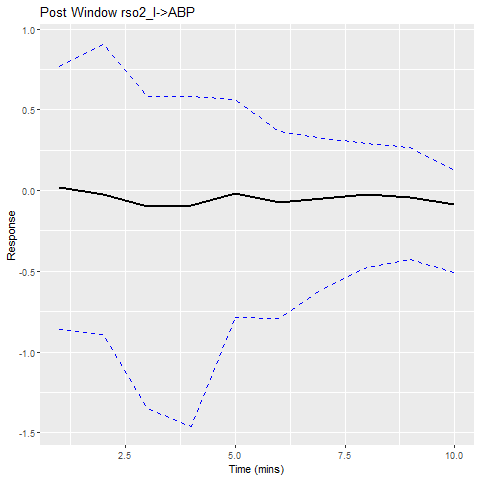

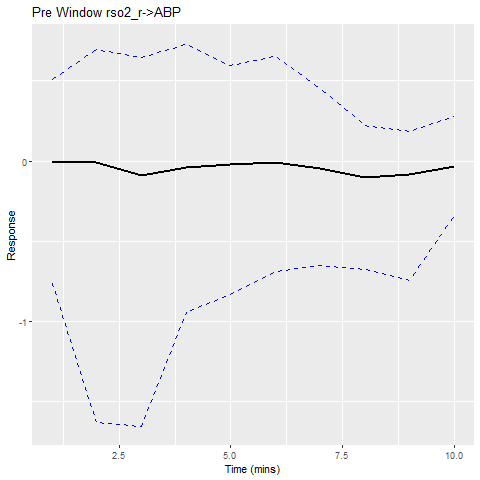

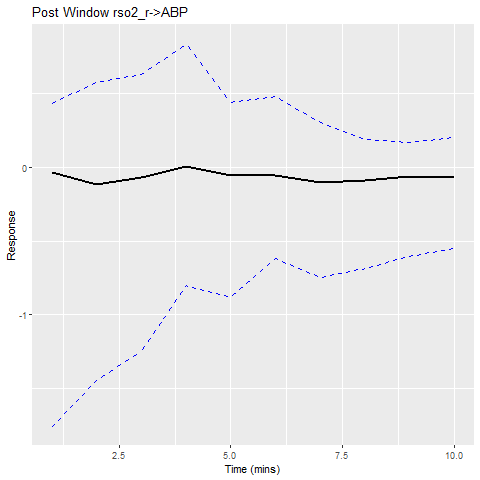

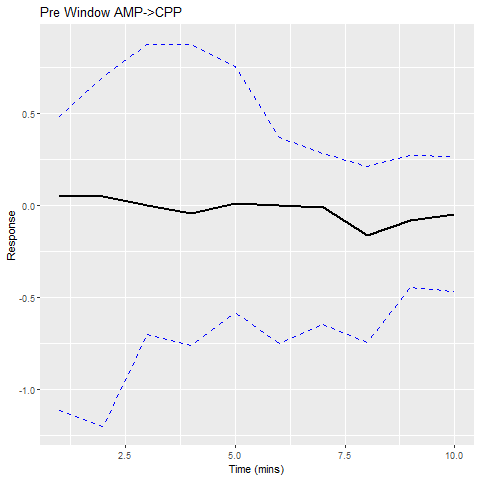

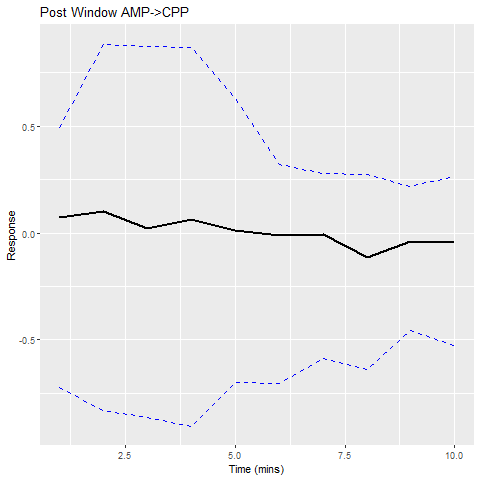


## Appendix L2. Decrease in Agent

*
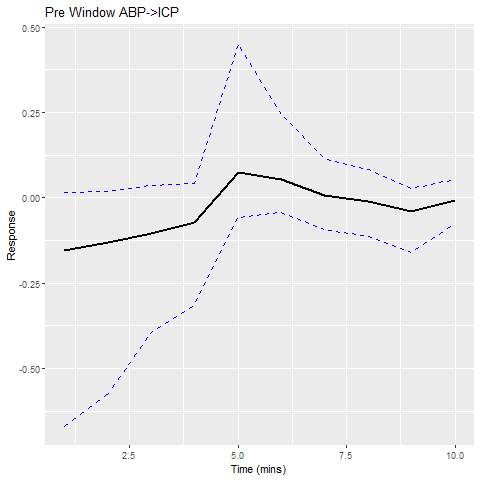

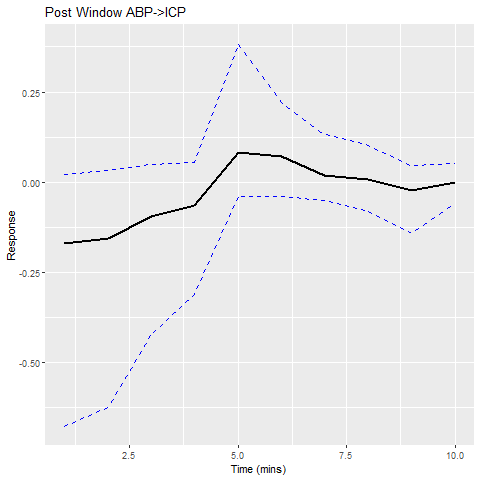
*

*
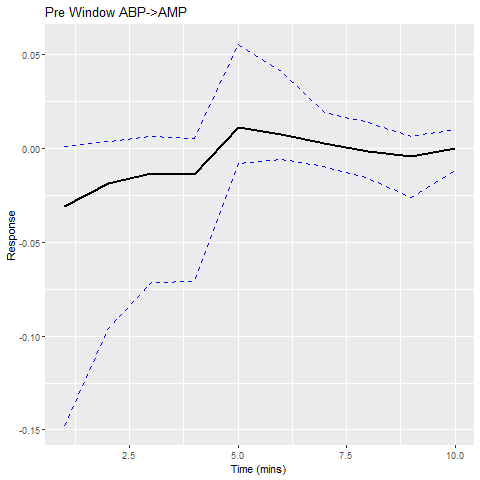

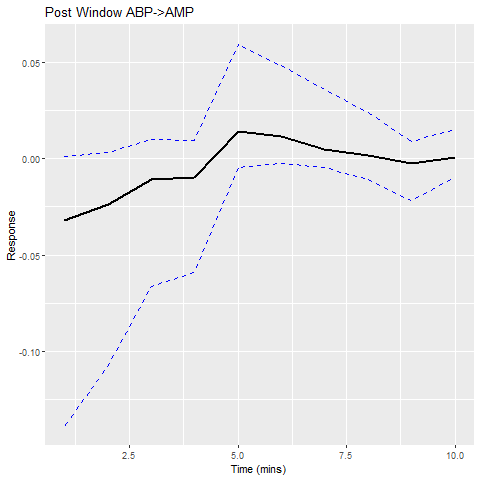
*

*
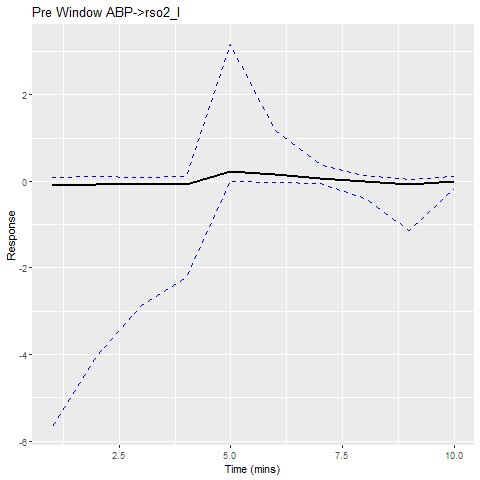

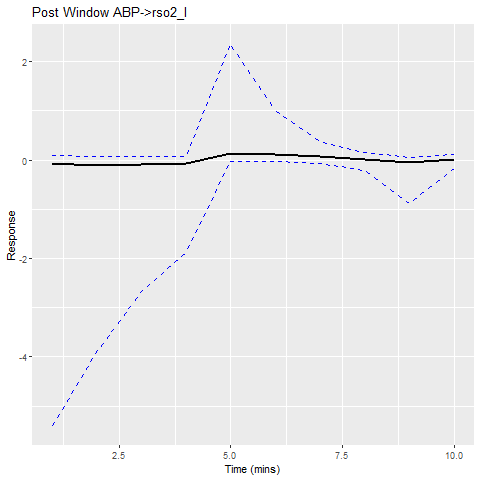

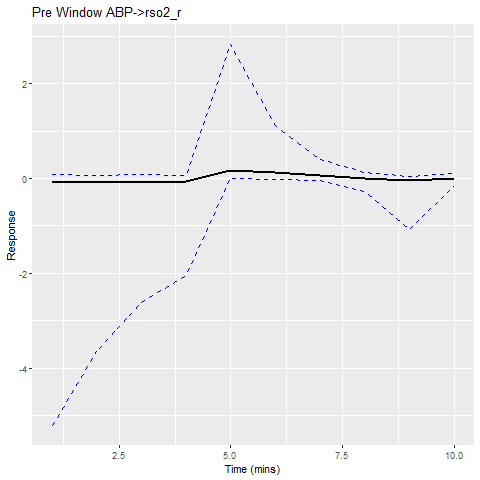

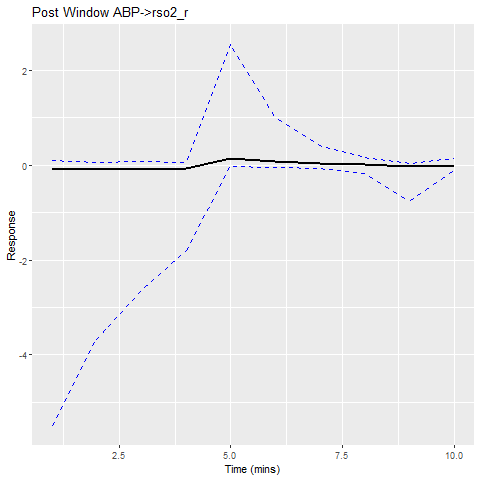

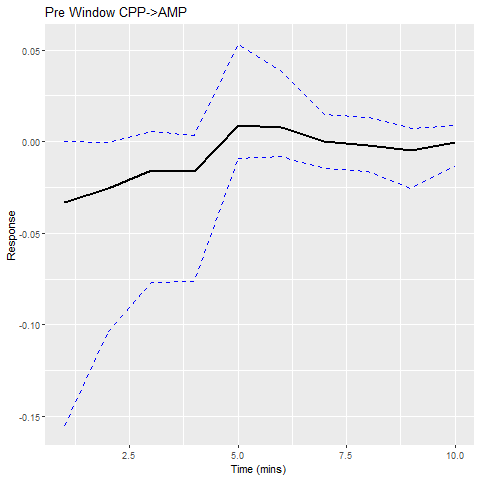

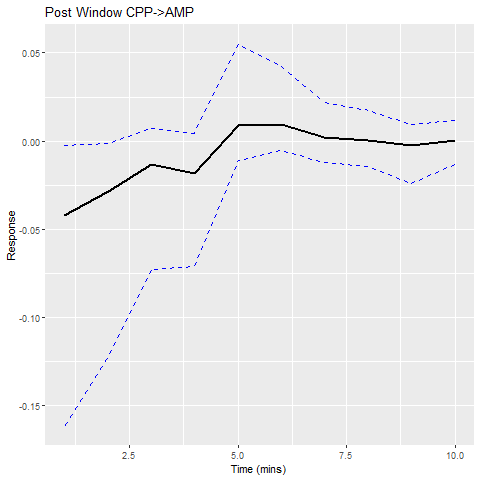

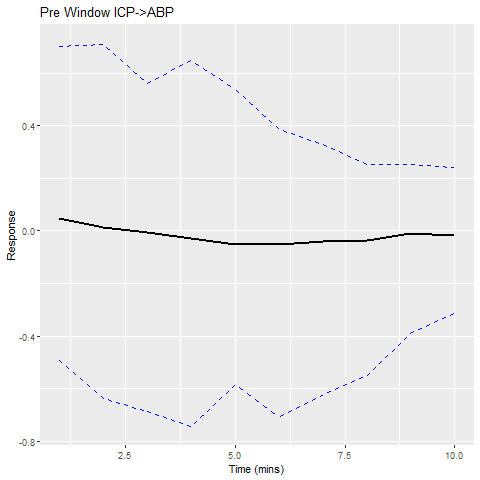

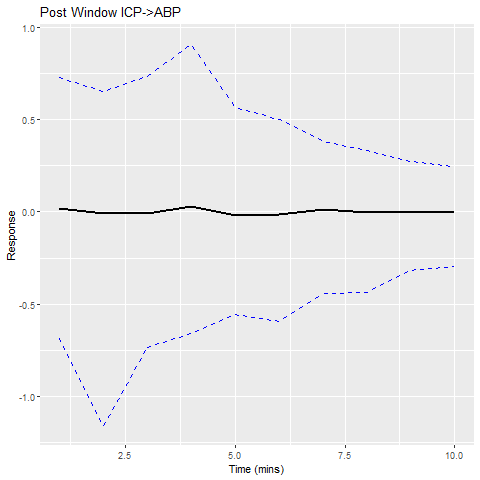

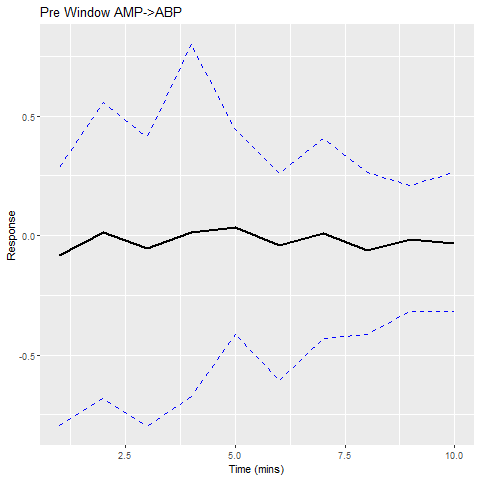
*

# Appendix M. Fentanyl Impulse Response Functions

To perform an impulse response function, first a vector autoregressive model was created between MAP and ICP/rSO_2_/PRx/PAx for both the pre/post windows (30 minutes of data). Then a pulse was given between MAP and the other variables and vice versa to give a resulting 10-minute pulse response. For all dose changes, these were found then a final plot was created for the median (black line) and interquartile values (blue lines) for each value at each minute.

*AMP, intracranial pulse amplitude; COx_R_a, cerebral oximetry index of right side using MAP; COx_L_a, cerebral oximetry index of left side using MAP; CPP, cerebral prefusion pressure; ICP, intracranial pressure; MAP, mean arterial blood pressure; PAx, pulse amplitude index; PRx, pressure reactivity; RAC, correlation between intracranial pulse amplitude and CPP; rSO2_L, regional oxygen saturation on left side; rSO2_R, regional oxygen saturation on right side.*

## Appendix M1. Bolus
